# Origins of adaptive genomic variation in a wild rodent

**DOI:** 10.1038/s42003-026-10511-6

**Published:** 2026-06-26

**Authors:** Marcos O. R. da Cruz, Silvia Marková, Marco A. Escalante, Jeremy B. Searle, Petr Kotlík, Hayley C. Lanier

**Affiliations:** 1 Sam Noble Museum, University of Oklahoma, Norman, OK USA; 2 School of Biological Sciences, University of Oklahoma, Norman, OK USA; 3 Laboratory of Molecular Ecology, Institute of Animal Physiology and Genetics of the Czech Academy of Sciences, Liběchov, Czechia; 4 Department of Ecology and Evolutionary Biology, Cornell University, Ithaca, NY USA

**Keywords:** Evolutionary biology, Genomics

## Abstract

Population history is a key yet underexplored determinant of local adaptation. Here, we assess its role by inferring the geographic origin of alleles at climate-associated loci. Using whole-genome data from 151 bank voles (*Clethrionomys glareolus*) sampled in Britain and four mainland European source populations with ancestry representing the Carpathian and Western refugia, we evaluate the origins of SNPs linked to climate adaptation at 1074 candidate loci. Most SNPs at adaptive loci represent widespread polymorphisms, shared across both European refugia and Britain. However, the larger and older Carpathian refugium contributed more unique SNPs associated with climate adaptation, which are more positively correlated with high temperatures, compared to the smaller contribution of uniquely Western alleles. Together, these results demonstrate that local adaptation is shaped not only by shared ancestral variation but also the distinct evolutionary legacies of individual refugia.

**Figure:**
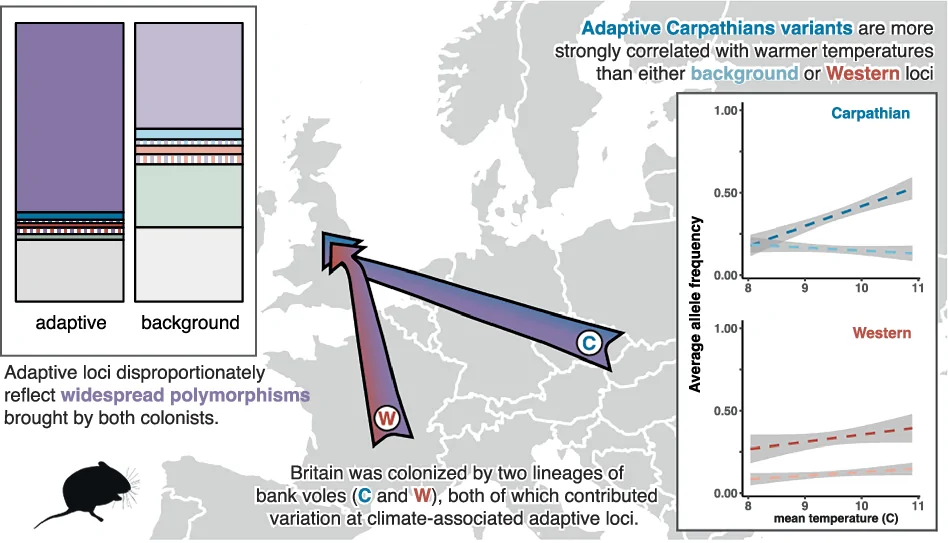

## Abstract (web summary)

Tracing the origins of variation at adaptive loci in British bank voles reveals that most variants exist as widespread polymorphisms shared with Europe and maintained during colonization, with lineage-specific differences in climate associations.

## Introduction

A species’ phylogeographic history—its past population size and movements—is a primary driver of the spatial distribution of genetic diversity^1–4^. For example, land bridges and ice sheets have structured genetic diversity by shaping colonization of new habitats and mediating the extent of gene flow among populations^3^. Glacial advances have restricted dispersal and displaced populations, reducing allelic diversity and fostering divergence among lineages^2,5,6^. In contrast, the retreat of ice sheets and the formation of land bridges have connected once-isolated environments, promoting long-distance dispersal and introgression, thereby altering allelic diversity, gene flow, and the overall population genetic structure^7^. However, while biogeographic processes are well established as a major force shaping the geographic distribution of neutral genetic diversity, their influence on adaptive genetic variation remains far less clear.

Because of its role in shaping both standing diversity and geographic location, the biogeographic history of a population may also influence adaptive capacity^8,9^. For instance, populations that have experienced particularly diverse climatic conditions over time may harbor greater standing genetic variation at functional genes, which can be advantageous when environmental conditions shift^10^. Responding quickly to rapid environmental change, including climate change, relies on harnessing this standing genetic variation, allowing selection on alleles with higher frequencies and greater adaptive value^11^. This dynamic interplay between historical events and genetic diversity is particularly likely in scenarios where populations face distinct climatic pressures, with the historical geographical range shaping past climate regimes and influencing future climatic responses^7^. Therefore, a population’s history could constrain or enhance local adaptation and may predict adaptive potential under future environmental conditions^12–14^. Yet, our ability to assess these effects has been limited: many species with well-known biogeographic histories lack genomic resources, while species with high-quality genomic data often lack a detailed phylogeographic history or information on the geographic distribution of adaptive variants.

**Fig. 1:**
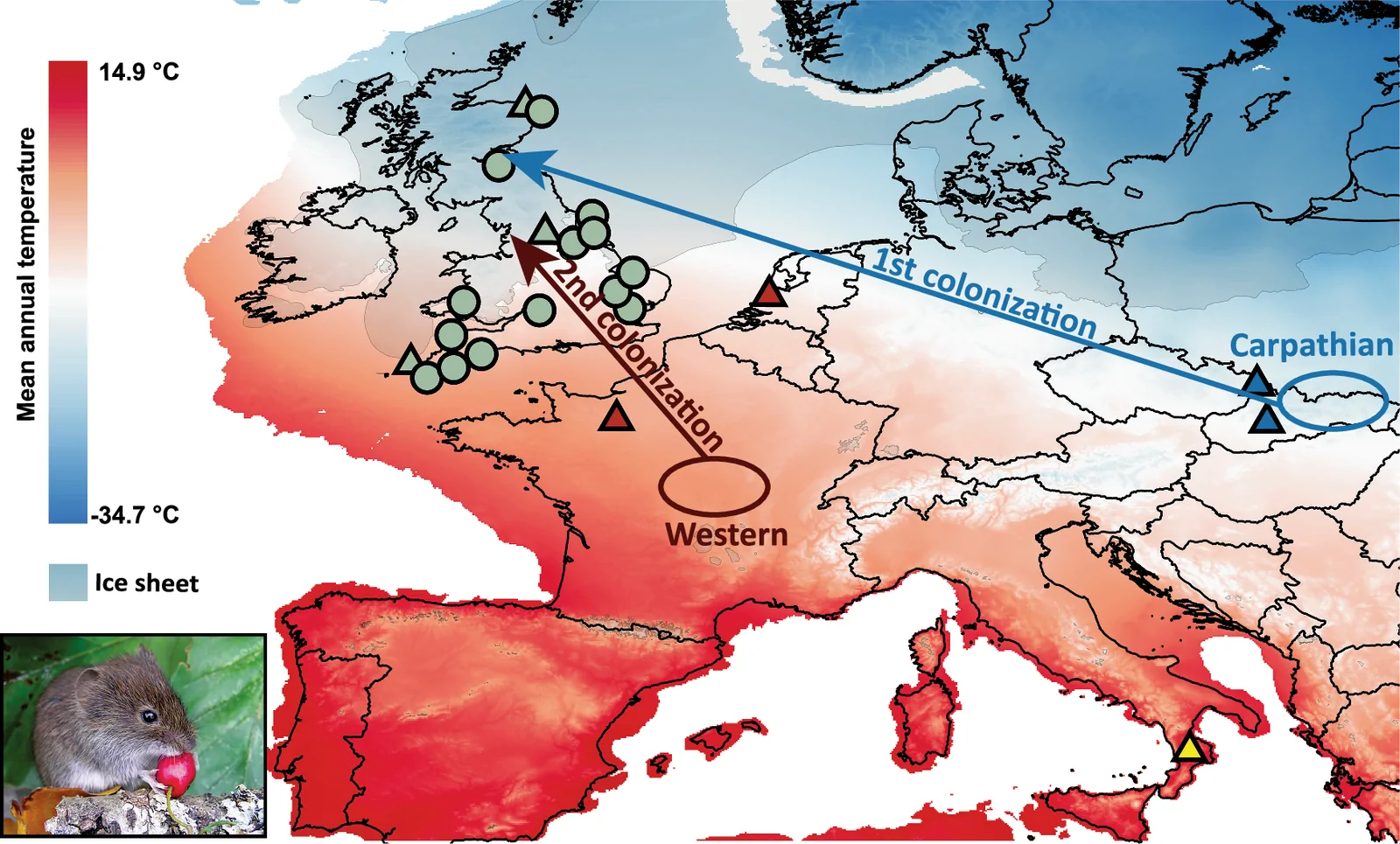
Population and environmental context. Population locations of the study species, the bank vole (inset image by P. Kotlík), relative to the Doggerland connection, the gradient of annual mean temperatures during the Last Glacial Maximum^28^, and the extent of the ice sheet at approximately 18,000 YBP. Triangles: populations analyzed for allelic origin and age from Britain (pale green), continental populations used as references for the Carpathian (blue) and Western (red) refugia, and a Calabrian population in southern Italy (yellow) as an outgroup^29^. These continental populations were selected as the most representative un-admixed populations from the respective refugia^15^. As such, geographic positions of the populations do not necessarily correspond to the locations of the glacial refugia. Filled circles in Britain represent population samples included in analyses evaluating the relationships between allelic origin and climate. Doggerland connected Britain and the European mainland until ~8200 YBP.

With a well-characterized biogeographic history^15–17^ and extensive genomic resources, including candidate climate-linked adaptive loci^18,19^, the bank vole (*Clethrionomys glareolus*) is an excellent model system for testing how population history shapes adaptive genetic variation^14^. The species spans habitats across Europe and Asia^16^, from coniferous forests and shrublands to bogs, hedgerows, and forest–steppe transitional zones, and diverse historical climates^14,20^. Bank voles colonized Britain from mainland Europe via Doggerland (Fig. 1), the land bridge that linked mainland Europe to the British peninsula^21^, after the Last Glacial Maximum (LGM) in two waves^5,17,18^: first from the Carpathian refugium during the Bøling–Allerød (14,700–12,890 YBP), and later from the Western refugium (presumed to be located in the Dordogne region in France^22,23^) between the Younger Dryas (12,800–11,700 YBP) and Doggerland’s submersion^24^ (~8200 YBP). These refugia experienced contrasting climates—warmer and drier in the Western refugium, colder and wetter in the Carpathians^25,26^—providing clear expectations about past climatic selection pressures and enabling comparisons between ancestral (mainland European) and more recently derived (British) alleles.

The different histories of these lineages, coupled with subsequent admixture^18,19^, make bank voles a powerful system for evaluating the role of population history in local adaptation. For example, strong evidence suggests that, following admixture, a hemoglobin gene variant originating in the Western refugium spread through populations of the Carpathian lineage in central Britain and largely replaced the resident hemoglobin allele^17^. This allele enhances cellular resistance to oxidative stress, potentially providing a selective advantage in the warmer southern habitats^18,27^, such as those in the Western refugium^28^. More broadly, we hypothesize that bank voles with Western ancestry may harbor other adaptations to warmer conditions, while Carpathian ancestry should be linked to colder climates. This difference may explain the dominance of Western genomic ancestry in southern Britain and the persistence of Carpathian ancestry in the north. Thus, past selection histories may continue to shape contemporary admixture and influence future adaptive potential.

To assess the impact of population history on the distribution of adaptive genetic diversity, we analyzed the origins and climate associations of genome-wide SNPs from British voles, focusing on loci previously implicated in climate adaptation^18^, and contrasting these results with a neutral reference set of alleles sampled from across the genome. For our analysis, we combined our existing British dataset^18^ with newly assembled whole genome data from four mainland populations representing the two colonization waves to Britain and one distant outgroup^29^ (Fig. 1; Supplementary Table 1). Our aims were to: (1) test if refugial origins of alleles at climate-linked SNPs differ from neutral expectations, (2) assess the relative contributions of the two refugia, and (3) evaluate the relationship between allele origin and present-day climatic conditions. We hypothesize that most adaptive alleles originated in mainland Europe rather than arising de novo in Britain. Based on past climates and evidence of population replacement, we further hypothesize a greater contribution from the Western refugium to adaptive variation, particularly for alleles conferring an advantage under warmer or drier conditions. Overall, our study highlights the central role of population history in shaping both current patterns of adaptive variation and species’ capacity to respond to future climate change. More broadly, understanding how population history provisions adaptive variation across the landscape will help extend insights from past demographic processes to predictions of future responses under climate change.

## Results

### Determining the origins of alleles

**Fig. 2:**
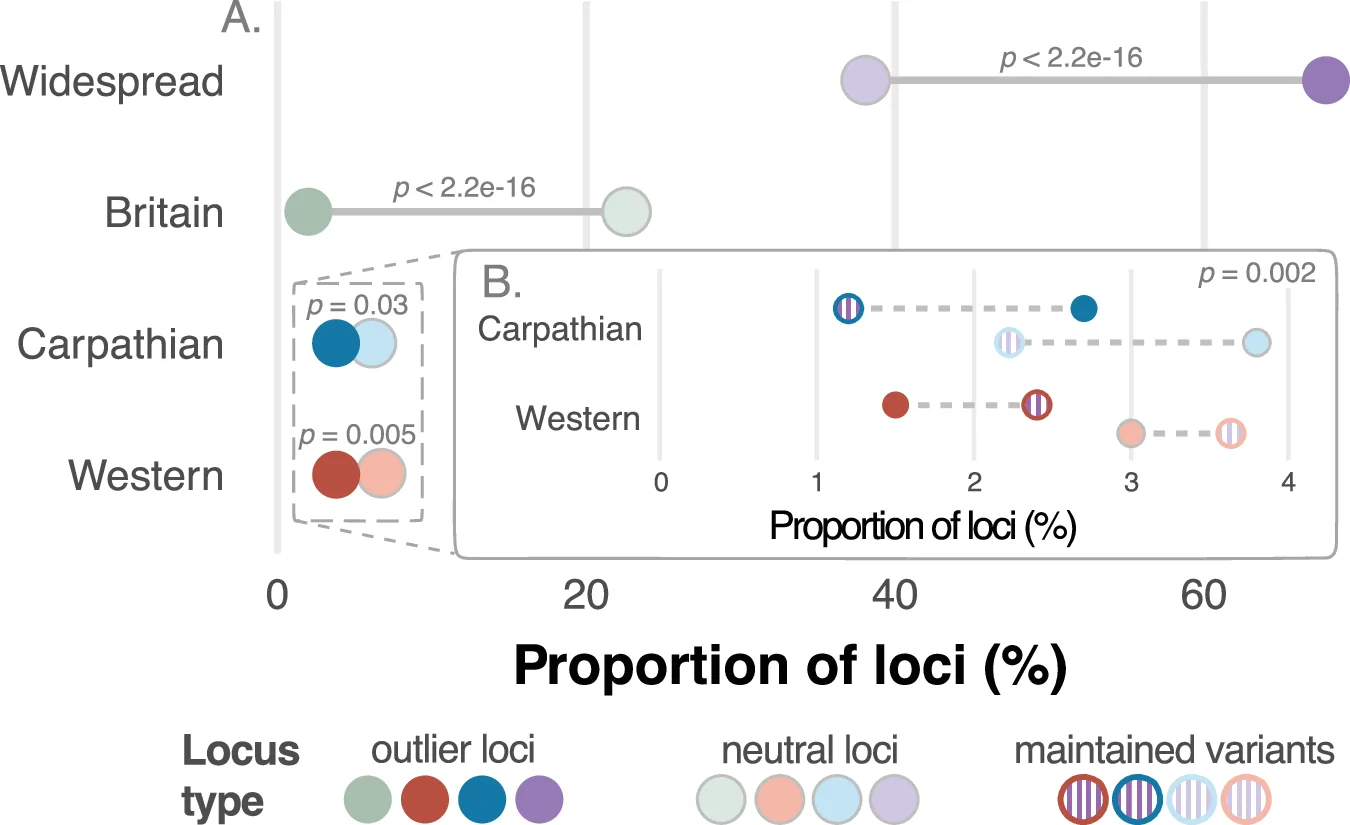
Proportion of variable loci in Britain relative to the inferred origin of variation at that locus. **A** Shown are the differences in the proportions of neutral and outlier loci where at least one allele is inferred to have a British, Carpathian, or Western specific origins based on haplotype trees (see Supplementary Table 2 for similar allele-frequency results). Loci were classified as “Widespread” if both alleles were present in both mainland refugia, and haplotype trees did not indicate a clear pattern of ancestry. *P* values comparing the number of neutral and outlier loci from a given refugia are based on chi-squared tests (*n* = 1624). **B** Differences in the proportion of loci with unique variation (solid circles) compared to those which represent widespread variation, where at least one allele was inferred as being maintained in and uniquely brought to Britain from the refugium shown (open circles with vertical stripes), colored by locus type and origin. Inset *P* value confirms a difference in the types of variants brought by each lineage, with more unique variation arriving with the Carpathian lineage but more maintained variation associated with the Western lineages based on a chi-square test (*n* = 221).

To distinguish signals of selection from other geographic processes (e.g., changes in effective population size or allele surfing^30^), we inferred the geographic origins of alleles at both outlier loci (previously identified candidates for climate adaptation^18^) and a corresponding set of neutral loci sampled from throughout the genome in populations. For each allele, origins were inferred using two complementary approaches: (1) allele frequencies compared across populations (i.e., presence or absence in refugial populations) and (2) phylogenetic ancestry for each allele inferred from haplotype trees constructed for each allele and the 100 kb region of linked SNPs. Both analyses reveal consistent general patterns (Fig. 2; Supplementary Table 2).

Most SNPs associated with climate adaptation occur as widespread polymorphisms present in mainland Europe as well as in Britain (Fig. 2; Supplementary Table 2), supporting the hypothesis that these variants originated on the continent rather than arising de novo post-colonization. Compared with neutral loci, outlier loci contained a much larger proportion of widespread polymorphisms (67.9 vs. 37.8%). Widespread alleles are present in both refugia, with the haplotype tree indicating that both lineages brought the variants to Britain. However, Western colonization was responsible for maintaining a greater proportion of variation at otherwise widespread alleles (i.e., 2.3% of widespread outlier variants appear to have only been maintained within the Western lineage compared to 1.2% in the Carpathian). When a single geographic origin of an allele could be assigned, unique variants classified as arising in the Carpathian refugium (outlier loci: 2.7%; neutral loci: 3.8%) were slightly more common than those assigned to the Western refugium (outlier loci: 1.6%; neutral loci: 3.1%). Both analyses also identified alleles unique to Britain; however, while these comprise a small fraction of the outlier loci the are a substantially higher percentage of the neutral dataset (2% outlier vs. 22.6% neutral).

### Estimating allele ages and divergence between haplotypes

**Fig. 3:**
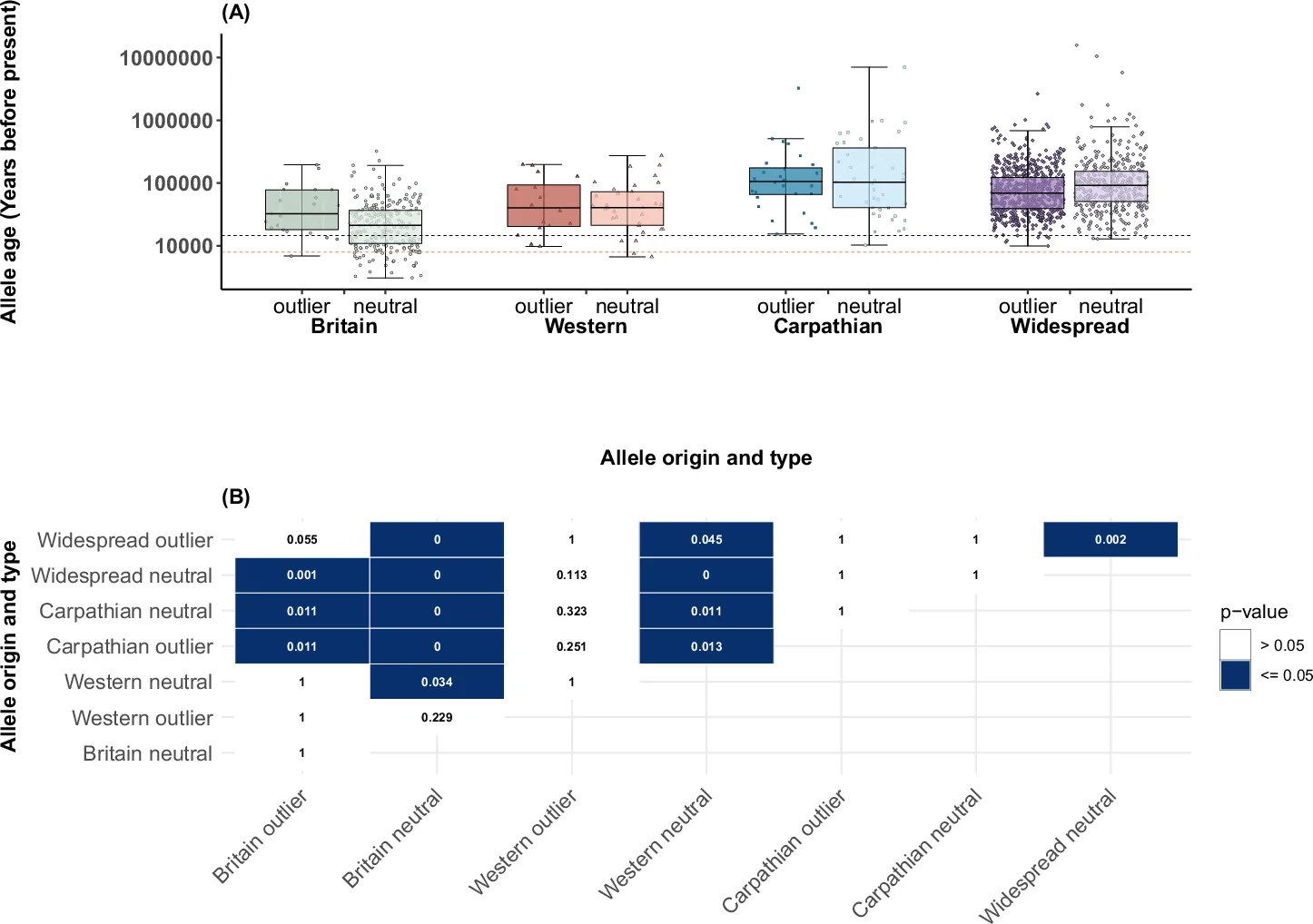
Ages of alleles based on assigned geographic origins. **A** The ages of alleles (points) grouped by inferred geographic origin, with overall distribution shown as box-and-whisker plots. Ages were estimated by converting coalescent time in generations to years before present (YBP), assuming a generation time of 0.5 years. The dark dashed line marks the period of Britain’s first colonization by bank voles^17^, beginning ~14,700 YBP, and the lighter dashed line marks the submersion of the Doggerland connection to mainland Europe. **B** Heatmap of *p* values from Kruskal–Wallis tests with *post hoc* Dunn’s test comparing allele ages (*n* = 1624).

To further validate the inferred geographic origins of alleles and gain insight into the processes shaping selective variation, we estimated allele ages at both outlier and neutral loci and calculated haplotype divergence within outlier loci. Converting coalescent times (Supplementary Fig. 1) into years using a generation time of 0.5 years revealed clear age differences among alleles of different geographic origins (Fig. 3). Widespread outlier alleles were significantly older (median = 69,146 YBP) than British outlier alleles (median = 32,664 YBP; Kruskal–Wallis, *p* = 2.56 × 10^−^^72^; Dunn test, *p* = 0.05) but not significantly older than Western outlier alleles (median = 40,515 YBP; Dunn test, *p* = 1). Furthermore, the Widespread outlier alleles were younger than the Carpathian outlier alleles (median = 106,319 YBP; Dunn test, *p* = 1). Neutral and outlier alleles from the same locality generally did not differ in age, except for widespread alleles: widespread outlier alleles (median = 69,146 YBP) were significantly younger than neutral widespread alleles (median = 91,718 YBP; Dunn test, *p* = 0.002). Most mainland-European variants were older than the submergence of Doggerland (~8200 YBP; 100% of outlier and 99% of neutral variants), with the vast majority of these predating the beginning of colonization of Britain (~14,700 YBP; 98%). Strikingly, this was also true for most alleles classified as “British” (86% of British outliers and 63% of the British neutral data), which were estimated to predate the arrival of the first colonists despite being classified as British in the other analyses.

We next compared divergence (*Dxy*) between haplotypes—defined here as the phased region of DNA surrounding an allele, including 50,000 bp upstream and downstream from the focal SNP—to further verify haplotypes inferred to contain an allele with a single refugial origin (i.e., Western or Carpathian; Supplementary Figs. 2–4) and further examine a scenario of polymorphism loss in widespread alleles during colonization. In addition to supporting unique refugial origins, the results indicated an additional 38 loci with widespread polymorphisms where one allele appears to have reached Britain from a single refugium (13 Carpathian; 25 Western; Fig. 2; Supplementary Table 2) and 4 loci with patterns consistent with differential allele loss in each lineage, with subsequent restoration of the polymorphism in Britain through admixture (Supplementary Fig. 5). Finally, Tajima’s *D* analyses further supported a scenario of population expansion without subsequent contraction (Supplementary Fig. 6), suggesting widespread retention of genetic variation across the genome.

### Correlation of allele frequencies with environmental variables

**Fig. 4:**
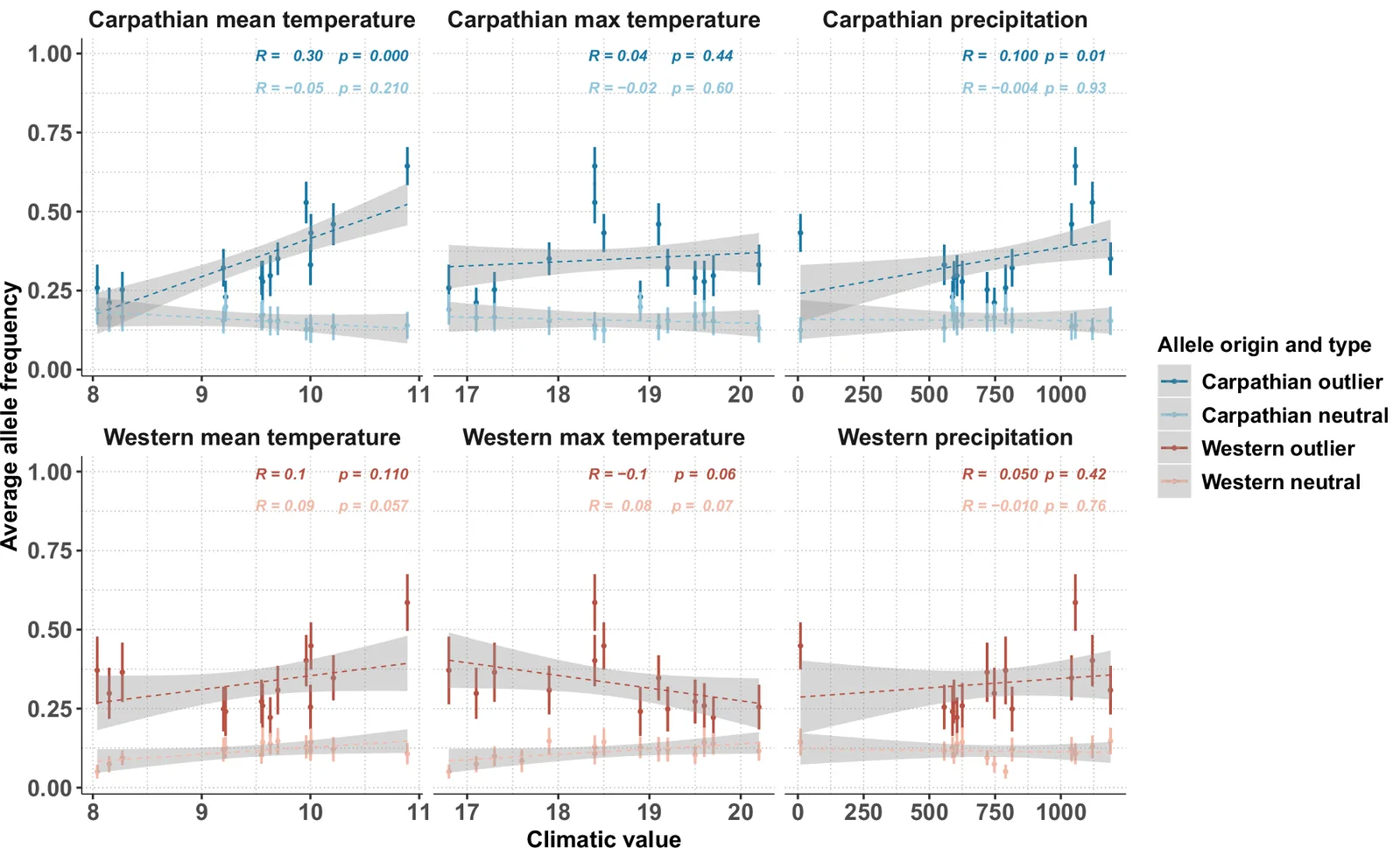
Allele frequencies of outlier and neutral alleles with Western or Carpathian refugial origins across Britain in relation to climatic variables. Regressions of population frequency of Carpathian outlier alleles against climatic variables were significantly positively associated with mean annual temperature and annual precipitation. In contrast, Western outlier alleles were not significantly associated with climate. Neutral allele frequencies reflected colonization history, including negative associations between Carpathian neutral alleles and mean annual temperature and positive associations between Western neutral alleles and maximum temperature of the warmest month. Carpathian and Western variants at outlier loci consistently occur at higher population frequencies compared to neutral alleles. Error bars represent standard error (*n* = 14).

**Fig. 5:**
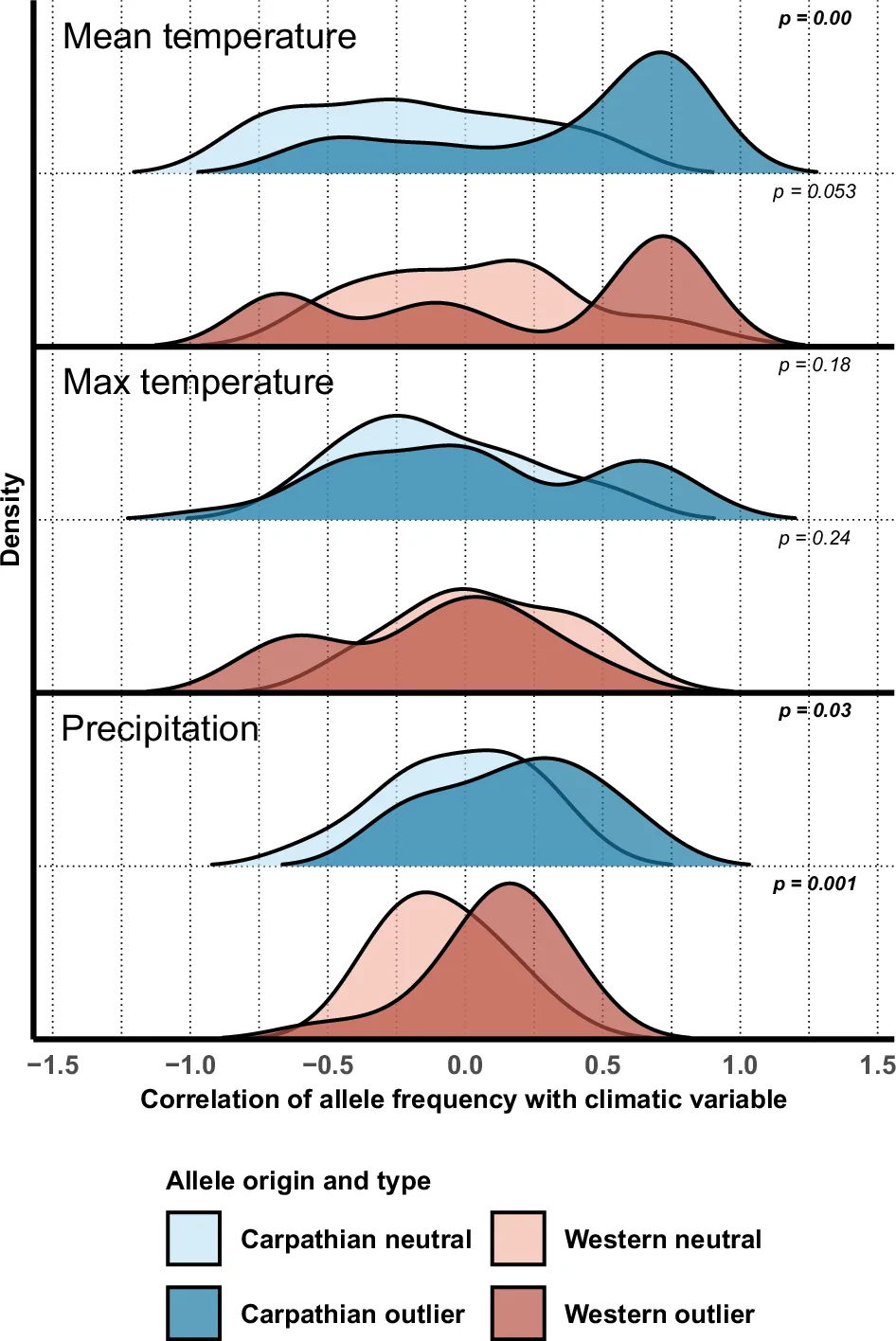
Distributions of correlation coefficients between climatic variables and population allele frequencies. In British bank voles, Carpathian outlier alleles showed stronger positive correlations with mean annual temperature than neutral Carpathian alleles, whereas distributions of both Carpathian and Western outlier alleles were more positively correlated with annual precipitation than neutral sets. Differences between the distributions of correlation coefficients for neutral and outlier alleles from each refugium were assessed using Kolmogorov–Smirnov tests.

To evaluate the influence of historical ancestry on allele-climate associations and test whether the Western alleles are more correlated with warmer conditions, we tested for correlations between climatic variables and the population frequency of alleles in Britain (Fig. 4). We then evaluated the overall distribution of correlation coefficients across loci (Fig. 5) and compared these with similar distributions for neutral loci. Overall, Carpathian outlier alleles showed significantly stronger positive correlations with mean annual temperature than the corresponding neutral Carpathian set (Figs. 4 and 5). Contrary to our expectation, Western outlier alleles did not exhibit stronger positive correlations with either mean annual or maximum temperature of the warmest month compared to the neutral Western alleles, although alleles at several individual outlier loci were either strongly positively or negatively associated (Fig. 5). The comparison with the neutral dataset highlights the distinct dynamics of outlier loci, particularly for temperature. For example, while Carpathian outlier alleles were significantly positively correlated with mean annual temperature, neutral Carpathian alleles showed the opposite trend (Fig. 4). By contrast, Western alleles—regardless of type—exhibited a weakly positive but nonsignificant relationship with mean annual temperature (Fig. 4). Despite differences in number of unique alleles contributed, the climate-associated outlier alleles (i.e., those presumably under selection) have risen in frequency regardless of source. Interestingly, Western outlier loci exhibit a weakly negative relationship to maximum temperature of the warmest month, contrasting with the positive relationship exhibited by the neutral loci (Fig. 4). None of the alleles when grouped by type and origin were significantly associated with isothermality (Supplementary Fig. 7), although Carpathian outliers were more positively associated with isothermality than Carpathian neutral alleles (Supplementary Fig. 8). Finally, Carpathian outlier alleles—and to a lesser extent Western outlier alleles—showed significant positive correlations with annual precipitation, whereas the corresponding neutral alleles did not (Figs. 4 and 5).

To control for geographic distance among populations, we conducted partial Mantel tests. These revealed significant positive correlations between allele frequencies and mean annual temperature for both Carpathian (*p* = 0.001, *r* = 0.60) and Western (*p* = 0.003, *r* = 0.48) alleles, as well as with maximum temperature of the warmest month (Carpathian: *p* = 0.001, *r* = 0.47; Western: *p* = 0.04, *r* = 0.25). We further applied partial redundancy analyses (pRDA)^31–33^ by locus origin, which account for population structure by incorporating neutral background divergence, to examine allele-climate relationships. The pRDA analyses indicate that many of the Carpathian and Western alleles are positively associated with mean annual temperature and precipitation, and a subset of Western alleles are negatively associated with maximum temperature of the warmest month, even after accounting for neutral genetic structure (Supplementary Figs. 9 and 10).

## Discussion

These results highlight how both shared ancestral variation and distinct refugial legacies shape the distribution and structure of adaptive genetic variation across the landscape in a temperate species of small mammal, the Eurasian bank vole. In Britain, most of the climate-associated SNPs in bank voles are ancient, widespread polymorphisms maintained across mainland Europe and during colonization from LGM refugia. While two continental European glacial refugia contributed unique alleles to British populations at candidate loci, the Carpathian refugium contributed slightly more than the Western refugium (Fig. 1, Supplementary Table 2). Climate-association analyses indicate that Carpathian-derived alleles are more strongly linked to warmer mean annual temperatures and higher precipitation in Britain. Together, these findings suggest that colonization processes and the genomic composition of past refugia strongly influence the availability and structure of adaptive genetic variation in British bank voles, with important implications for their capacity to respond to future climate change^18,34^.

Variation at climate-relevant adaptive loci in bank voles^18^ largely comprises widespread polymorphisms shared between Britain and the mainland refugia, which contributed to the island’s colonization. In this system, documented ancient admixture between bank vole lineages in mainland Europe^15^ likely explains the high frequency of widespread polymorphisms with deeper coalescent times in our dataset. Although neutral widespread alleles are common (34.8% of loci), adaptive loci harboring widespread alleles are significantly overrepresented relative to the neutral baseline in our system. Whether maintained by balancing selection^31^, or driven by parallel evolution in large populations^35–37^, widespread polymorphisms such as those seen in British bank voles are increasingly recognized as important for both local adaptation^35,38^ and adaptive divergence between species^39^. By serving as reservoirs of genetic variation across a broad geographic and environmental range, widespread polymorphisms may be particularly important for response to current and future environmental stressors^40,41^.

Previous analyses of clinal variation in British bank voles indicated that the genetic variation brought in by the second colonization (from the Western refugium) replaced many of the polymorphisms brought in by the first colonization (from the Carpathians) in the southern and central regions of Britain^17,19^. However, we also find that 1.7× more individual variants—both neutral and outlier—can be uniquely linked to the Carpathian refugium than to the Western refugium; that is, the percentage of loci with at least one allele exclusively from the Carpathian refugium is higher than those uniquely from the Western refugium. This may reflect the greater genetic diversity in the Carpathian population (*π* = 0.246, *θ* = 0.210) relative to the Western population (*π* = 0.218, *θ* = 0.207) or any other bank vole lineage in continental Europe^15^. Consequently, the Carpathian lineage may harbor a larger pool of standing genetic variation, making it a more likely source of unique adaptive variants^29,42,43^. However, because populations in the Western refugium originated from ancient admixture between Carpathian and Spanish lineages^15^, they may contain fewer private alleles unique to the Western lineage. These repeated admixture events during interglacial periods, many involving the Carpathian refugium as one of the source populations^15^, likely facilitated the spread of alleles between refugia, contributing to the widespread polymorphisms observed today. Our results indicate that variants present in Britain reflect this history of admixture, with the second (Western) colonization bringing in more variation from widespread outlier loci (i.e., alleles which were widespread in Europe but appear to be present in Britain due to the contributions of only one lineage). Together, these findings further reinforce the growing body of literature that points to the prominent contribution of the Carpathian refugium to shaping genetic diversity across all of Europe^3,15,44,45^.

Alleles at the vast majority of candidate adaptive loci in British bank voles exist as standing genetic variation that originated on the mainland and was retained during colonization in both lineages. Range expansions and the colonization of isolated areas are often associated with bottlenecks and founder events, driving allele loss and reducing genetic diversity at the edge of expansion^30,46^. However, subsequent admixture between lineages has the potential to recover this lost variation^47^, potentially facilitating rapid adaptation^43^. Our results suggest that a scenario of variability loss during colonization with subsequent geneflow recovering the polymorphism is uncommon in British bank voles, as variants from 5.5% of widespread loci (42/763) show evidence being maintained in only a single lineage during colonization, of which 4 loci have ancestry patterns consistent with each allele being carried by a different set of colonists (see Supplementary Fig. 5). While admixture between lineages in Britain has undoubtedly contributed to the spread of adaptive variation^19^, our data suggest both colonizing populations retained a considerable portion of the standing genetic variation at climate-associated SNPs. This retention likely contributed to the widespread success and regional adaptation of bank voles in Britain.

While our results strongly reinforce the importance of standing genetic variation accumulated over hundreds of thousands of years in British bank voles, they also shed light on the contributions of “new” mutations—those arising post-colonization—to adaptation. Although 22.6% of neutral loci harbor SNPs assigned as British in our analyses, only 2% carry tentatively adaptive SNPs inferred to have originated in Britain (22 loci). Most alleles at both candidate adaptive loci and neutral loci inferred as British predate 14,700 YBP, the earliest estimated colonization of the island^5,6,17^, suggesting they originated in mainland Europe but were unsampled or lost post-colonization. While this discrepancy between allele classification (i.e., new versus pre-existing in Europe) may partly reflect challenges in estimating recombination rate across the genome, which can skew the results towards older coalescent times^48,49^, our findings strongly imply that adaptation in bank voles in Britain is not primarily driven by new mutations. Post-colonization mutations account for only a minority of variation at loci carrying tentatively adaptive SNPs. This aligns with theoretical expectations^46^, and underscores that future adaptation of bank voles—and likely most other vertebrates—depends on the structure and distribution of long-standing variants^18,34,50,51^.

Finally, our results provide insights into the dual roles of current selection pressures and historical conditions in shaping the characteristics of adaptive variants among populations of British bank voles. Alleles from both refugia show positive associations with precipitation and temperature, reflecting ongoing selective pressures since colonization. However, across all three tests of climate association, candidate adaptive alleles originating in the Carpathian refugium are more strongly positively correlated with mean annual temperature than either neutral Carpathian alleles (which tend to be negatively correlated, as expected by phylogeographic history) or Western alleles, contrary to our hypothesis. This may reflect the selective advantage of Carpathian alleles at higher temperatures, whereas widespread alleles at the same loci confer advantages at cooler temperatures. Alternatively, warm-adapted Carpathian alleles may have actually originated in older, more southern refugia^15^ and been retained through the Carpathian refugium, consistent with their older ages. Although Carpathian outlier loci are strongly associated with mean temperature, they are not strongly associated with maximum temperature (Figs. 4 and 5). This may support the idea that thermal maxima, and possibly also minima, have harder physiological limits than other aspects of temperature response. In contrast, the muted temperature signal in Western alleles may reflect their initial distribution across latitudinal gradients, positive selection across a range of climates, or simply a smaller pool of important adaptive variants—although some, such as hemoglobin, have a notable impact^17,27^. Together, these results indicate that past population history has strongly influenced geographic patterns of adaptation to current climatic conditions.

Taken together, these findings on British bank voles reinforce the growing evidence in various species that population history strongly shapes the geographic distribution of adaptive genetic variation^12,13,51,52^ and emphasize an underused approach to advance our understanding of how population history influences adaptation to climate. Linking population history and the distribution of neutral variation^1–4^ to the distribution of adaptive variants^12,13,43,49^ has an important potential to improve predictions of regional responses to climate change^27^. Our results indicate that lineage history influences the characteristics and distribution of candidate adaptive SNPs, affecting local and future adaptation^18^. The extent to which these patterns occur in other organisms with similar phylogeographic histories—such as common shrews (*Sorex araneus*), water voles (*Arvicola terrestris*), field voles (*Microtus agrestis*), or pygmy shrews (*S. minutus*)^5^—is currently unknown, but these systems may provide frameworks for assessing how life-history traits or refugial locations mediate historical effects on adaptation. Given the rapid pace of environmental change, identifying the major factors shaping adaptive capacity^53,54^, including past population history, is critical for predicting species responses and informing conservation.

## Methods

### Sample selection, whole-genome sequencing, and locus selection

To investigate the role of allelic origin in shaping patterns of local adaptation, we extended an existing dataset of 111 whole genomes from 14 British bank vole populations^18^ by adding 41 additional genomes from five continental European localities, representing two LGM refugia and one outgroup (Fig. 1; Supplementary Table 1)^25,42^. Continental populations were chosen for their minimal secondary admixture, particularly in the Western refugium. Sila Grande, a population representing the most divergent bank vole lineage^29^ was used to root the tree as whole genomes from a close congener—the northern red-backed vole (*Clethrionomys rutilus*)—were unavailable. Furthermore, since the common ancestor of bank voles and northern red-backed voles diverged ~1.5 million years ago^55^, *C. rutilus* may be too divergent to accurately infer ancestral alleles within bank voles.

Genomic DNA was extracted from previously collected tissue samples (liver, spleen, and toes, see Marková et al.^47^) using the DNeasy kit (Qiagen, Valencia, CA), and whole-genome libraries were prepared with the NEBNext® Ultra™ II FS DNA Library Prep Kit (New England Biolabs, Woburn, MA). Extracted DNA was enzymatically fragmented, ligated to NEBNext adapters for Illumina sequencing, amplified by 3–5 PCR cycles using index primers (NEBNext Multiplex Oligos), and purified with NEBNext Sample Purification Beads. Fragment size and adapter incorporation were verified using an Agilent TapeStation (Santa Clara, CA). Libraries were normalized and sequenced as 150-bp paired-end reads on an Illumina NovaSeq 6000 platform at the Oklahoma Medical Research Foundation DNA Sequencing Facility (University of Oklahoma, OK). Average sequencing depth was 13× per sample. Samples were demultiplexed and converted to FASTQ format with bcl2fastq v2.20.0.422. Quality control of raw reads was performed using FastQC v0.11.9. Reads were processed with bbduk to remove residual Illumina adapters, trim read tails with a mean Phred score <10 over a 13-bp sliding window (*k* = 13), and trim the leftmost 10 bases (trimq = 10). Reads mapping to the reference mitochondrial genome of the bank vole were also removed. Remaining reads were mapped to the chromosome-level reference genome using *bwa* mem. The samtools package was used to convert SAM to BAM files, calculate quality statistics, remove unmapped and duplicate reads, tag heterozygous sites, index BAM files, and merge individuals into a single file.

SNPs were called and filtered using BCFtools v.1.9^56^ and VCFtools v.0.1.16^57^. SNPs were called using bcftools v1.9. Genotype calls for all genomic positions were generated with bcftools mpileup, applying a minimum mapping quality of 30 (-q 30), minimum base quality of 20 (-Q 20). SNPs were then called with bcftools call. A custom bash script was used with bcftools view to retain only the 1074 loci previously identified as under selection (see below), which were used as input for downstream analysis. Additional filtering with vcftools removed sites not meeting the following criteria: minimum mean depth ≥4 (--min-meanDP 4), maximum mean depth ≤60 (--max-meanDP 60), minimum allele count ≥2 (--mac 2), maximum number of alleles = 2 (--max-alleles 2), and ≤30% missing data (--max-missing 0.30). For consistency, both the newly generated and previously published genomic datasets were processed with the same pipeline.

We focused on climate-associated SNPs identified by Marková et al.^18^, who used two genome scan methods and a genotype–environment association approach to identify 1075 high-confidence candidate SNPs linked to climate adaptation in Britain. The procedure to identify outlier loci can result in a high rate of false positives due to factors such as fluctuations in effective population size (Ne), genetic drift at the range expansion margin, and allelic surfing mutation load. These factors influence the efficacy of selection and generate allele frequency patterns that mimic those expected from adaptive processes^58–62^. To minimize these false positives during outlier locus identification, Marková et al.^18^ used multiple approaches—accounting for population structure, population differentiation, and genotype–environment associations—and applied a stringent false discovery rate (FDR < 0.1). We analyzed 1074 of these loci across all 151 bank vole samples, as the hemoglobin beta subunit was excluded due to paralogous complexity, which complicates phylogenetic analysis^27^. To provide a set of null expectations based on Ne and population structure alone against which to compare outlier results, we selected an additional set of 1074 putatively neutral loci, randomly sampled across the genome, polymorphic in Britain, and located ≥50 kb away from the candidate loci^18^.

### Allelic origin inference from allele frequencies

We first inferred allelic origins using population-specific allele frequencies. Frequencies were calculated in PLINK v.2.0^63^ at three sites in Britain (representing each colonist lineage and one admixed population), four continental European sites (extant samples from populations representative of each refugium), and one individual from Calabria, Italy (Supplementary Table 1), representing the most divergent lineage of bank voles^29^. This ensured direct comparability with the haplotype tree analyses.

To infer potential origins, we used the following rules to assign classification:

1. British origin: allele present exclusively in Britain.
2. Refugium-specific: present in Britain and a single mainland refugium but absent in Calabria.
3. Widespread: present in Britain, plus both mainland refugia and Calabria.

Because all candidate SNPs were originally identified in Britain, each locus is polymorphic in Britain and consists of one widespread allele plus one variant allele, which may be widespread or assigned to a specific origin. For consistency, we report only the origin or climate associations of the variant allele by locus.

### Inference of geographic origin from a haplotype tree

Because allele frequency patterns can be misleading due to loss or fixation, particularly at adaptive loci, we also inferred allelic origins (H1 and H2) from haplotype trees. For each SNP, we constructed trees from a ~ 100 kb window (±50 kb) surrounding the focal site (100,001 bp total), corresponding to the average linkage block size of ~100 kb in the genome, as determined by linkage disequilibrium analysis. These ~100 kb blocks were phased in BEAGLE v5.4^64^ to obtain haplotypes relative to reference and alternative alleles at the focal loci. The phased haplotype blocks were stored in VCF format and then converted to FASTA files using the vcf2fasta utility from the vcflib v1.0.9 C++ library^65^. The FASTA files were used in the tree inference conducted by RAxML v.8.2.4^66^ under the GTRCAT model with 30 independent runs. This analysis produced 1074 trees in Newick format, each rooted using one bank vole sample from Sila Grande, Italy, from the Calabrian refugium^29^. Newick trees were visualized and edited in *R* v4.1.3^67^ using the ggtree v3.5.3 package^68^ to facilitate the detection of patterns of allelic ancestry.

Allelic origins were assigned as follows:

1. British: allele identified as British in the frequency analysis formed a monophyletic British clade.
2. Refugium-specific: haplotype carrying the allele in Britain coalesced with that allele from a single mainland refugium.
3. Widespread: widespread from the allele frequency analysis; no clear pattern of ancestry between Britain and mainland alleles.
4. Unclear: allele found in Britain and only one refugium, but lacking monophyly with refugial alleles.

Differences in classification between outlier and neutral loci were evaluated with chi-square tests.

### Validation of allelic origin using coalescent and divergence analyses

We further validated inferences about allelic origin by estimating allele ages and pairwise divergence (*Dxy*)^69^. Coalescent ages were estimated using RUNTC v.0.0.1^48^ for both neutral and outlier datasets, assuming a per-base, per-generation recombination rate (*r*) of 6 × 10^−9^ (calculated as *r* = *ρ*/4*Ne*, with *Ne* = 386,000 and *ρ* = 0.0091 from *Mus musculus*^70^). Coalescent times (in generations) were converted to years before present (YBP), assuming a generation time of 0.5 years, based on breeding seasonality (March–October; males breed from ~2 months of age, females from ~2 to 5 months)^16^. We expected mainland alleles to predate the submersion of Doggerland (8200 YBP)^71,72^, whereas alleles of British origin would postdate the initiation of colonization (~14,700 YBP). Differences in allele ages across origins (British, Western refugium, Carpathian refugium) and locus type (outlier vs. neutral) were tested with the Kruskal–Wallis test followed by *post hoc* pairwise comparisons. We hypothesized that alleles unique to Britain originated after colonization and would therefore be significantly younger than alleles of a single refugial origin, and widespread alleles would generally be the oldest variants.

To complement coalescent age estimates, we calculated pairwise divergence (*Dxy*) between haplotypes containing putatively adaptive SNPs using PIXY v.1.2.7^69^ under default parameters. We expected alleles restricted to a single refugium to exhibit lower *Dxy* than widespread alleles at the same locus, because widespread alleles—having persisted longer—should have accumulated greater divergence. We performed *Dxy* analyses on a locus-by-locus basis, using two distinct sets of VCF files generated by in-house bash scripts, running *Dxy* analyses for haplotypes carrying the alternative allele separately from those with the reference allele. This allowed us to estimate *Dxy* separately for haplotypes associated with each allele at a candidate locus.

*Dxy* was also used to test whether genetic diversity at widespread loci was maintained during colonization. Specifically, we assumed alleles that were widespread with respect to allele frequency data but refugium-specific with respect to haplotype trees (i.e., variants that may have been maintained during colonization) would have lower *Dxy* to their inferred-refugium compared to the other refugium, as would be expected if the allele was only maintained in one lineage during colonization. If both colonizing lineages carried and maintained the same alleles, *Dxy* values for Western–British and Carpathian–British comparisons should be similar. Conversely, if each lineage lost a different allele during colonization but the polymorphisms were later regained in Britain during secondary contact, *Dxy* values for Western–British comparisons would differ significantly from Carpathian–British comparisons for both reference and alternative alleles. These differences were evaluated using two-sample t-tests in *R* v.4.1.3^67^ (function *t.test*).

Finally, to assess potential demographic bottlenecks during colonization, Tajima’s *D* was calculated from the neutral dataset using VCFtools v.0.1.16.

### Correlation of allele frequencies with climatic variables

To test the hypothesis that lineage-specific environments influenced allelic characteristics (e.g., whether a greater proportion of warm-associated alleles originated from the Western refugium; H3), we analyzed the relationships between climatic conditions and allele frequencies from 110 bank voles representing 14 British populations (including the three used for inference of allelic origin) for both outlier and neutral datasets. Allele frequencies were estimated in PLINK v.2.0^63^, and correlations between population-level allele frequencies and climatic variables were calculated in *R* v.4.1.3^67^ using the function *cor.test*. Only alleles with an inferred geographic origin were included. In total, 29 and 17 outlier loci carried at least one allele of Carpathian and Western origin, respectively; for neutral loci, there were 41 Carpathian and 33 Western.

Climatic variables were selected based on those previously shown to shape the adaptive landscape^18^ at each sampling site: mean annual temperature (BIO1), isothermality (BIO3), maximum temperature of the warmest month (BIO5), and annual precipitation (BIO12), extracted from WorldClim^27^. Alleles were grouped by their inferred origin (Carpathian or Western), as determined by both haplotype tree and allele frequency analyses, to test whether Western-origin alleles showed stronger associations with warmer conditions than Carpathian-origin alleles. To compare outlier versus neutral expectations, we examined the distributions of correlation coefficients for each refugial group using Kolmogorov–Smirnov tests. We further regressed the overall population frequencies of a set of alleles by refugial group against climatic variables. These approaches allowed us to assess whether outlier loci showed significantly stronger correlations with climate than neutral loci, consistent with adaptive signals. Because correlation analyses do not account for population divergence or spatial autocorrelation, we complemented them with (1) a partial Mantel test, conducted in *R* (mantel.partial, vegan v.2.6.4) to evaluate correlations between allele frequencies and mean annual temperature while controlling for geographic distance, and (2) a partial redundancy analysis (pRDA)^31–33^ to evaluate the relationship between allele frequencies and environmental variables while controlling for population structure. The pRDA analysis was implemented in vegan v2.6.4^73^. Four climatic predictors were included, and the analysis was conditioned on ancestry coefficients (*K* = 3) from Marková et al.^18^ which served as proxies for neutral genetic differentiation. Significance of the overall model and constrained axes was assessed using permutation-based ANOVA with the function anova.cca. Finally, correlations between each locus and each climatic predictor were calculated using a modified version of the script provided in Forester et al.^32^.

### Statistics and reproducibility

Our study used a SNP dataset of whole-genome sequences from 111 bank voles sampled across 14 localities, originally generated by Marková et al.^18^ and accessible via Marková et al.^74^ and at da Cruz et al.^75^. This dataset was extended with 44 additional whole-genome sequences from five continental European localities. To infer the geographic origin of 1074 outlier loci previously identified as correlated with environmental variables^18^ and a corresponding neutral dataset of 1074 loci, we calculated allele frequencies, constructed haplotype trees from phased haplotype blocks of 100,001 bp surrounding the locus, estimated allele ages, and calculated *Dxy*, as detailed in the Materials and Methods. After assigning geographic origins, we conducted subsequent analyses of climatic relationships in *R*, as detailed in the methods section. Maps were created in QGIS v.3.32.1 using temperature layers from WorldClim (https://www.worldclim.org/data/v1.4/paleo1.4.html#google_vignette). Graphs were generated with the ggplot2 package in R v.4.4.2, with final figure edits performed in Adobe Illustrator using the original graphical output.

### Reporting summary

Further information on research design is available in the Nature Portfolio Reporting Summary linked to this article.

## Supplementary information

Transparent Peer Review file

Supplementary information

Description of Additional Supplementary Files

Data Set 1

Reporting Summary

## Data Availability

The raw Illumina sequencing data generated in this study are deposited at NCBI in the Sequence Read Archive (SRA) under BioProject PRJNA1097528. The SNP datasets used in this study can be accessed through the Dryad Digital Repository at https://datadryad.org/dataset/doi:10.5061/dryad.k3j9kd5qq. Numerical source data for graphs and charts can be found in Supplementary Data 1.

## Code availability

The code used to conduct the analysis in this study can be accessed at: https://github.com/marcticruz/Origins-of-adaptive-genomic-variation-in-a-wild-rodent.
