# Supplementary material for: Origins of adaptive genomic variation in a wild rodent
Source: Commun Biol. 2026 Jun 26;9:1360. doi: 10.1038/s42003-026-10511-6 (PMC13677672; doi:10.1038/s42003-026-10511-6)

nature portfolio

Peer Review File

Origins of adaptive genomic variation in a wild rodent

Corresponding Author: Mr Marcos da Cruz

This file contains all reviewer reports in order by version, followed by all author rebuttals in order by version.

Version 0:

Reviewer comments:

Reviewer #1

(Remarks to the Author)
This study aims to reconstruct and understand the age and origin of refuge-specific adaptive alleles in recolonized regions of western Europe in a vole species. This is a potentially interesting study and leverages a relatively well understood system to ask a cutting-edge question important to understanding present-day patterns of postglacial genetic variation. The results are not particualry striking (although results of Fig 4 are interesting), but I don't think that detracts from the relevance of the paper. I do, however, have one major analytical concern that needs to be addressed, and one point on writing as well as some line-by-line comments and clarifications of supplementary methods.

(a) analysis - There is extensive literature (both theoretical and empirical) detailing (1) how changes in effective population size affect the efficacy of selection on alleles; (2) the strong effects of allelic surfing, mutation load, and drift at the edge of an expanding front (Excoffier and Ray 2008; Gilbert et al 2018); and (3) the interplay between fluctuating Ne and efficacy of selection and/or effects of fluctuating selection pressure (Johnson et al 2023 and references therein; Engen et al 2005; Myrhe et al 2016). All three of these topics are directly relevant to the study but are not sufficiently discussed. More importantly, they can dramatically alter results and lead to high false positives. While the authors are looking at a priori defined putatively adaptive loci, they cannot make a convincing case for their results without considering appropriate null hypotheses that accommodate these factors. The simplest way I can imagine doing this is picking a same number of random loci throughout the genome and performing the same set of analyses on them as a null sample. These effects may also contribute to why so many loci are categorized as 'widespread' in Fig 2, or compared to non-adaptive loci. (Although the fact that there were ~8-10 high amplitude glaciations over the last 1 Myrs means these alleles could also have mixed during prior interglacials and therefore appear widespread). Similarly, it could potentially be interesting to compare the candidate alleles' associations to a climatic variable thought not to be important (with the expectation there is not an association). Either way, I think some sort of comparison to a null sample needs to be considered.

(b) writing – the authors do a good job of setting up the structure of the paper with their hypotheses, however these are rarely revisited in the results section. It would help if in results sub-section it refers briefly back to whether it supports the original hypothesis.

Line-by-line notes:
1. L41 – and population size
2. L71 – there is no real way to know those were refugia without fossil data
3. L245-8 – this may be true but it is not convincing without comparing these distribution to the association of the non-refugial (i.e. widespread) alleles to the same climatic variables. In other words, it lacks necessary statistical context without adding the widespread loci to Figure 4.
4. L256-7 – Given that Fig S5 plots Dxy, I don't see how this is evidence that bottlenecking did not occur. It seems the authors really need to run SMC++ or skyline plots to look at population-specific change in Ne over time and/or spatial patterns of genetic variation to test for this (or at a minimum run Tajima's D on the non-candidate loci)
5. L277 – the authors may also consider if the refugia were stable over subsequent glaciations, then repeated admixture events may have sustained variation over time and manifest today as 'widespread' with deeper coalescence times.
6. L287 – again, having population-specific Ne estimates would help clarify this.
7. L404 – I believe in the SI it says 50kb on either side (100kb total?)

Fig 3 – I think it would be helpful to show the distribution for 'widespread' alleles on this chart as well for context.

Supplemental Methods–
- Bbduk version?
- Bwa mem flags (if any)
- The rho used to calculate recombination rate was from mouse, but where was the Ne taken from? The Ne can be calculated per population or per species, how would it be impacted by the change of Ne that come from populations merging and splitting during glaciations? If the age of allelic origin is important to the story (which it seems to be) then the estimation of r here is as well. If it was taken from mouse, that is not a reasonable choice.
- Where was the generation time of 0.5 years taken from?

Refs
Engen, S., Lande, R. and Saether, B.E., 2005. Effective size of a fluctuating age-structured population. Genetics, 170(2), pp.941-954.
Excoffier, L. and Ray, N. 2008. Surfing during population expansions promotes genetic revolutions and structuration. – Trends Ecol. Evol. 23: 347–351.
Gilbert, K. J., Peischl, S. and Excoffier, L. 2018. Mutation load dynamics during environmentally- driven range shifts. – PLoS Genet. 14: e1007450.
Johnson, O.L., Tobler, R., Schmidt, J.M. and Huber, C.D., 2023. Fluctuating selection and the determinants of genetic variation. Trends in Genetics, 39(6), pp.491-504.
Myhre, A.M., Engen, S. and Sæther, B.E., 2016. Effective size of density-dependent populations in fluctuating environments. Evolution, 70(11), pp.2431-2446.

Reviewer #2

(Remarks to the Author)
This study builds upon a recent study by many of the same authors (Markova et al. 2023) that seeks to investigate the origins of genomic variation linked to climate in present-day British bank voles. These studies are starting to build upon classic phylogeographic studies to look at how past standing variation might influence future adaptation in populations to ongoing change. The main limitation is likely the limited geographic scope and the focus on Britain (which was the focus of the previous paper also). Should there be concerns in regions that are more likely to be impacted by climate in the more southerly regions for example and the implications for more northerly populations in Scandinavia. I certainly see the value of this study however and how it has implications well beyond the current study system.

The keywords refer to the genus as Myodes but the main ms to Clethrionomys. Now, this may be deliberate given the back and forth with the classification of this species in recent years so just check if you meant this or if a mistake.

It is somewhat confusing as to why samples representing the Western lineage have been chosen that are quite distant from the proposed location of the actual refugium. This is an incredibly well-studied phylogeographic system (probably the best-studied in Europe at least in terms of a mammal) with a huge number of samples that should have been available. Was there a reason that samples could not have been selected closer to the proposed refugium in a similar way to the Carpathian refugium? If direct comparisons are being made, the sampling is not exactly uniform between both and it’s unclear why.

Lines 182-186. Would it not be possible that at least some of the alleles unique to Britain could also have been missed given the limited number of individuals screened from the mainland. This obvious possibility only hinted at and the sampling design/locations should be critiqued here rather than just stating that they may have been missed. This goes back to the point above that that is the main concern with the ms: the sampling is fairly limited from the two refugia representing the mainland. This definitely will impact some of the findings and inferences drawn here.

Reviewer #3

(Remarks to the Author)
Dear authors,

Thank you for submitting your manuscript on the origins of adaptive genomic variation in bank voles. While the topic is potentially very interesting, there are several areas that require thorough revision before it can be considered for publication.

One major improvement would be to provide a picture of the target animal species. Including information about their habitat, behavior, and ecological aspects would greatly assist readers in understanding the context and significance of your study. This additional information will enhance the overall clarity and impact of your findings.

Furthermore, I noticed that the figures included in this manuscript are poorly prepared and some of them could benefit from being compiled into a single figure. By consolidating these figures, you can present your data more effectively and improve readability for readers.

Additionally, it is important to address an issue regarding the presentation of your main results. Currently, they are hidden within supplementary files which significantly diminishes their importance. To ensure that your key findings receive proper attention, consider integrating them into the main body of the manuscript.

Moreover, I observed that some figures presenting statistical results lack legends indicating their significance. It is crucial to include clear explanations or captions alongside these figures so that readers can understand their implications without confusion.

Lastly, there are still areas throughout the manuscript where confusion persists and needs to be addressed. For example, in one instance it states in the legend "Carpathian alleles were significantly more negatively correlated with mean annual temperature than Western alleles with p-value = 0.01 according to Mann–Whitney U test," but upon reviewing Figure (b), I found that the correlation coefficient for 'Carpathian' was actually lower than that for 'Western'.

Version 1:

Reviewer comments:

Reviewer #1

(Remarks to the Author)
I was the previous reviewer who requested the test on background neutral loci, and I'm very glad the authors took that feedback seriously. The paper is now much stronger, and the results are compelling. It addresses several fundamental questions in population genetics that are typically only explored through simulations, so I believe this study will serve as a valuable — and likely highly cited — empirical example. The new figures are clear and well presented. I have only minor suggestions below; overall, I am now very happy with and fully supportive of this manuscript.

I noticed that the Carpathian outlier loci are strongly associated with mean temperature but not maximum temperature (Figs. 4–5). This seems reasonable, as critical thermal minima and maxima may have harder physiological limits than other aspects of temperature response. Just a thought as to why this pattern might be expected.

L59 – suggest removing the comma after 'resources'
L103 – consider slightly rephrasing aim 1. You're really testing whether refuge-derived allele-linked SNPs show a different spatial pattern than neutral ones, as would be an expected result of local adaptation/selection
L118 – add a citation for allele surfing
L222-4 – great!
L262-4 – while true, your results actually show something stronger: despite differences in magnitude of alleles contributed, the temp- and precipitation-associated alleles (presumably under selection) have risen in frequency regardless of source. This is expected, but it's nice to see a real example of it.
L321 – for clarity, consider replacing upward with "younger" or "older"
L323-5 – great
L384 – consider adding something about 'null expectations based on Ne and population structure alone against which…"
Fig 1 – I suggest nudging the labels for colonization and Carpathian so they don't overlap the black lines, which makes it harder to read
Fig 2 – great, really shows how strong the results are. This may be a conversion issue in the submission system, but the bold font has strange spacing—if not already I would suggest changing font to arial or Helvetica or something standard.
Fig 3 – I suggest adding the significance results (where positive) to the figure itself—I see what the authors are doing but it's strange to show a heatmap of p values since it's more of a threshold rather than a gradient type of result. Or, just color the ones that are significant and leave the rest white etc.
Fig 4- great figure

Reviewer #2

(Remarks to the Author)
Having reviewed the previous version of the ms, I am satisfied with how the authors have responded to my comments and concerns. I also am in agreement with the responses to the other reviewers and have no further comments to make.

I look forward to seeing this published hopefully.

Reviewer #3

(Remarks to the Author)
Dear editor,
Authors have thoroughly addressed all my comments, and I believe this version is now suitable for publication.

Open Access This Peer Review File is licensed under a Creative Commons Attribution 4.0 International License, which permits use, sharing, adaptation, distribution and reproduction in any medium or format, as long as you give appropriate credit to the original author(s) and the source, provide a link to the Creative Commons license, and indicate if changes were made.
In cases where reviewers are anonymous, credit should be given to 'Anonymous Referee' and the source.
The images or other third party material in this Peer Review File are included in the article's Creative Commons license, unless indicated otherwise in a credit line to the material. If material is not included in the article's Creative Commons license and your intended use is not permitted by statutory regulation or exceeds the permitted use, you will need to obtain permission directly from the copyright holder.
To view a copy of this license, visit https://creativecommons.org/licenses/by/4.0/

Dear Mr. da Cruz,

Your manuscript entitled "Origins of adaptive genomic variation in a wild rodent" has now been seen by 3 referees, whose comments are appended below. You will see from their comments copied below that while they find your work of potential interest, they have raised quite substantial concerns that must be addressed. In light of these comments, we cannot accept the manuscript for publication, but would be interested in considering a revised version that addresses these serious concerns.

We hope you will find the referees' comments useful as you decide how to proceed. Should further experimental data or analysis allow you to address these criticisms, we would be happy to look at a substantially revised manuscript.

In particular, please note that the following revisions would be necessary for us to contact our referees again:

Reviewer 1 suggests additional analysis based on randomly selected regions of the genome to provide a null sample. Reviewer 2 seeks clarification on the choice of Western lineage samples, and reviewer 3 suggests combining and improving the figure presentation. The reviewers also identify a number of minor changes that should be addressed.

We are committed to providing a fair and constructive peer-review process. Do not hesitate to contact us if you wish to discuss the revision or if there are specific requests from the reviewers that you believe are technically impossible or unlikely to yield a meaningful outcome.

If you decide to submit a revised version, we ask that you ensure your manuscript complies with our editorial policies. Please see our revision checklist for guidance on formatting the manuscript and complying with our policies. A comprehensive guide to our formatting requirements for final submissions is also available for your reference here.

We hope to receive your revised manuscript within 3 months, but appreciate that every situation is unique. Please take as long as necessary to address these concerns in full, including performing any additional experimental work required. We look forward to receiving your revised manuscript when it is ready and will not enforce any specific deadline. However, please bear in mind that if the revision process takes significantly longer than six months, we may take into account whether anything similar has been accepted for publication at Communications Biology or published elsewhere in the meantime.

Please do not hesitate to contact me if you have any questions or would like to discuss the required revisions further. Thank you for the opportunity to review your work.

Best regards,

John Mulley, PhD
Editorial Board Member
Communications Biology
orcid.org/0000-0002-1537-7316

Referee expertise:

Referee #1: Genomics, speciation

Referee #2: Molecular ecology, rodent genomics

Referee #3: Taxonomy, rodent/mammalian evolution

Reviewers' comments:

**Reviewer 1 comments**	**Response**
1. This study aims to reconstruct and understand the age and origin of refuge-specific adaptive alleles in recolonized regions of western Europe in a vole species. This is a potentially interesting study and leverages a relatively well understood system to ask a cutting-edge question important to understanding present-day patterns of postglacial genetic variation. The results are not particularly striking (although results of Fig 4 are interesting), but I don't think that detracts from the relevance of the paper.	Thank you for acknowledging the value of using a well-characterized system to investigate an important evolutionary question.
1.b I do, however, have one major analytical concern that needs to be addressed, and one point on writing as well as some line-by-line comments and clarifications of supplementary methods.<br>(a) analysis - There is extensive literature (both theoretical and empirical) detailing (1) how changes in effective population size affect the efficacy of selection on alleles; (2) the strong effects of allelic surfing, mutation load, and drift at the edge of an expanding front (Excoffier and Ray 2008; Gilbert et al 2018); and (3) the interplay between fluctuating Ne and efficacy of selection and/or effects of fluctuating selection pressure	We agree these are important factors and although our goal is to evaluate origins of previously identified loci, we have followed your suggestion and analyzed a null panel of alleles which represent random loci sampled from throughout the genome. The results of these analyses are integrated throughout the paper, including as updates to Figures 2-5, in the Results and Discussion, and in the supplement. We believe these additions substantially strengthen the manuscript. For example, although adaptive and null allele sets show a similar pattern with respect to allele origin, widespread polymorphisms are disproportionately more common and new

(Johnson et al 2023 and references therein; Engen et al 2005; Myrhe et al 2016). All three of these topics are directly relevant to the study but are not sufficiently discussed. More importantly, they can dramatically alter results and lead to high false positives. While the authors are looking at a priori defined putatively adaptive loci, they cannot make a convincing case for their results without considering appropriate null hypotheses that accommodate these factors. The simplest way I can imagine doing this is picking a same number of random loci throughout the genome and performing the same set of analyses on them as a null sample. These effects may also contribute to why so many loci are categorized as 'widespread' in Fig 2, or compared to non-adaptive loci. (Although the fact that there were ~8-10 high amplitude glaciations over the last 1 Myrs means these alleles could also have mixed during prior interglacials and therefore appear widespread). Similarly, it could potentially be interesting to compare the candidate alleles' associations to a climatic variable thought not to be important (with the expectation there is not an association). Either way, I think some sort of comparison to a null sample needs to be considered.

mutations are far less frequent among the putatively adaptive loci. Our interpretation of the relationship between allele frequency and temperature were also strengthened by the null comparison: while Carpathian neutral alleles are negatively associated with temperature, consistent with geographic distribution, the Carpathian adaptive alleles are significantly positively correlated (Fig. 4), strengthening the evidence for the association between adaptive alleles and warmer climates. Likewise, our interpretation of the correlation between population allele frequency and climate was strengthened by the comparison of null and candidate loci (Figure 5).

We agree that the factors that you mentioned are important, and result in a high rate of false positives during outlier locus identification, and we have been careful to address this. The set of outlier loci included in the current study were previously identified by Markova et al. (2023) as the intersection of loci identified through separate analysis of population structure, population differentiation, and genotype-environmental associations using a stringent threshold of false discovery rate (FDR < 0.1). We have emphasized this in the main body of the manuscript on lines 377–383: "*The procedure to identify outlier loci can result in a high rate of false positives due to factors such as fluctuations in effective population size (Ne), genetic drift at the range expansion margin and allelic surfing mutation load. These factors influence the efficacy of selection and generate allele frequency patterns that mimic those expected from adaptive processes* [55–59]*. To minimize these false positives during outlier locus identification, Markova et al.*[18] *used multiple approaches—accounting for population structure, population differentiation, and genotype-environmental associations—and applied a stringent false discovery rate (FDR < 0.1)*."

Finally, our additional analyses showing differences between outlier and neutral alleles in correlations with environmental variables

indicates that it is likely we managed to avoid an excessively high rate of false positives.

In response to the reviewer's suggestion, we performed new comparative analyses, which show that differences between outlier and neutral alleles in their correlations with environmental variables likely indicate that we have avoided an excessively high rate of false positives.

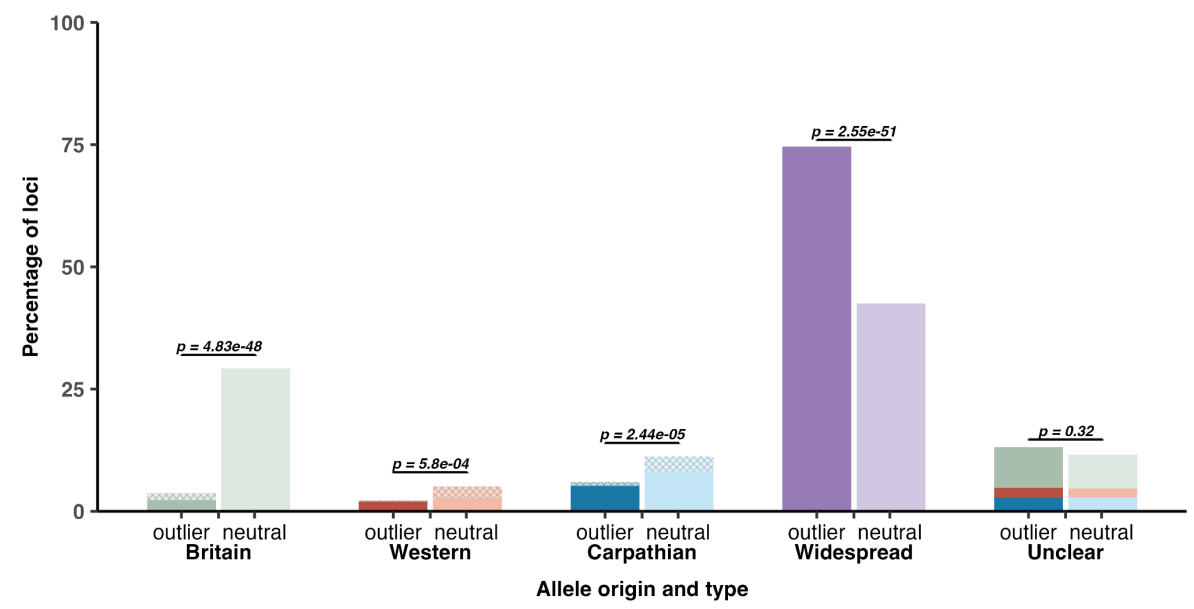

Figure 2: Percentage of loci with alleles in Britain that can be assigned a British, the Western, or Carpathian origin. Shown are the proportions of loci where at least one allele is inferred to have one of these specific origins based on haplotype trees (see Table S2 for similar allele-frequency results). Loci were classified as 'Widespread' variants if both alleles were present in both mainland refugia and haplotype trees did not indicate a clear pattern of ancestry. Loci were classified as 'Unclear' when allele-frequency analyses indicated a British, Carpathian, or Western origin, but the haplotype trees did not support these assignments. Colors of 'Unclear' loci indicate origin inferred from allele-frequency analyses. Hatched regions represent uncertainty in the scoring of haplotype trees. *P*-values are based on chi-squared tests.

Note on Figure 2: We noticed the requirement in quotes *"Convert all bar graphs to box-and-whisker or dot-plot format to show data distribution."* in the journal revision checklist. We believe that these types of graphs are not appropriate for the categorical data compared here. Figure 2 was updated by including the results of the neutral panel.

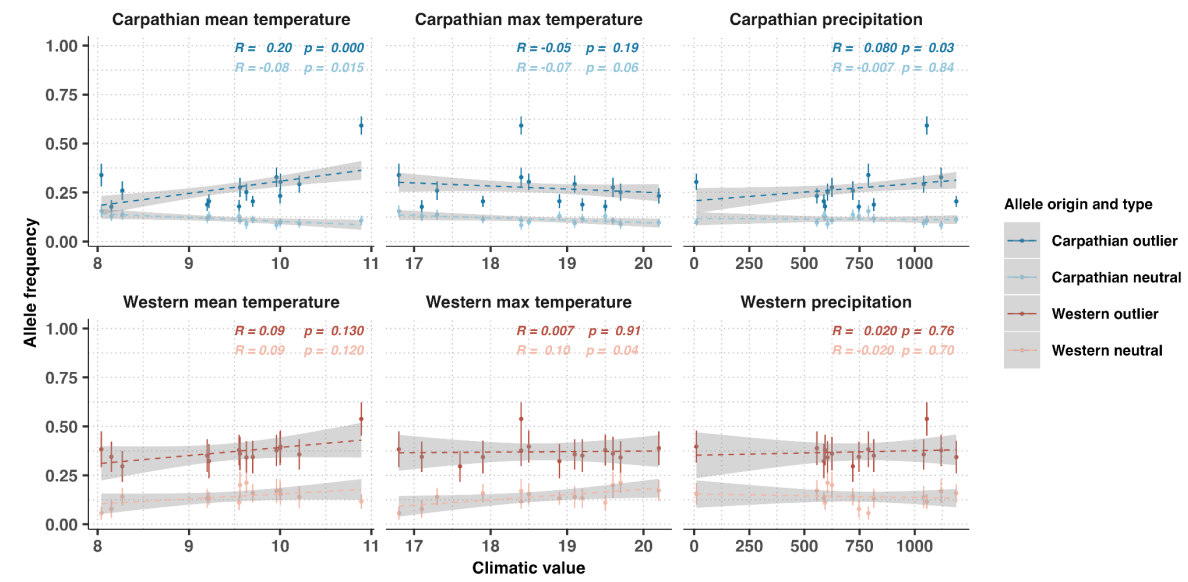

Figure 4: Allele frequencies of outlier or neutral alleles with Western or Carpathian refugial origins across Britain in relation to climatic variables. Population frequencies of Carpathian outlier alleles showed significant positive associations with mean annual temperature and precipitation. In contrast, Western outlier alleles were not significantly associated with climate. Patterns in neutral alleles reflect colonization history, including negative associations between Carpathian neutral alleles and mean annual temperature and positive associations between Western neutral alleles and maximum temperature of the warmest month[17,30]. Carpathian and Western variants at outlier loci consistently occur at higher population frequencies compared to neutral alleles.

<table>
<tr>
<td></td>
<td>

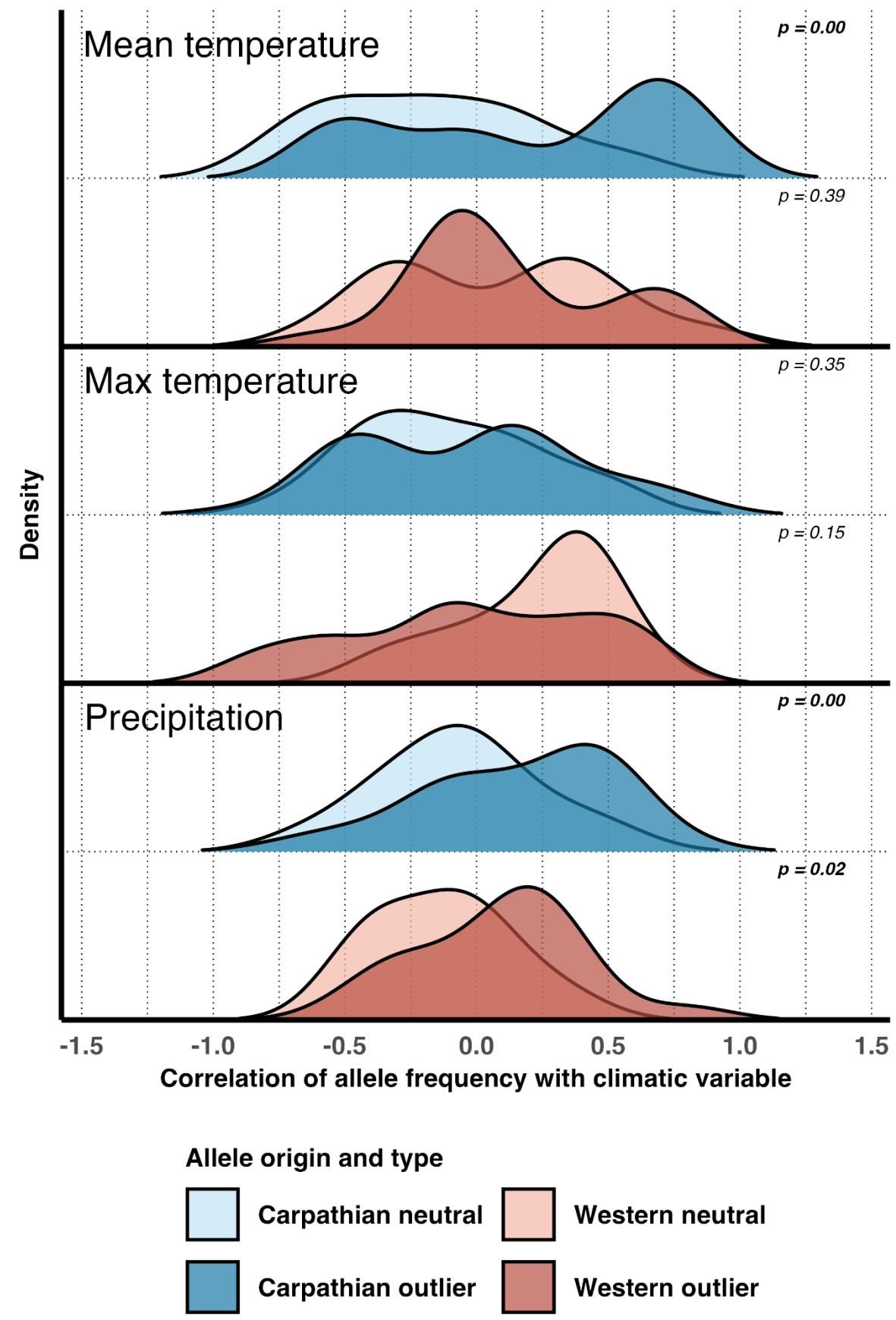

Figure 5. Distributions of correlation coefficients between climatic variables and population allele frequencies. In British bank voles, Carpathian outlier alleles showed stronger positive correlations with mean annual temperature than neutral Carpathian alleles, whereas distributions of both Carpathian and Western outlier alleles were more positively correlated with annual precipitation than neutral sets. Differences between the distributions of correlation coefficients for neutral and outlier alleles from each refugium were assessed using Kolmogorov–Smirnov tests.

</td>
</tr>
<tr>
<td>2. writing – the authors do a good job of setting up the structure of the paper with their hypotheses, however these are rarely revisited in the results section. It would help if in results sub-section it refers briefly back to whether it supports the original hypothesis.</td>
<td>Based on this feedback, in several places we added reference to the hypothesis being tested. For example, Lines 136–138: "Most SNPs associated with climate adaptation occur as widespread polymorphisms present in mainland Europe as well as in Britain, supporting the hypothesis that these variants</td>
</tr>
</table>

	originated on the continent rather than arising de novo *post-colonization*."<br><br>Additionally on Lines 328–332: "*However, across all three tests of climate association, candidate adaptive alleles originating in the Carpathian refugium are more strongly positively correlated with mean annual temperature than either neutral Carpathian alleles (which tend to be negatively correlated, as expected by phylogeographic history) or Western alleles, contrary to our hypothesis.*"
3. L41 – and population size	We have now added this (line 33): *"A species' phylogeographic history—its past population size and movements—is a primary driver of the spatial distribution of genetic diversity*[1–4]*."*
4. L71 – there is no real way to know those were refugia without fossil data	We have added clarification to indicate that the Western refugium location is simply suggested (line 67): *"later from the Western refugium (presumed to be located in the Dordogne region in France*[22,23]*)"*
5. L245-8 – this may be true but it is not convincing without comparing these distribution to the association of the non-refugial (i.e. widespread) alleles to the same climatic variables. In other words, it lacks necessary statistical context without adding the widespread loci to Figure 4.	The inclusion of a comparative panel of neutral loci (see response to the reviewer's first request) provides important statistical context for this interpretation. The results indicate that Carpathian outlier alleles show a significant positive correlation with mean annual temperature, whereas Carpathian neutral loci show the opposite trend. Contrary to our expectations, the overall set of Western outlier alleles does not show a significant positive correlation with mean annual temperature (Fig. 4) relative to Western neutral alleles, although some individual loci do exhibit strong positive correlations (e.g., the upper tail of the distribution in Fig. 5).<br><br>We respectfully disagree with the suggestion to add widespread loci to this analysis. Unlike alleles with inferred Carpathian (colder and wetter) or Western (warmer and drier) origins—which provide the desired contrast for our study—widespread alleles lack a clear a priori expectation for selecting one variant over another at a locus, and their deviations from background alleles are difficult to

	interpret. By contrast, the panel of neutral background alleles we employed has been assigned to specific geographic origins wherever possible, thereby providing a meaningful baseline against which to evaluate putative adaptive loci.
6. L256-7 – Given that Fig S5 plots Dxy, I don't see how this is evidence that bottlenecking did not occur. It seems the authors really need to run SMC++ or skyline plots to look at population-specific change in Ne over time and/or spatial patterns of genetic variation to test for this (or at a minimum run Tajima's D on the non-candidate loci)	As suggested, we ran Tajima's *D* analysis on the neutral (i.e., non-candidate) loci, and it shows evidence for population growth but no evidence of a bottleneck. This is now included in the manuscript on lines 173–175 in quotes here:<br><br>*"Tajima's D analyses supported a scenario of population expansion without subsequent contraction (Fig. S8), suggesting widespread retention of genetic variation across the genome."*
7. L277 – the authors may also consider if the refugia were stable over subsequent glaciations, then repeated admixture events may have sustained variation over time and manifest today as 'widespread' with deeper coalescence times	We agree that this is likely the case and that the older ages inferred for many of the 'widespread' refugia support this idea. To address this in the manuscript, we provide additional clarification in two sentence:<br>lines 268–270: "*documented ancient admixture between bank vole lineages in mainland Europe*[15]*, likely explains the high frequency of widespread polymorphisms with deeper coalescence time in our dataset.*"<br>lines 292–295 "*These repeated admixture events during interglacial periods, many involving the Carpathian refugium as one of the source population, likely facilitated the spread of alleles between refugia, contributing to the widespread polymorphisms observed today*."
8. L287 – again, having population-specific Ne estimates would help clarify this.	We further support the discussion of Carpathian origins of variation by contrasting results from the neutral set of SNPs, which support a similar pattern of higher Carpathian contributions. Previous studies also suggest that the Carpathian lineage harbors greater genetic diversity than the Western lineage (Horníková et al., 2021). These findings imply that the Carpathian lineage may simply maintain a larger pool of standing genetic variation and is therefore more likely to serve as a source of adaptive genetic variants

<table>
<tr><td></td><td>(Hermisson & Pennings, 2005; Hendry, 2013). We have expanded on this in the third paragraph of the discussion (lines 277–296).</td></tr>
<tr><td>9. L404 – I believe in the SI it says 50kb on either side (100kb total?)</td><td>It is close to 100 kb total. The region is 50 kb upstream and downstream of a target SNP (1 bp). We have provided additional clarification of this in the methods (lines 405–406): “For each SNP, we constructed trees from a ~100 kb window (±50 kb) surrounding the focal site (100,001 bp total)”</td></tr>
<tr><td>10. Fig 3 – I think it would be helpful to show the distribution for ‘widespread’ alleles on this chart as well for context.</td><td>We have added the widespread alleles to this figure, which is now shown in a log scale.
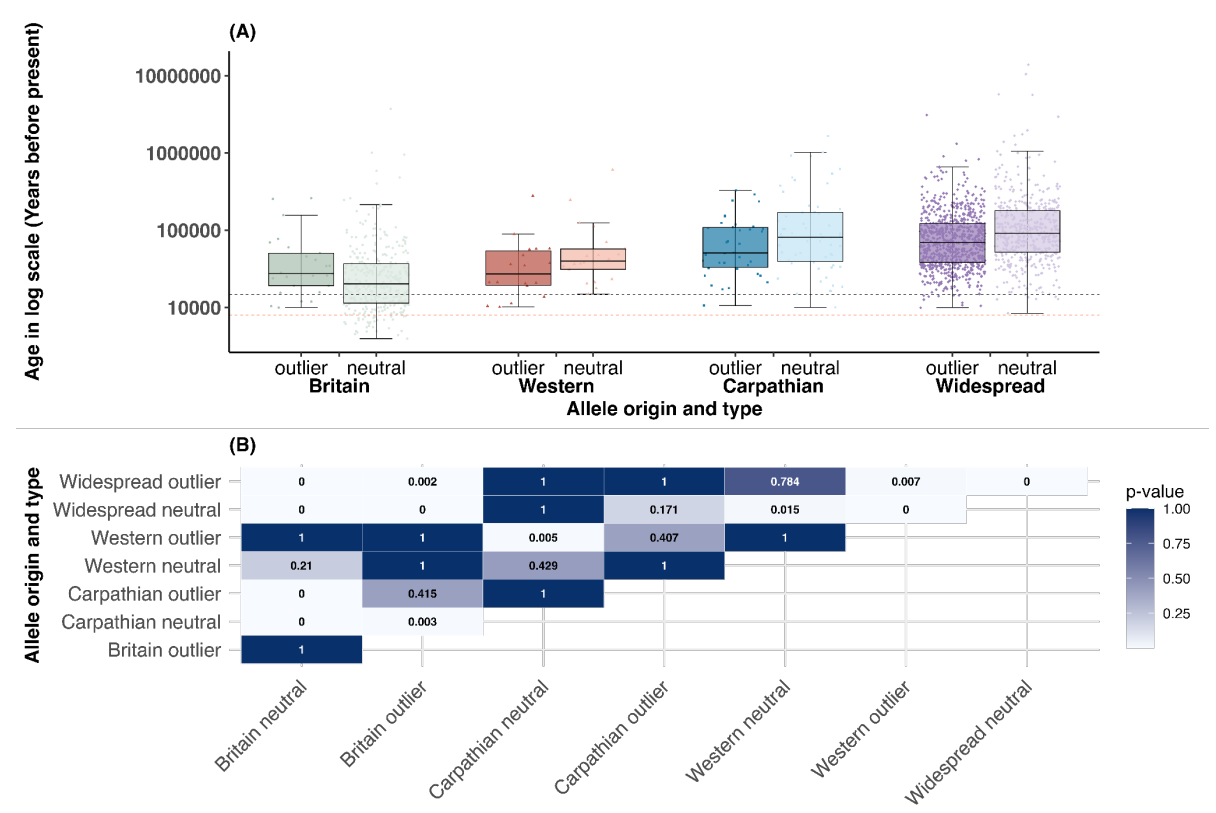

Figure 3. Ages of alleles of based on assigned geographic origins. (A). The ages of alleles (points) grouped by inferred geographic origin, with overall distribution shown as box-and-whisker plots. Ages were estimated by converting coalescent time in generations to years before present (YBP) assuming a generation time of 0.5 years. The dark dashed lines mark the period of Britain's colonization, beginning ~14,700 YBP, and the lighter dashed line reflects the submersion of the Doggerland connection to mainland Europe. (B) Heatmap of p-values from Kruskal-Wallis tests with post hoc Dunn’s test comparing allele ages.

Note on Fig 3: This figure now includes the ages of the widespread alleles, the ages of the neutral alleles, and p-values as requested by Reviewer 3.</td></tr>
<tr><td>11. Supplemental Methods–
- Bbduk version?</td><td>BBDuk is part of the BBTools package. We used BBTools version 38.88 and now added</td></tr>
</table>

	the information as*BBDuk in BBTools v.38.88*" to the supplementary information.
12. Bwa mem flags	BWA-MEM was executed using the default settings. This information has now been included in the supplementary information.
13. The rho used to calculate recombination rate was from mouse, but where was the Ne taken from? The Ne can be calculated per population or per species, how would it be impacted by the change of Ne that come from populations merging and splitting during glaciations? If the age of allelic origin is important to the story (which it seems to be) then the estimation of r here is as well. If it was taken from mouse, that is not a reasonable choice.	Estimates of recombination rate (*ρ)* and *Ne* were used to calculate *r*, and both were taken from the mouse due to the lack of species-specific estimates for either these parameters or *r* for the bank vole. While a species' specific estimate would be nice, this is the most biologically reasonable option available. We provide additional clarification on this in the methods (lines 422–424): "*Coalescent ages were estimated using RUNTC v.0.0.1*[44] *for both neutral and outlier datasets assuming a per-base per-generation recombination rate (r) of* 6×10^{-9} *(calculated as r = ρ/4Ne, with Ne = 386,000 and ρ = 0.0091 from Mus musculus*[67]*).*"
14. Where was the generation time of 0.5 years taken from?	Generation time was assumed to be roughly 1/2 year, based on the breeding season and average breeding onset. (*Handbook of the Mammals of the World*, Wilson (lines 424–427): "*Coalescent times (in generations) were converted to years before present (YBP) assuming a generation time of 0.5 years, based on breeding seasonality (March–October; males breed from ~2 months of age, females from ~2–5 months)*[16]*.*"

**Reviewer 2 comments**	**Response**
1. This study builds upon a recent study by many of the same authors (Markova et al. 2023) that seeks to investigate the origins of genomic variation linked to climate in present-day British bank voles. These studies are starting to build upon classic phylogeographic studies to look at how past standing variation might influence future adaptation in populations to ongoing change. The main limitation is likely the limited geographic scope and the focus on Britain (which was the	Thank you. We agree that broader geographic coverage is important. Building on Markova et al. (2023), we focused on Britain because it provides a tractable system with strong data depth and a well-characterized history, which enabled us to uncover mechanisms. We are actively extending this work to other regions.

focus of the previous paper also). Should there be concerns in regions that are more likely to be impacted by climate in the more southerly regions for example and the implications for more northerly populations in Scandinavia. I certainly see the value of this study however and how it has implications well beyond the current study system.	
2. The keywords refer to the genus as Myodes but the main ms to Clethrionomys. Now, this may be deliberate given the back and forth with the classification of this species in recent years so just check if you meant this or if a mistake.	This was deliberate, as there is some debate on the correct genus to use (even within our group of authors). To further reduce confusion, we have included *Clethrionomys* in the keywords.
3. It is somewhat confusing as to why samples representing the Western lineage have been chosen that are quite distant from the proposed location of the actual refugium. This is an incredibly well-studied phylogeographic system (probably the best-studied in Europe at least in terms of a mammal) with a huge number of samples that should have been available. Was there a reason that samples could not have been selected closer to the proposed refugium in a similar way to the Carpathian refugium? If direct comparisons are being made, the sampling is not exactly uniform between both and it's unclear why.	As the reviewer indicates, this is an exceptionally well-studied system, and we used that literature and our existing knowledge of the system to determine sampling localities. These 'refugial' populations were chosen because they show minimal secondary admixture (i.e., admixture post-glaciation), compared to several closer to the inferred refugial location. We have included is in the methods (lines 363–364): *"Continental populations were chosen for their minimal secondary admixture, particularly in the Western refugium."*
4. Lines 182-186. Would it not be possible that at least some of the alleles unique to Britain could also have been missed given the limited number of individuals screened from the mainland. This obvious possibility only hinted at and the sampling design/locations should be critiqued here rather than just stating that they may have been missed. This goes back to the point above that that is the main concern with the ms: the sampling is fairly limited from the two refugia representing the mainland. This definitely will impact some of the findings and inferences drawn here.	It is likely that at least some of the alleles unique to Britain are actually variants from the European mainland. Indeed, the allele ages suggest this scenario. Only 6 alleles are estimated to have arisen after the submersion of Doggerland, out of 40 indicated as possibly British by the allele frequency and genealogy analyses. This scenario is further addressed in the discussion in lines 315–317 in quotes here: "*Most alleles at both candidate adaptive loci and neutral loci inferred as British predate 14,700 YBP, the earliest estimated colonization of the island*[5,6,17]*, suggesting they originated in mainland Europe but were un-sampled or lost post colonization.*"<br><br>While this impacts some of the inferences (i.e., the average age of British alleles is likely

	overestimated in comparisons shown in Fig. 3), it has little impact on the overall aims of the paper. Further, it underscores how little impact 'new' mutations have on climate adaptation in this context.

<table>
<tr><th>Reviewer 3 comments</th><th>Response</th></tr>
<tr><td>1 Thank you for submitting your manuscript on the origins of adaptive genomic variation in bank voles. While the topic is potentially very interesting, there are several areas that require thorough revision before it can be considered for publication.

One major improvement would be to provide a picture of the target animal species. Including information about their habitat, behavior, and ecological aspects would greatly assist readers in understanding the context and significance of your study. This additional information will enhance the overall clarity and impact of your findings.</td><td>We have added a photo of the bank vole to Figure 1. We also describe the general habitat of the bank vole in the Introduction (lines 62–64): "The species spans habitats across Europe and Asia[16], from coniferous forests and shrublands to bogs, hedgerows, and forest–steppe transitional zones, and, with a diverse range of historical climates."
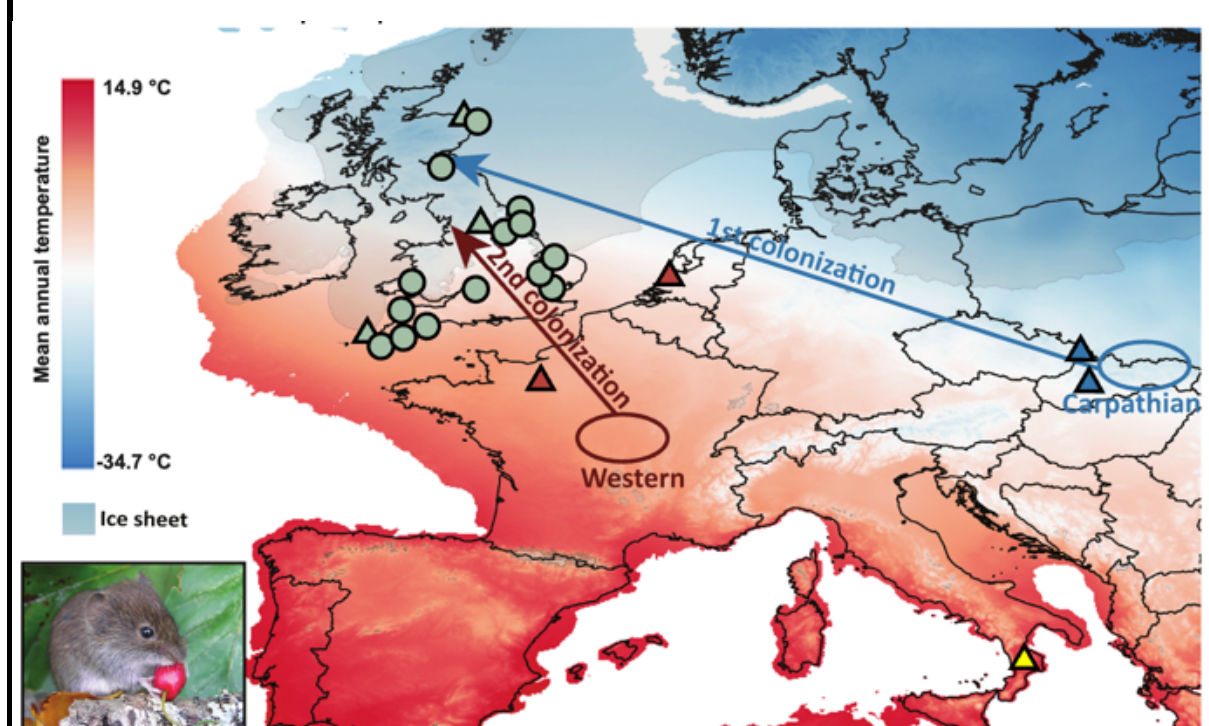

Figure 1. Population and environmental context. Population locations of the study species, the bank vole (inset image), relative to the Doggerland connection, the gradient of annual mean temperatures during the Last Glacial Maximum[28], and the extent of the ice sheet at approximately 18,000 YBP. Triangles: populations analyzed for allelic origin and age from Britain (pale green), continental populations used as references for the Carpathian (blue) and Western (red) refugia, and a Calabrian population in southern Italy (yellow) as an outgroup[29]. These continental populations were selected as the most representative un-admixed populations from the respective refugia[30]. As such, geographic positions of the populations do not necessarily correspond to the locations of the glacial refugia. Filled circles in Britain represent population samples included in analyses</td></tr>
</table>

<table>
<tr><td></td><td>evaluating the relationships between allelic origin and climate. Doggerland connected Britain and the European mainland until ~ 8,200 YBP.</td></tr>
<tr><td>2. Furthermore, I noticed that the figures included in this manuscript are poorly prepared and some of them could benefit from being compiled into a single figure. By consolidating these figures, you can present your data more effectively and improve readability for readers.</td><td>All figures in the main body of the manuscript have been updated. They are included in other responses to reviewers.</td></tr>
<tr><td>3. Additionally, it is important to address an issue regarding the presentation of your main results. Currently, they are hidden within supplementary files which significantly diminishes their importance. To ensure that your key findings receive proper attention, consider integrating them into the main body of the manuscript.</td><td>The main results are in the main body of the manuscript, and we have reviewed all results which are included in the revision to ensure that all essential results and data are included. We have opted to keep several supplemental tables and figures for full transparency with respect to the data.</td></tr>
<tr><td>4. Moreover, I observed that some figures presenting statistical results lack legends indicating their significance. It is crucial to include clear explanations or captions alongside these figures so that readers can understand their implications without confusion.</td><td>We have updated the figures and legends so that indications of statistical significance are clearer.

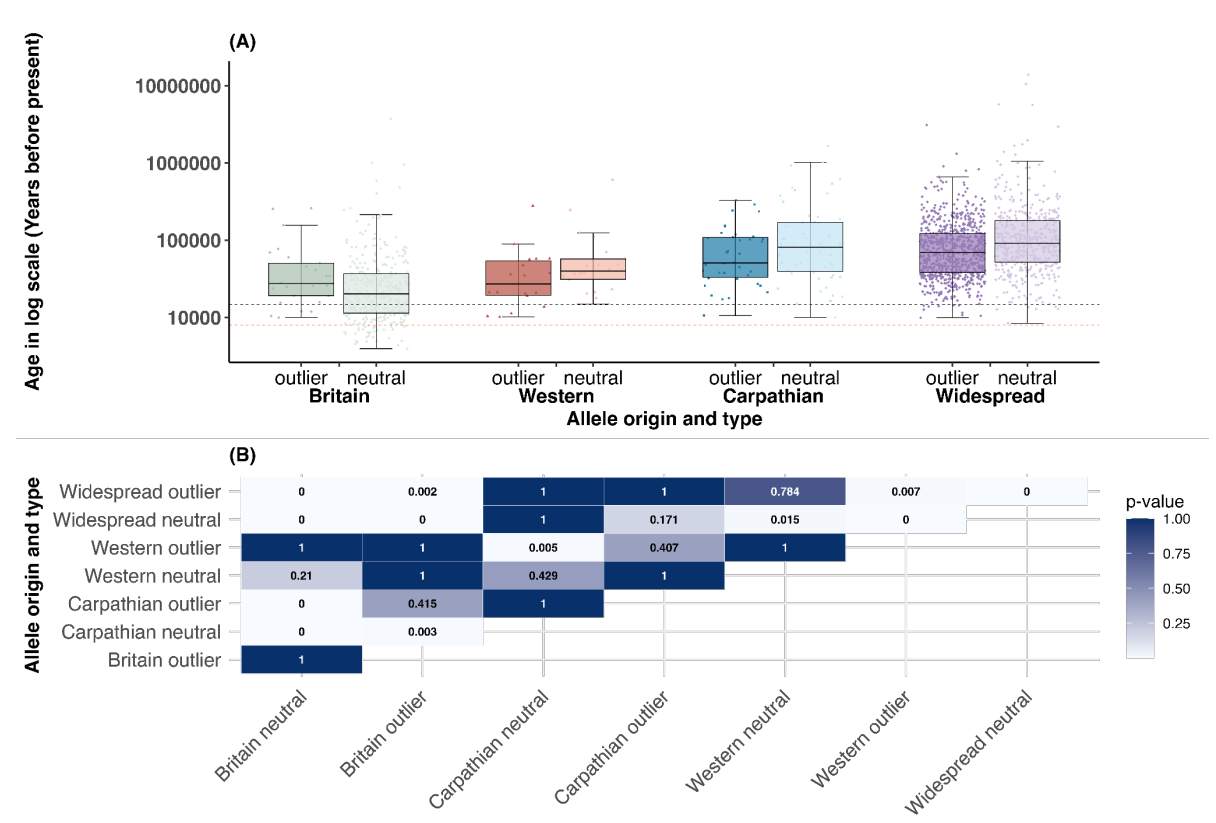

Figure 3. Ages of alleles of based on assigned geographic origins. (A). The ages of alleles (points) grouped by inferred geographic origin, with overall distribution shown as box-and-whisker plots. Ages were estimated by converting coalescent time in generations to years before present (YBP) assuming a generation time of 0.5 years. The dark dashed lines mark the period of Britain's colonization, beginning ~14,700 YBP, and the</td></tr>
</table>

<table>
<tr>
<td></td>
<td>lighter dashed line reflects the submersion of the Doggerland connection to mainland Europe. (B) Heatmap of p-values from Kruskal-Wallis tests with post hoc Dunn's test comparing allele ages.</td>
</tr>
<tr>
<td>5. Lastly, there are still areas throughout the manuscript where confusion persists and needs to be addressed. For example, in one instance it states in the legend "Carpathian alleles were significantly more negatively correlated with mean annual temperature than Western alleles with p-value = 0.01 according to Mann–Whitney U test," but upon reviewing Figure (b), I found that the correlation coefficient for 'Carpathian' was actually lower than that for 'Western'.</td>
<td>We have completely re-done these analyses, drawing contrast between both adaptive candidates with origins which have been inferred in each refugium and with their respective neutral counterparts. As a result, this entire section has been substantially revised and those confusing aspects removed entirely.

The figure displaying those results have been substantially modified, and replaced Figure 5 (below):

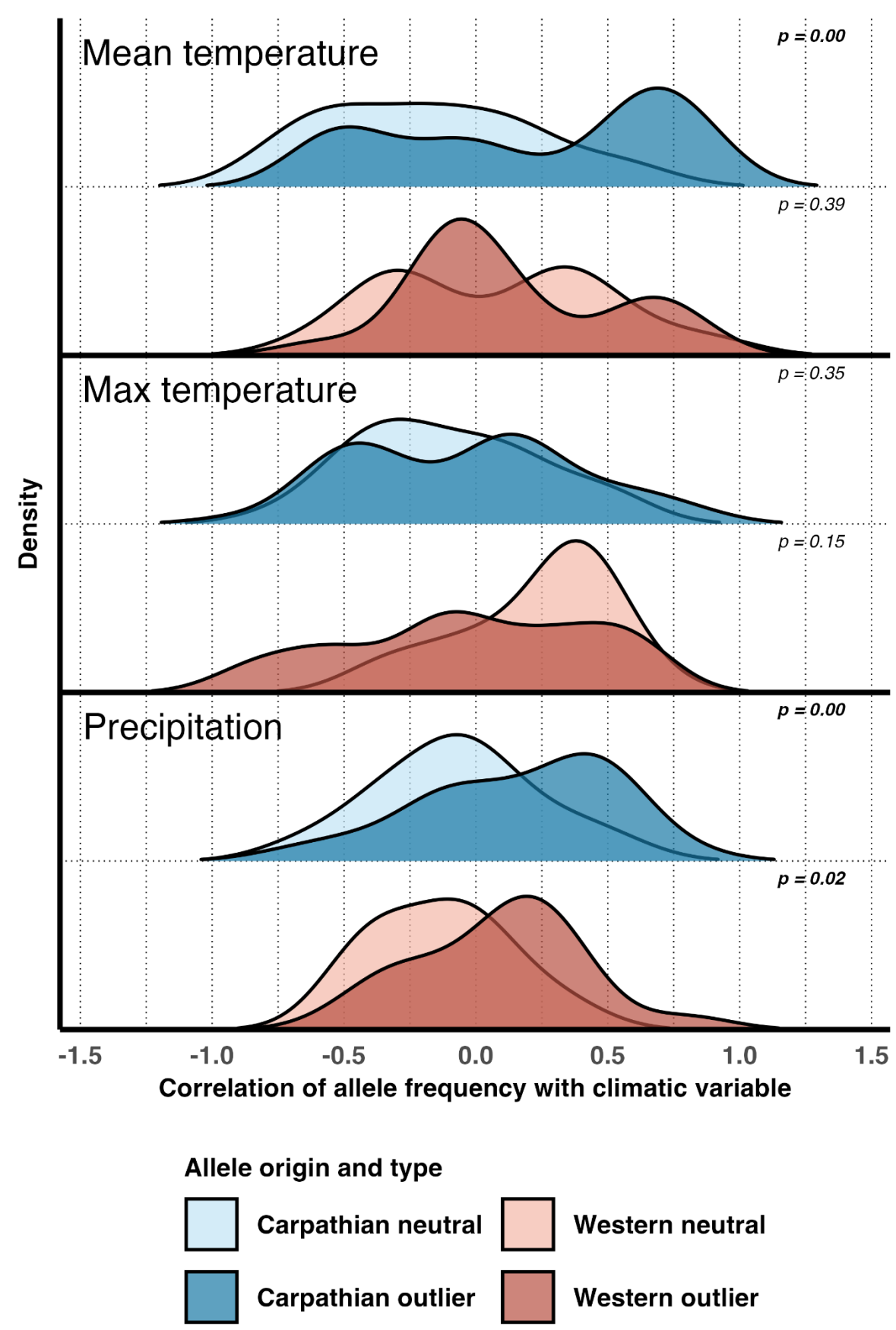

Figure 5: Distributions of correlation coefficients between climatic variables and population allele frequencies. In British bank voles, Carpathian outlier alleles showed</td>
</tr>
</table>

	stronger positive correlations with mean annual temperature than neutral Carpathian alleles, whereas distributions of both Carpathian and Western outlier alleles were more positively correlated with annual precipitation than neutral sets. Differences between the distributions of correlation coefficients for neutral and outlier alleles from each refugium were assessed using Kolmogorov–Smirnov tests.

Dear Dr. Bunton-Stasyshyn,

Thank you for the extension on our manuscript. We have completed both the requested revisions and updates related to the issue we identified with several of our sample identifications. The resulting manuscript reflects these changes, as detailed in the response below.

The biologically significant trends in the dataset, and the conclusions we draw from them, have not been greatly altered by the data correction. We have provided a side-by-side comparison of all the affected tables and figures at the end of this letter, to allow you to more easily judge the magnitude of the difference.

With appreciation,
Marcos

–

Dear Mr da Cruz,

Your manuscript entitled "Origins of adaptive genomic variation in a wild rodent" has now been seen again by our referees, whose comments appear below. In light of their advice I am delighted to say that we are happy, in principle, to publish a suitably revised version in Communications Biology.

Please address all of the remaining comments from the Reviewers and when re-submitting, include a point-by-point rebuttal and a manuscript highlighting all changes.

We therefore invite you to revise your paper one last time to address the remaining concerns of our reviewers. At the same time we ask that you edit your manuscript to comply with our format requirements and to maximise the accessibility and therefore the impact of your work.

* Please see the attached document for editorial requests for the final version (.docx file). Please ensure a completed version of this file is uploaded as a Related Manuscript with your final submission.

* Please review our final submission file checklist to ensure all necessary files are present with your final submission and to avoid delays in accepting your manuscript. For your reference, a style and formatting guide is available here and includes all of our style requirements.

It is important that you pay careful attention to the requests in these documents to avoid a delay in formal acceptance of the article.

If you have any questions or concerns about any of our requests, please do not hesitate to contact me.

Open access
Communications Biology is a fully open access journal. Articles are made freely accessible on publication. For further information about article processing charges, open access funding, and advice and support from Nature Research, please visit https://www.nature.com/commsbio/open-access
At acceptance, you will be provided with instructions for completing the open access licence agreement on behalf of all authors. This grants us the necessary permissions to publish your paper. Additionally, you will be asked to declare that all required third party permissions have been obtained, and to provide billing information in order to pay the article-processing charge (APC).

Please use the following link to upload your revised files:

https://mts-commsbio.nature.com/cgi-bin/main.plex?el=A5Cx2Jbg3B3BovB5I7A9ftd6eoiMs5OQFlUZnEjHEAZ

** This url links to your confidential home page and associated information about manuscripts you may have submitted or be reviewing for us. If you wish to forward this email to co-authors, please delete the link to your homepage first **

We hope to hear from you within two weeks. If you expect the process to take longer than one month, please let us know.

Congratulations on an excellent paper!

Best regards,

Rosie Bunton-Stasyshyn, PhD
Associate Editor
Communications Biology
orcid.org/0000-0002-1895-2240

and

John Mulley, PhD
Editorial Board Member
Communications Biology
orcid.org/0000-0002-1537-7316

PS: At acceptance, the corresponding author will be provided with instructions for completing the license on behalf of all authors. This grants us the necessary permissions to publish your paper. Additionally, you will be asked to declare that all required third party permissions have been obtained, and to provide billing information in order to pay the article-processing charge (APC).

<table>
<tr><th>Reviewer 1 comments</th><th>Author’s response</th></tr>
<tr><td>I was the previous reviewer who requested the test on background neutral loci, and I’m very glad the authors took that feedback seriously. The paper is now much stronger, and the results are compelling. It addresses several fundamental questions in population genetics that are typically only explored through simulations, so I believe this study will serve as a valuable — and likely highly cited — empirical example. The new figures are clear and well presented. I have only minor suggestions below; overall, I am now very happy with and fully supportive of this manuscript.

I noticed that the Carpathian outlier loci are strongly associated with mean temperature but not maximum temperature (Figs. 4–5). This seems reasonable, as critical thermal minima and maxima may have harder physiological limits than other aspects of temperature response. Just a thought as to why this pattern might be expected.</td><td>Thank you for all the feedback on our manuscript. We agree it has substantially improved the paper. We have also added a sentence reflecting your suggestion on physiological limits, which we agree may be the case.

Added on lines 282–285: “Although Carpathian outlier loci are strongly associated with mean temperature, they are not strongly associated with maximum temperature (Figures 4–5). This may support the idea that thermal maxima, and possibly also minima, have harder physiological limits than other aspects of temperature response. ”
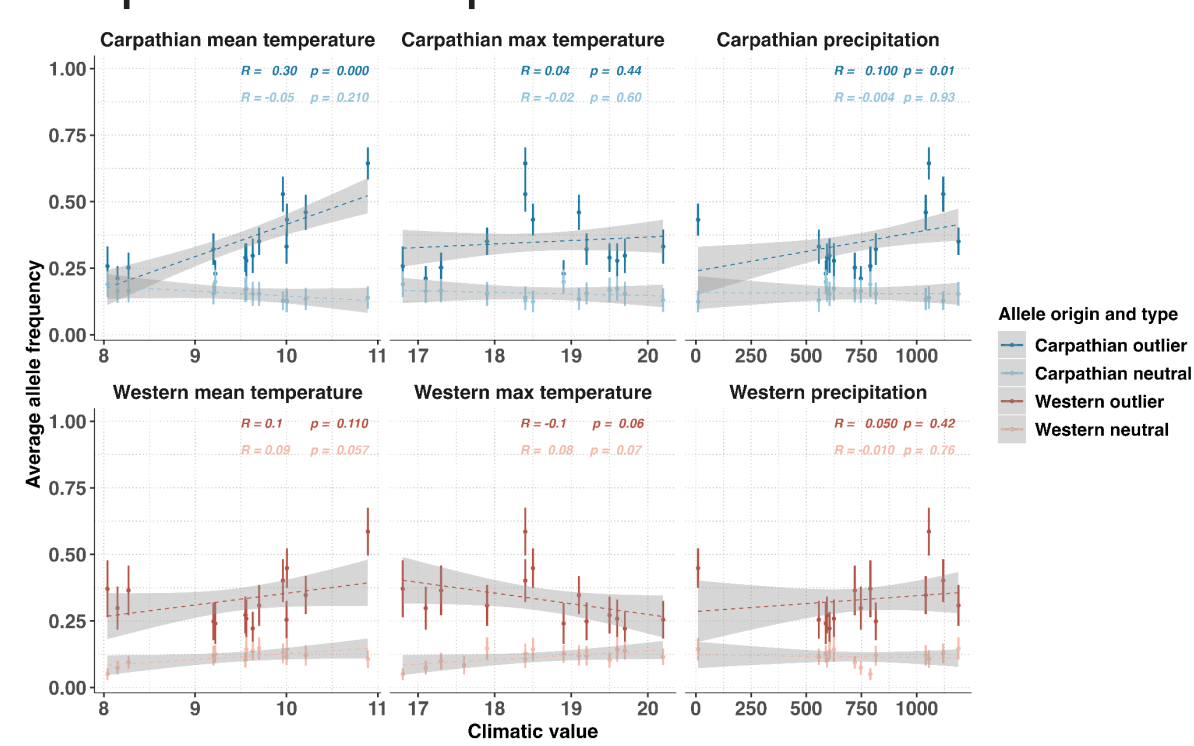
</td></tr>
</table>

<table>
<tr><td></td><td>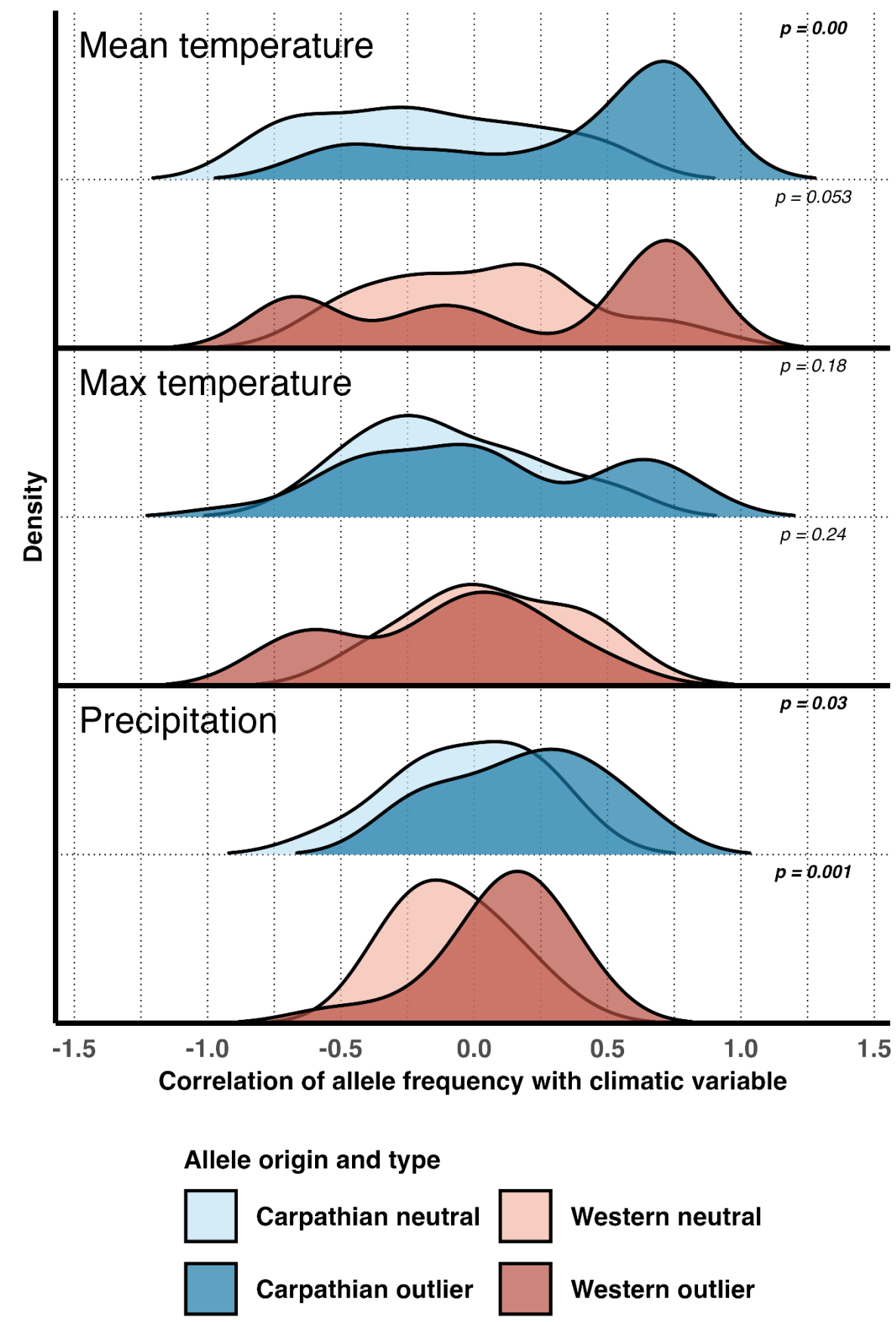
</td></tr>
<tr><td>L59 – suggest removing the comma after ‘resources’</td><td>The comma was removed (now line 57)</td></tr>
<tr><td>L103 – consider slightly rephrasing aim 1. You’re really testing whether refuge-derived allele-linked SNPs show a different spatial pattern than neutral ones, as would be an expected result of local adaptation/selection</td><td>Thank you for the suggestion. We want to avoid leading readers to anticipate spatial statistical analysis associated with Aim 1, but given your feedback have revised the aims as follows on L88-92: “ Our aims were to: (1) test if refugial origins of alleles at climate-linked SNPs differ from neutral expectations, (2) assess the relative contributions of the two refugia, and (3) evaluate the relationship between allele origin and present-day climatic conditions."</td></tr>
<tr><td>L118 – add a citation for allele surfing</td><td>L105 a citation for allele surfing has been added</td></tr>
<tr><td>L222-4 – great!</td><td>Thank you!</td></tr>
</table>

L262-4 – while true, your results actually show something stronger: despite differences in magnitude of alleles contributed, the temp- and precipitation-associated alleles (presumably under selection) have risen in frequency regardless of source. This is expected, but it's nice to see a real example of it.	Added in line 211–213: "Despite differences in number of unique alleles contributed, the climate-associated outlier alleles (i.e., those presumably under selection) have risen in frequency regardless of source."
L321 – for clarity, consider replacing upward with "younger" or "older"	L265 we replaced it to use the term older. It now reads "…which can skew the results towards older coalescent times."
L323-5 – great	Thank you!
L384 – consider adding something about 'null expectations based on Ne and population structure alone against which…"	Added to line 358-361 of the current document: "To provide a set of null expectations based on Ne and population structure alone against which to compare outlier results, we selected an additional set of 1,074 putatively neutral loci, randomly sampled across the genome, polymorphic in Britain, and located ≥50 kb away from the candidate loci"
Fig 1 – I suggest nudging the labels for colonization and Carpathian so they don't overlap the black lines, which makes it harder to read	The labels were moved as suggested:<br>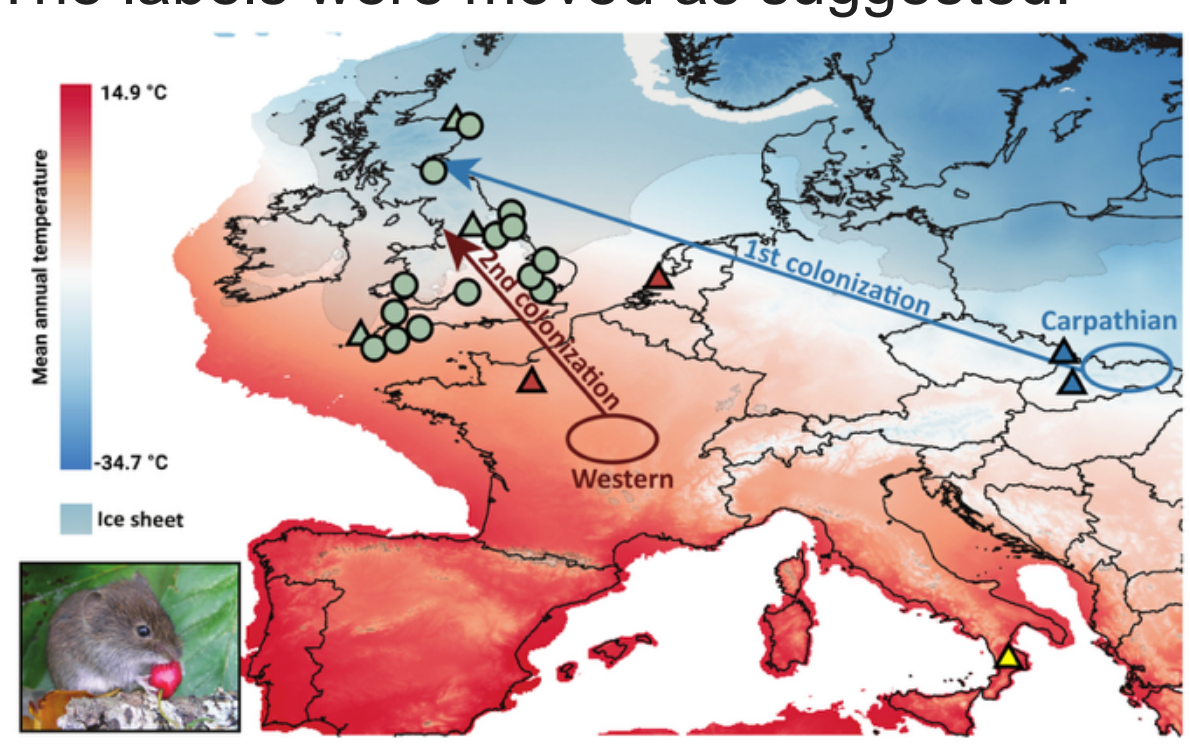
Fig 2 – great, really shows how strong the results are. This may be a conversion issue in the submission system, but the bold font has strange spacing—if not already I would suggest changing font to arial or Helvetica or something standard.	The font was changed to Helvetica. We also changed it to a dotplot as suggested by the editor:

<table>
<tr><td></td><td>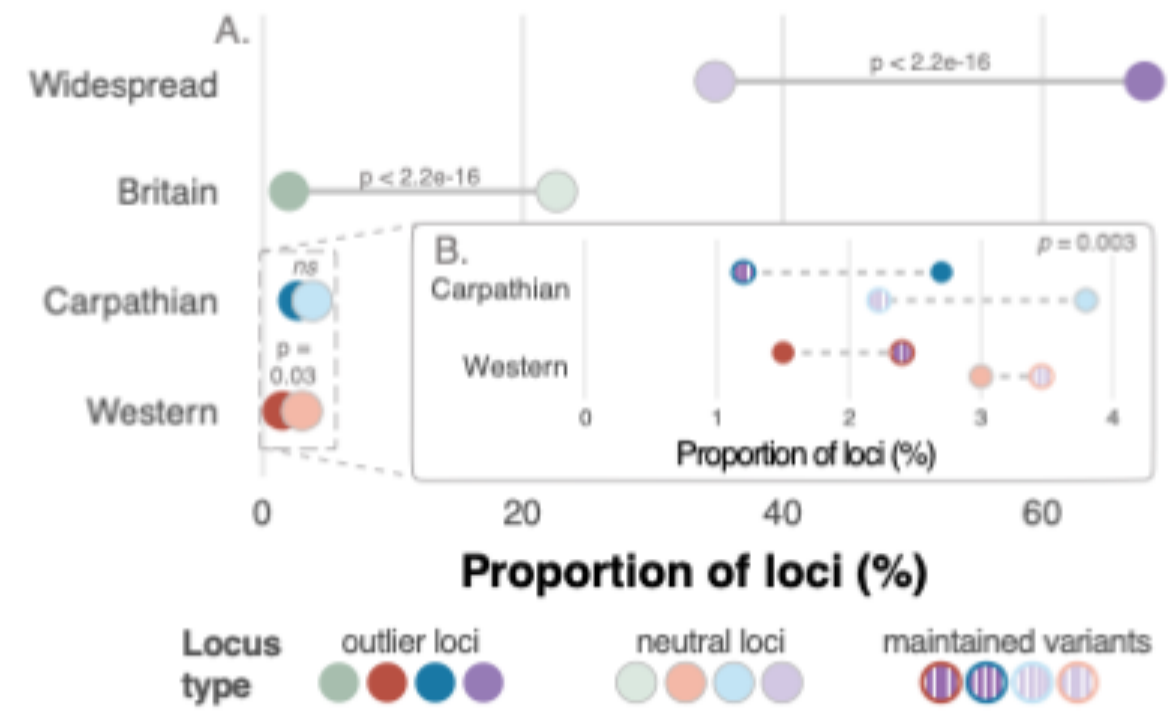
</td></tr>
<tr><td>Fig 3 – I suggest adding the significance results (where positive) to the figure itself—I see what the authors are doing but it’s strange to show a heatmap of p values since it’s more of a threshold rather than a gradient type of result. Or, just color the ones that are significant and leave the rest white etc.</td><td>We have changed the figure based on this suggestion by coloring the comparisons that were significant while leaving the rest white.
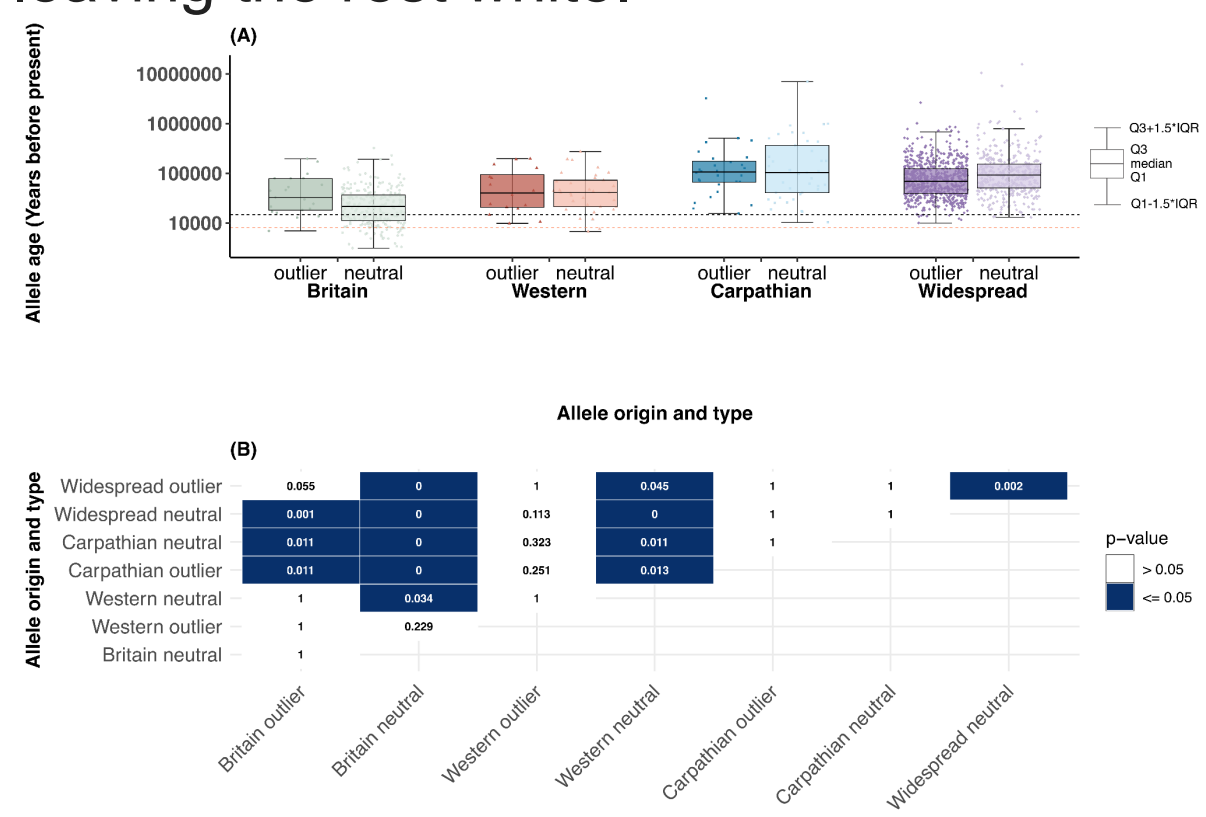
</td></tr>
<tr><td>Fig 4- great figure</td><td>Thank you!</td></tr>
</table>

As we were completing the requested revision we discovered that 4 of the samples in our dataset (all from mainland Europe) had been incorrectly assigned before we received our data from the sequencing lab. After thoroughly evaluating the extent of the problem and ensuring it was isolated to only 4 samples (out of the 152 total samples used for this study), we corrected this error and re-ran all the analyses. Differences between prior submission and this based on the corrected dataset are minor. For reference all impacted figures and tables are shown below.

Prior submission – Fig 2

Fig. 2

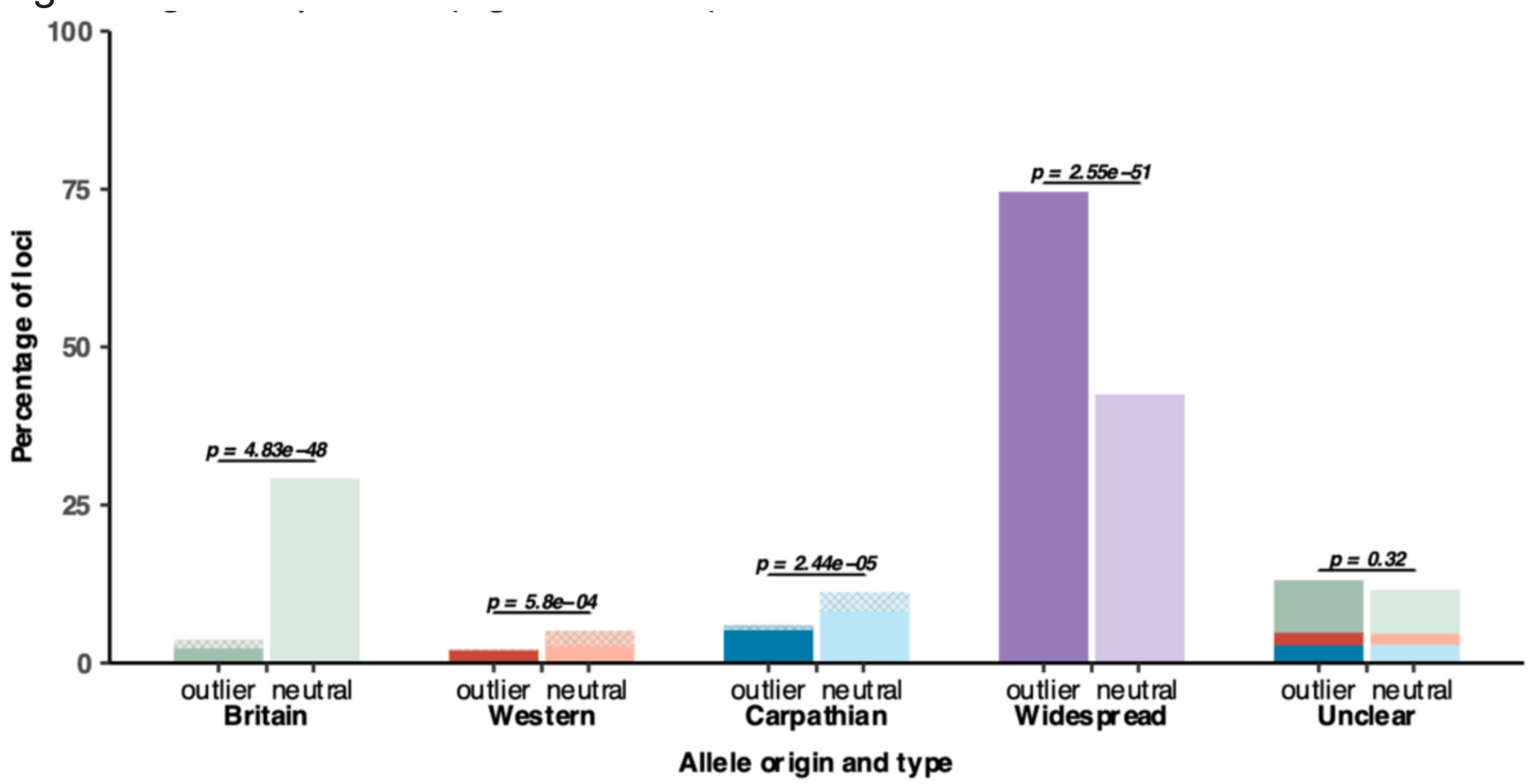

Revised dataset – Fig. 2 (*note, figure 2 is now presented as a dotplot as shown above in reviewer responses, but the version shown here provides the most direct comparison of results)*

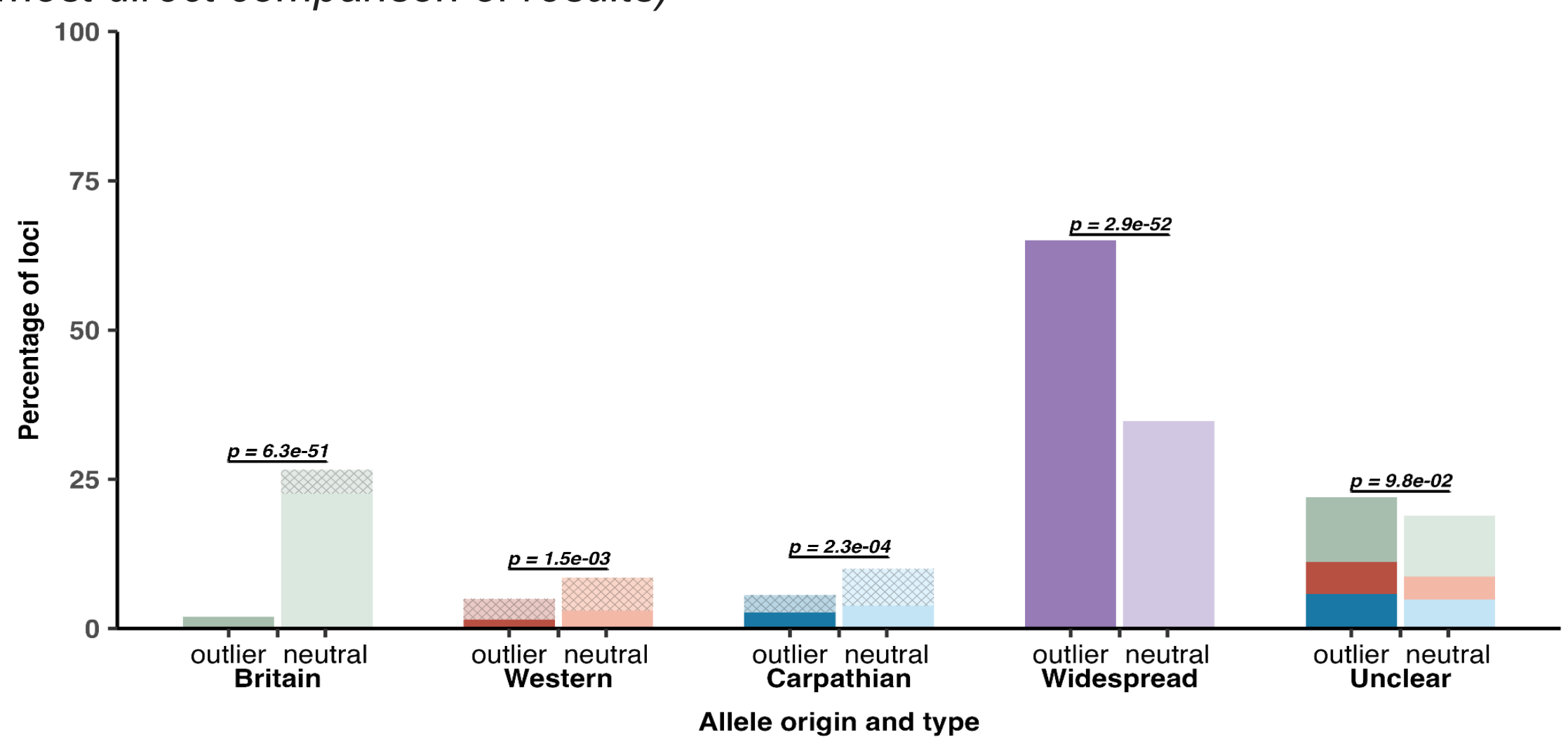

Prior submission – Fig 3

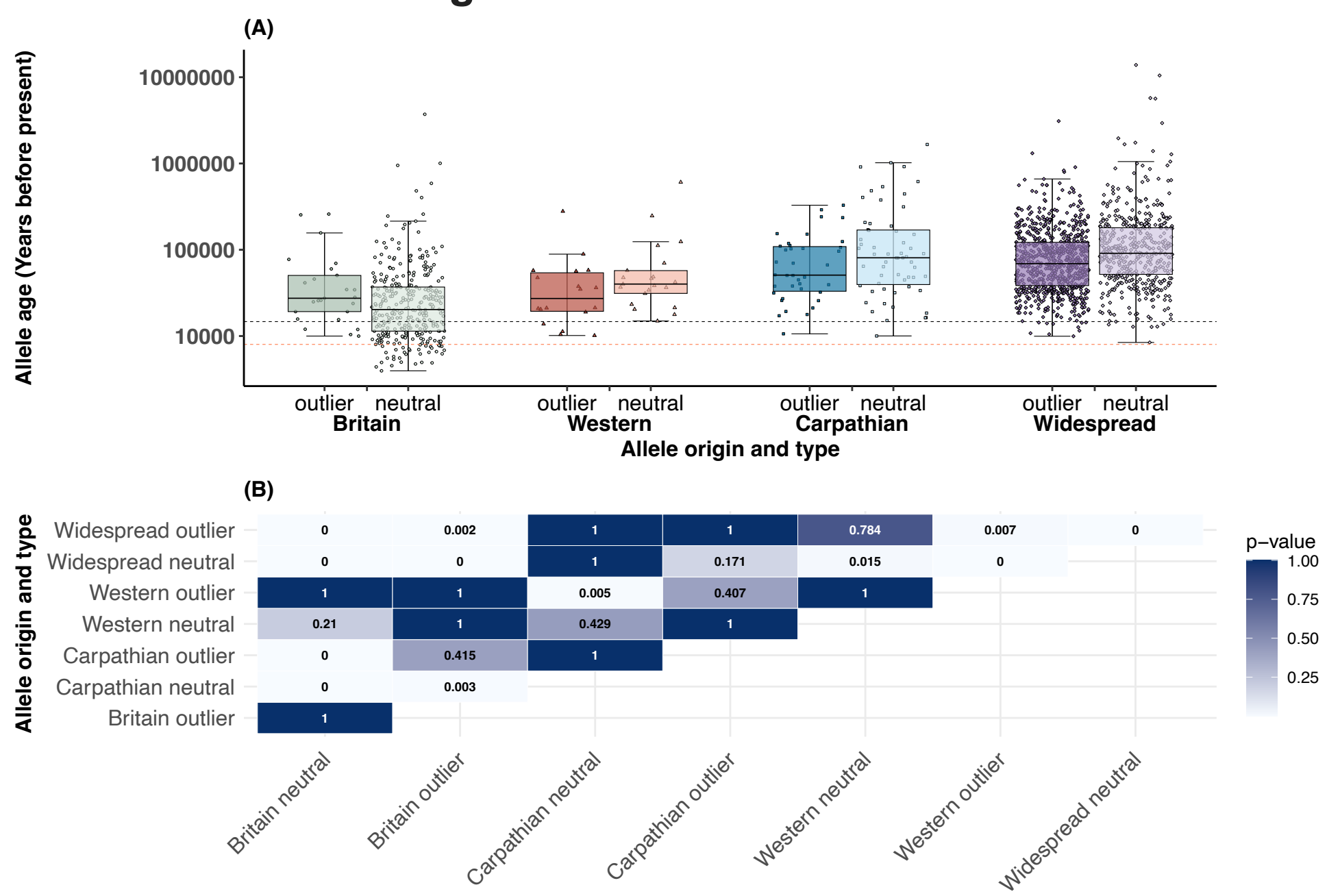

Revised dataset – Fig. 3 (note that the color is changed, as requested by Reviewer 1, and the order of the columns has also shifted to better align with the figure above)

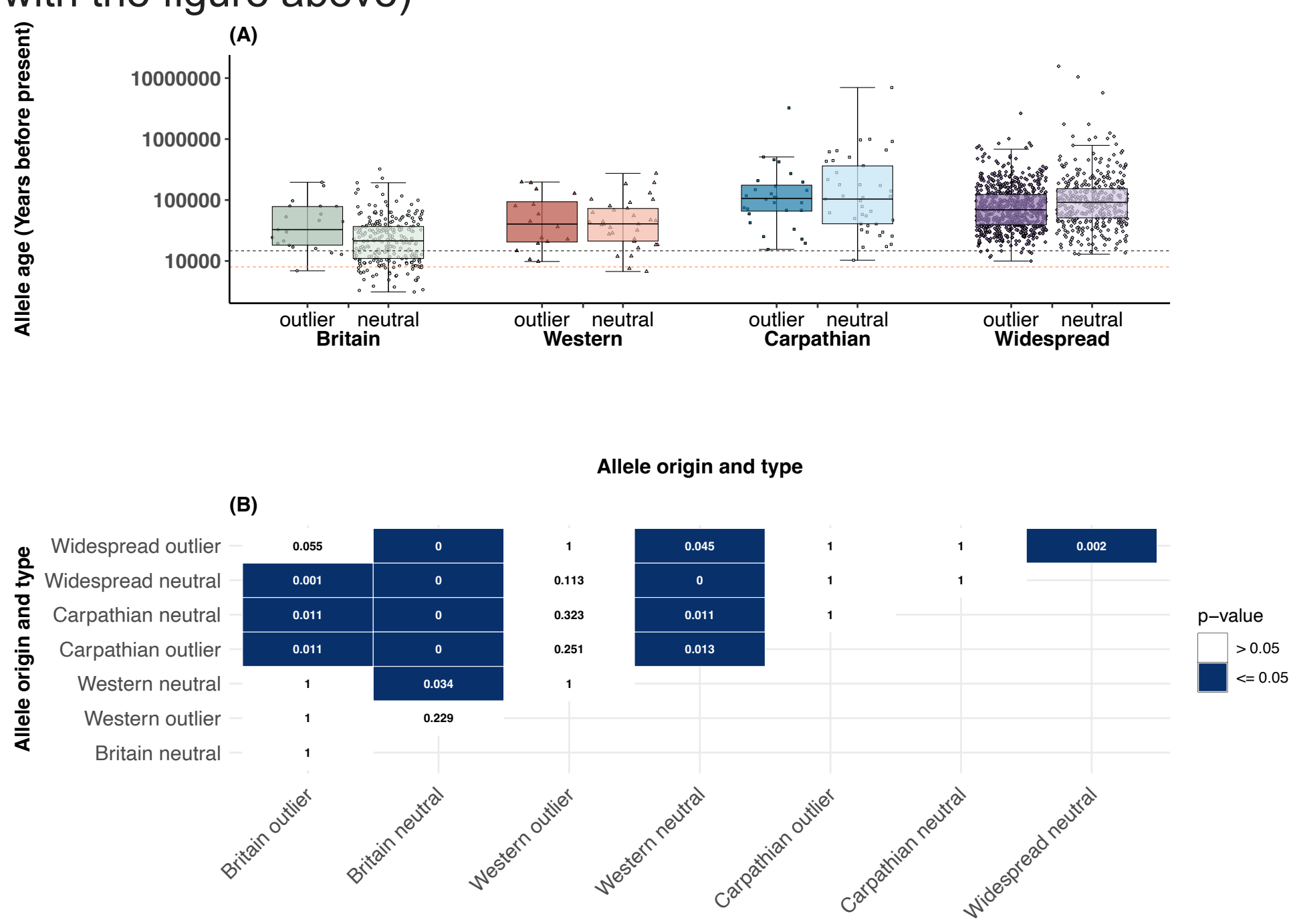

Prior submission – Fig 4

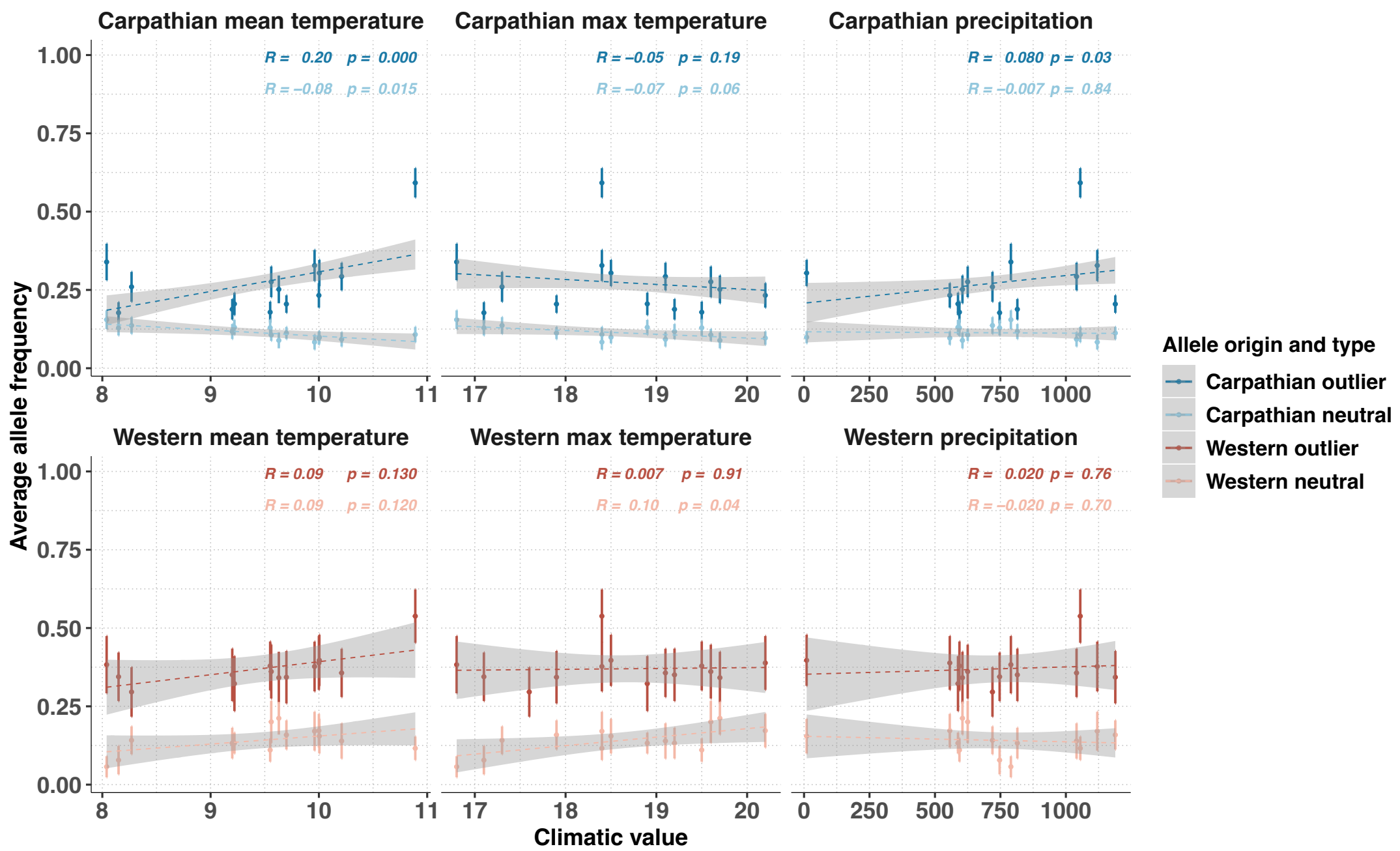

Revised dataset – Fig. 4

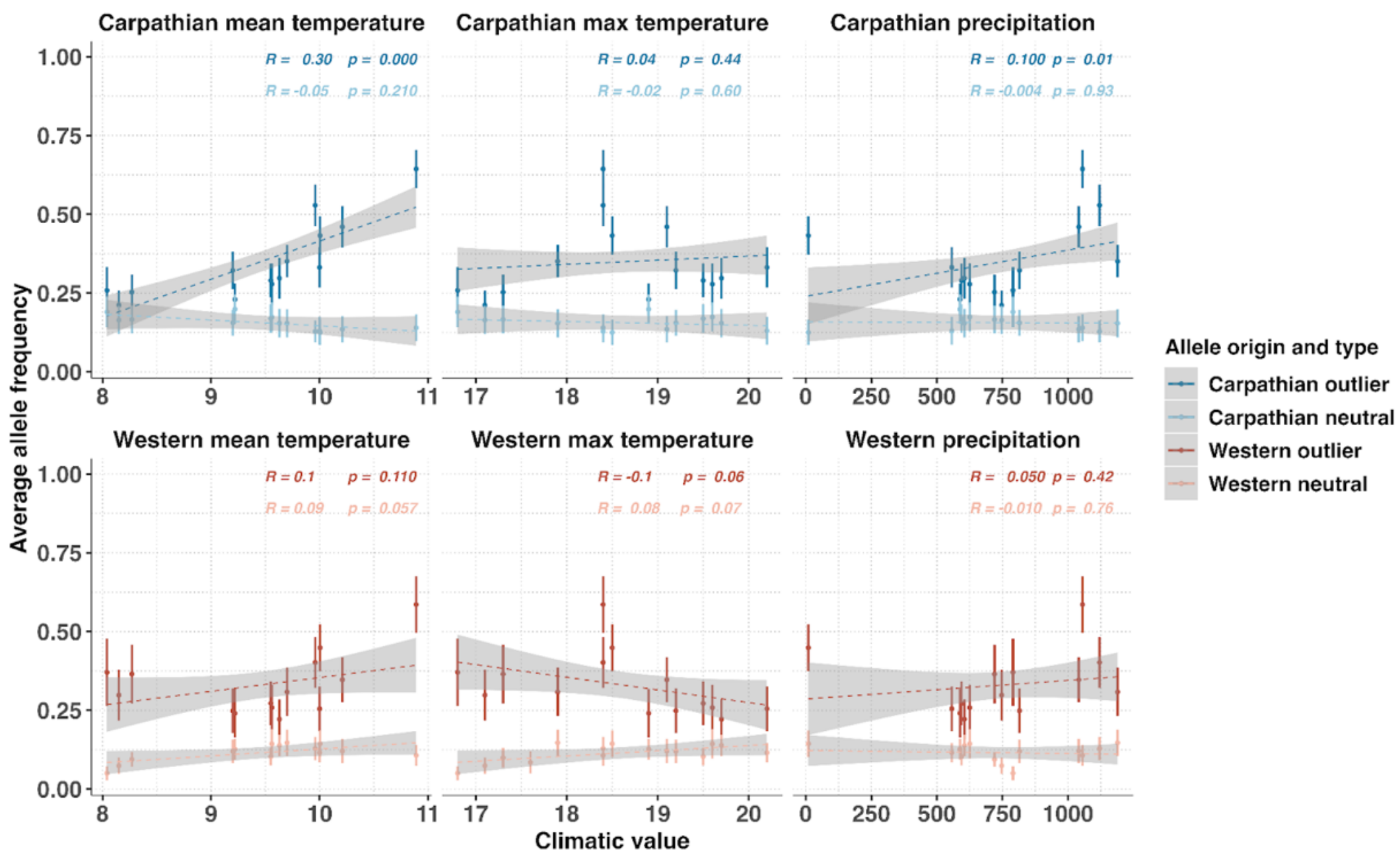

Prior submission – Fig 5

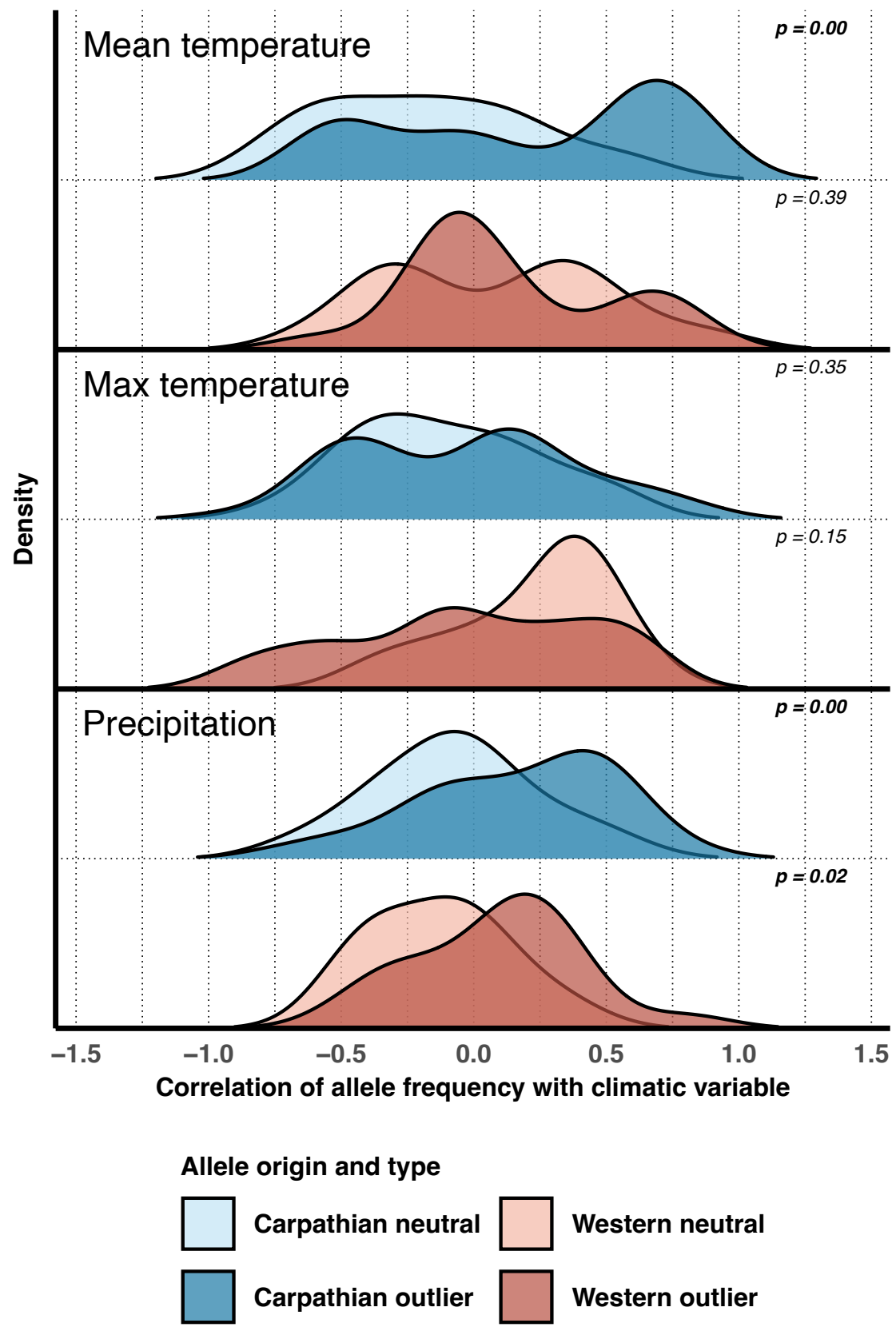

Revised dataset – Fig. 5

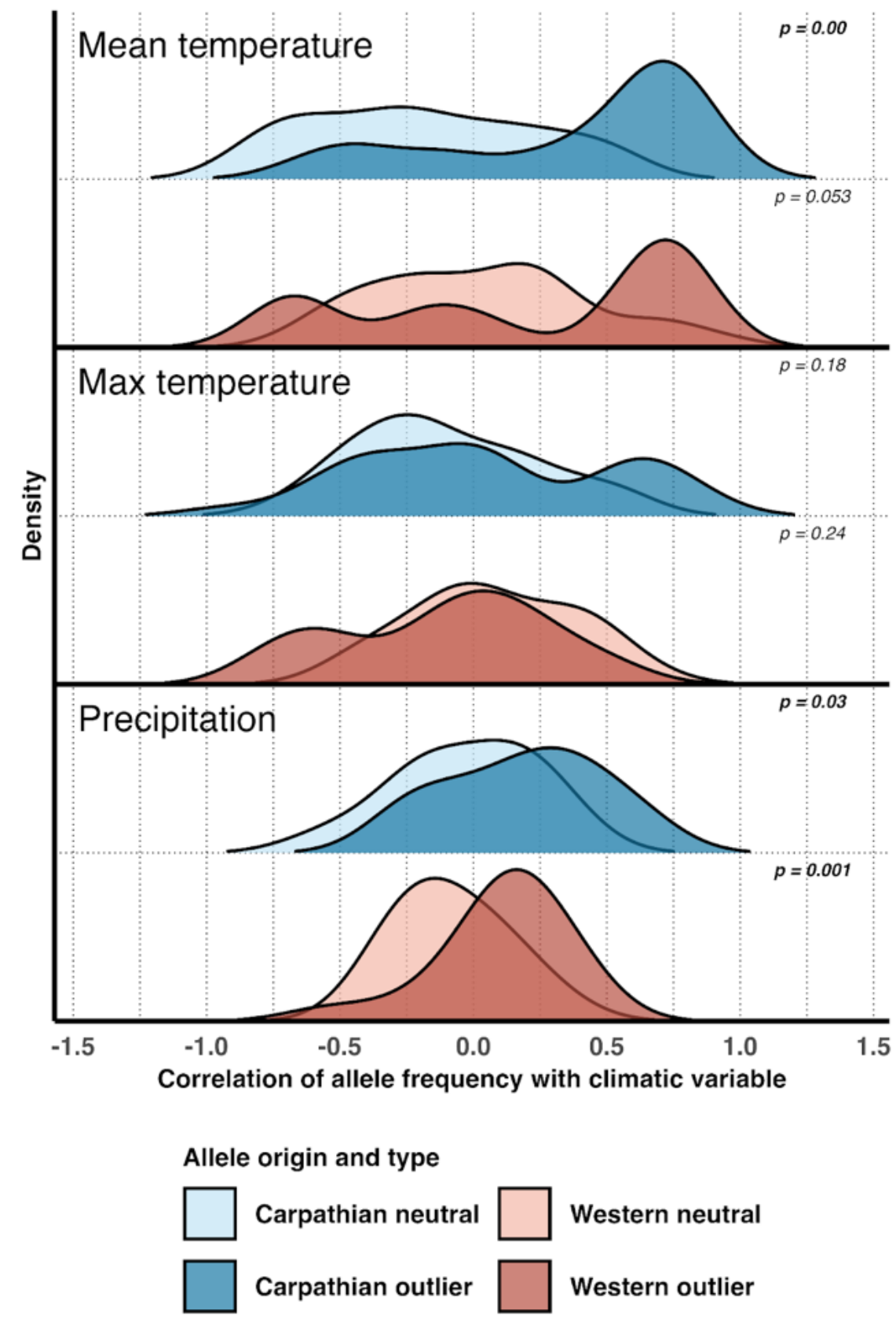

Prior submission – Supplementary Table 2

	Neutral	Outlier	Outlier	Neutral
**Variant Origin**	**Allele distribution**		**Haplotype trees**	
Widespread	521 | 48.5%	801 | 74.5%	802 | 74.6%	457 | 42.5%
Carpathian	110 | 10.2%	89 | 8.2%	56–65 | 5.2–6%	89–121 | 8.2–11.2%
Western	53 | 4.9%	50 | 4.6%	22–24 | 2–2.2%	29–55 | 2.7–5.1%
Britain	390 | 36.3%	131 | 12.1%	25–40 | 2.3–3.7%	314 | 29.2%
Unclear	*|*	*|*	143 | 13.3%	127 | 11.8%

Revised dataset – Supplementary Table 2

	Neutral	Outlier	Outlier	Neutral
**Variant Origin**	**Allele distribution**		**Haplotype trees**	
Widespread	466 | 43.3%	764 | 71.1%	730 | 68.0%	408 | 38.0%
Carpathian	118 | 10.9%	94 | 8.8%	29–42 | 2.7–3.9%	41–65 | 3.8–6.1%
Western	84 | 7.8%	77 | 7.2%	17–42 | 1.6–3.9%	33–72 | 3.1–6.7%
Britain	406 | 37.8%	139 | 12.9%	22 | 2%	243 | 22.6%
Unclear	* | *	* | *	238 | 22.2%	286 | 26.6%

Prior submission – Supplementary Figure 1

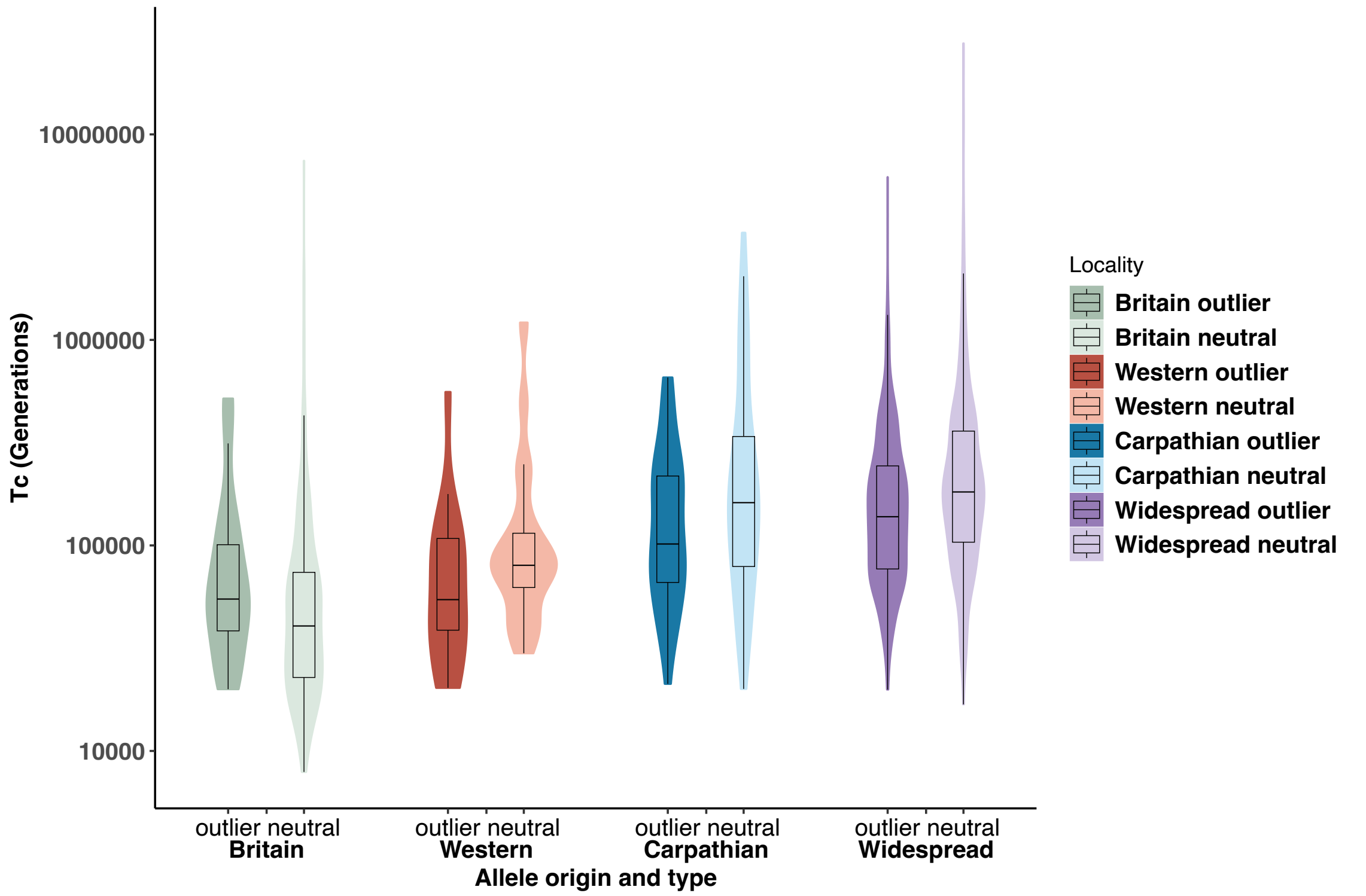

Revised dataset – Supplementary Figure 1

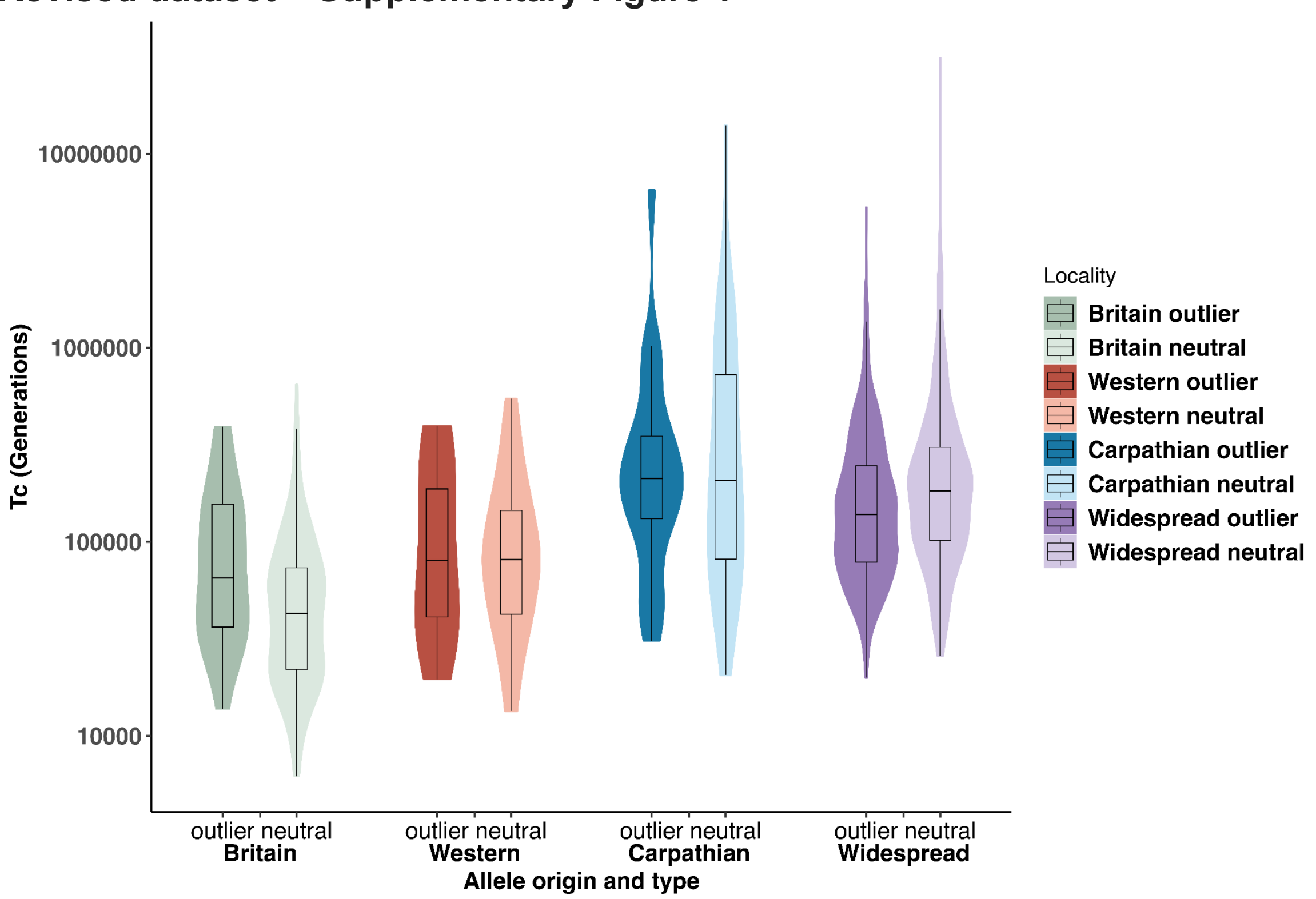

Prior submission – Supplementary Figure 2

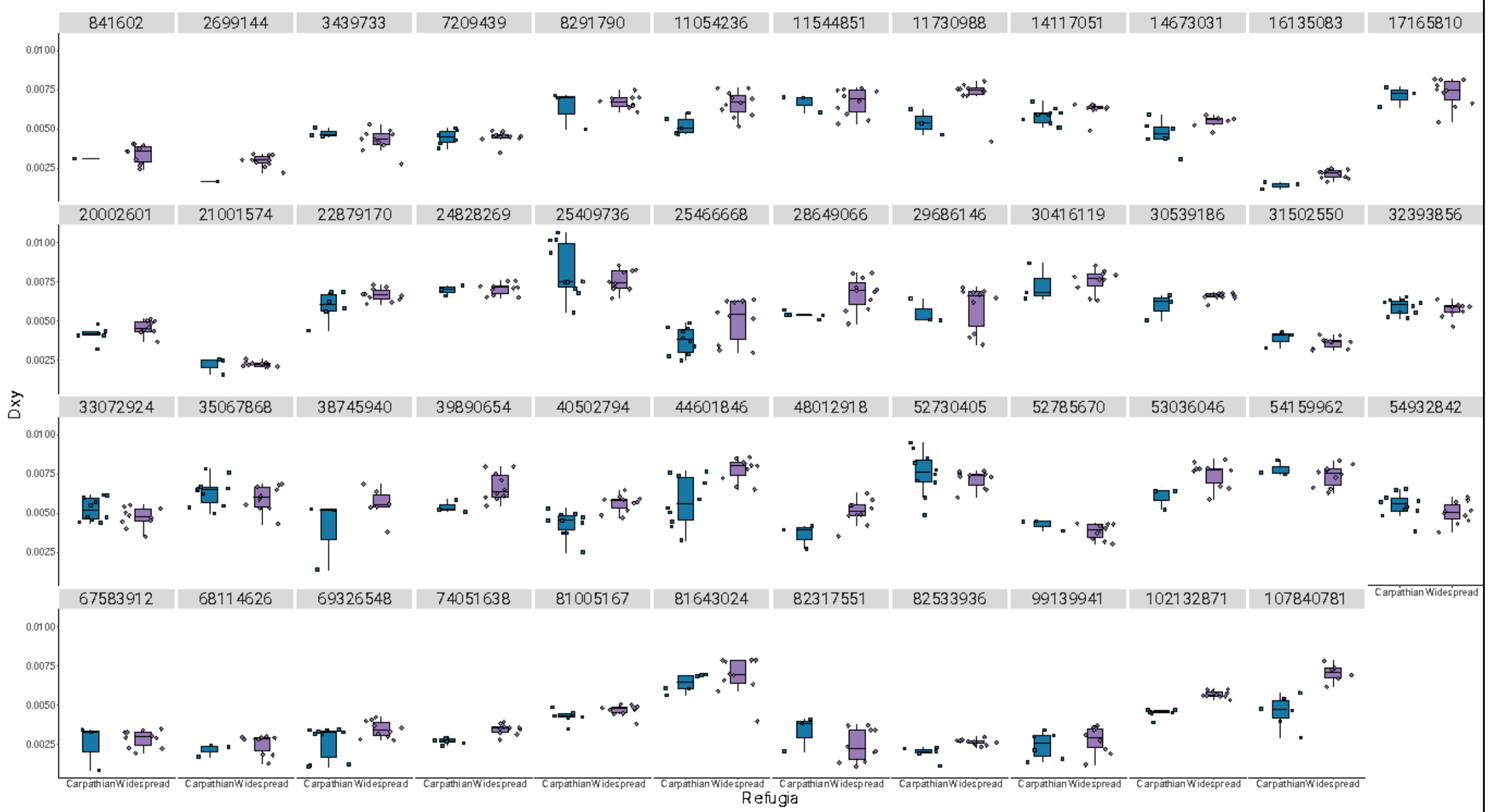

Revised dataset – Supplementary Figure 2

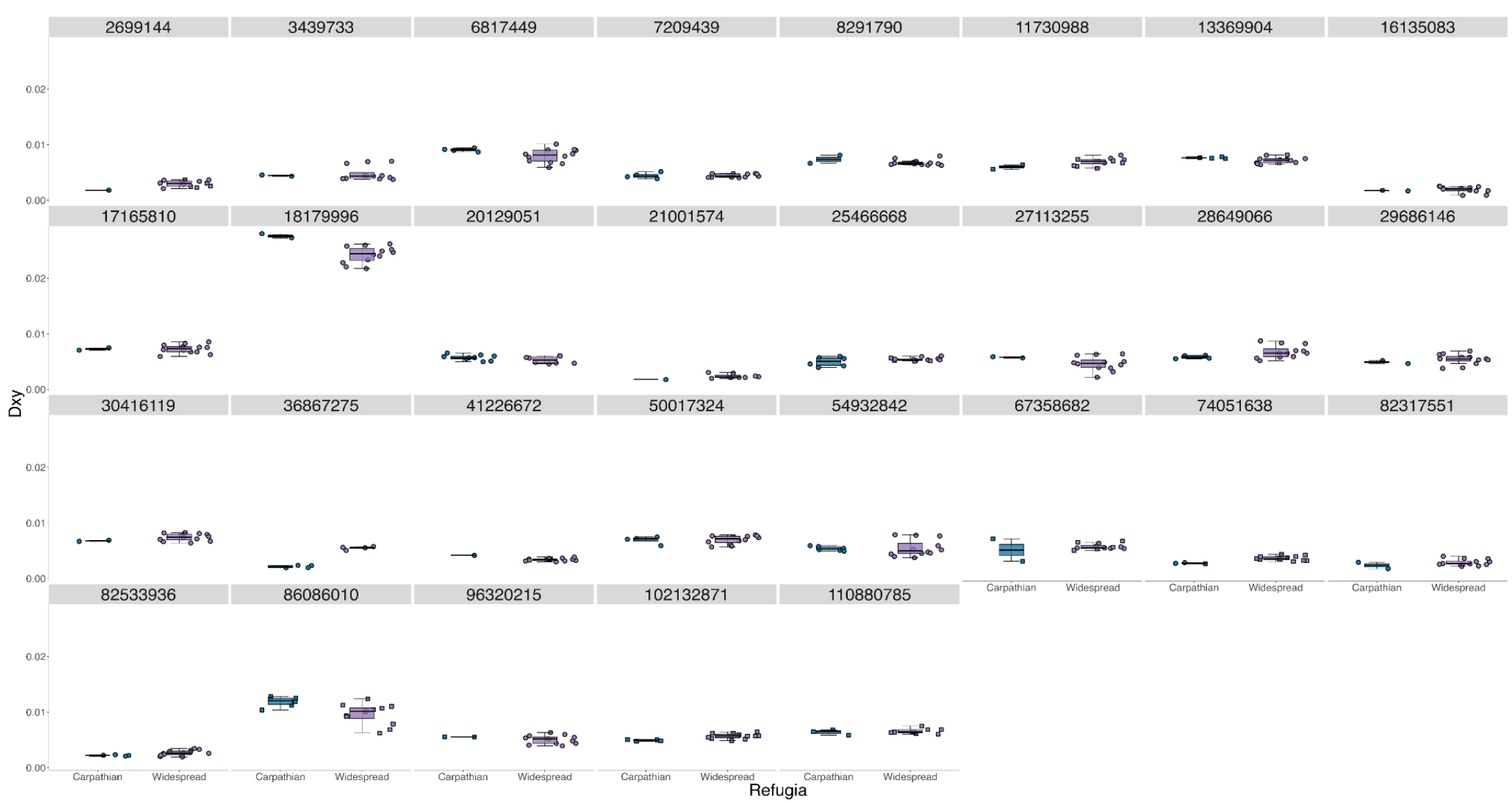

Prior submission – Supplementary Figure 3

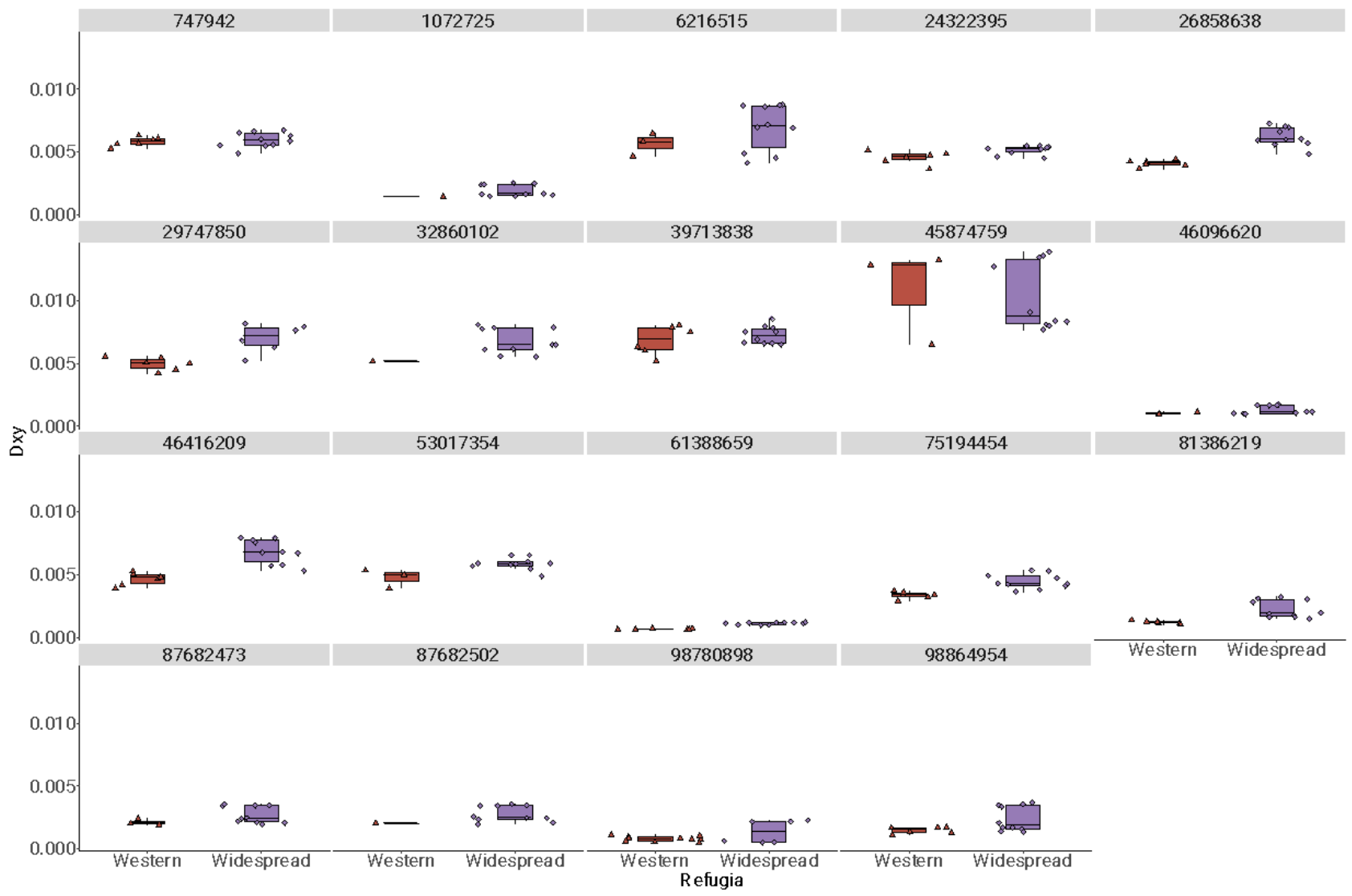

Revised dataset – Supplementary Figure 3

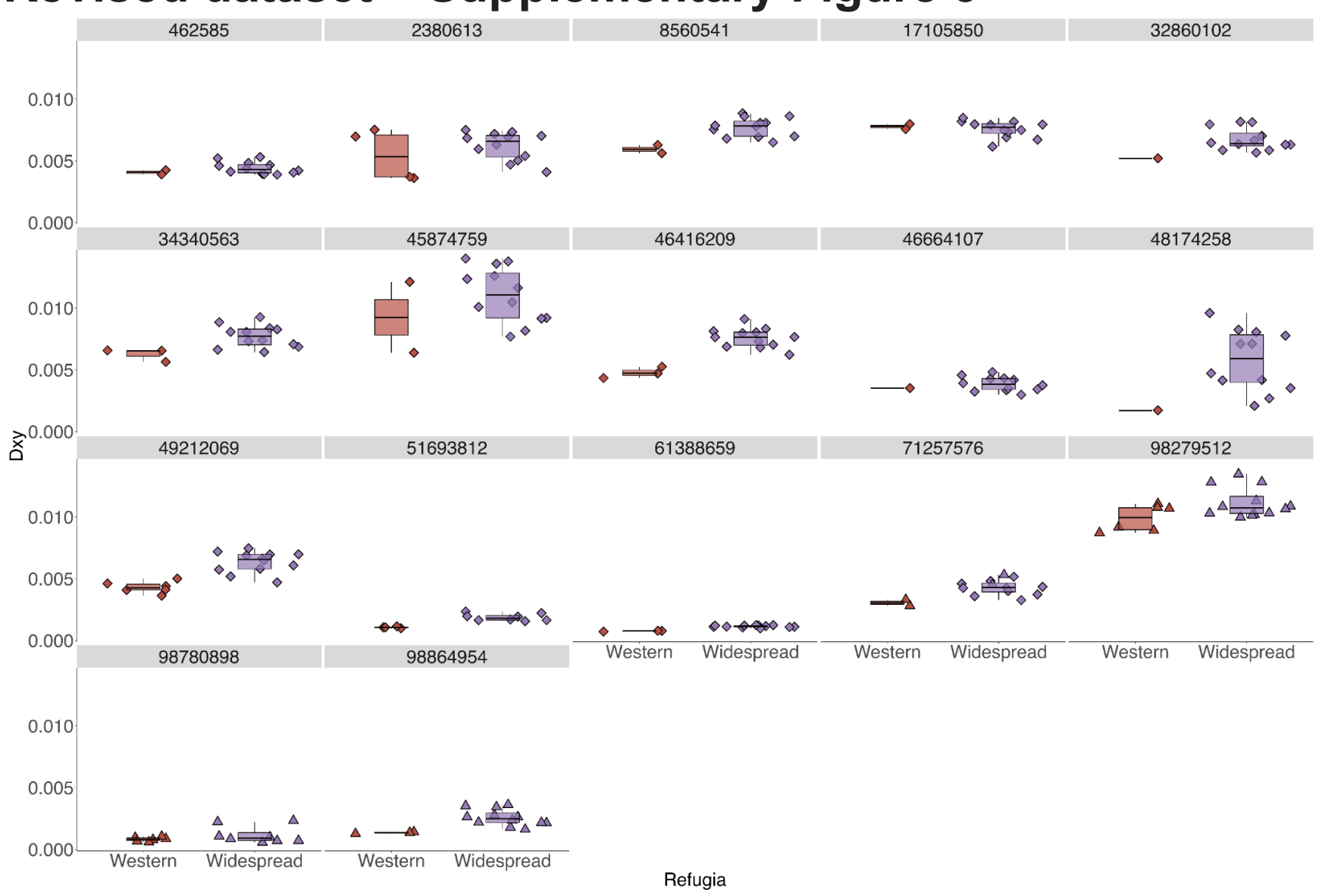

Prior submission – Supplementary Figure 4

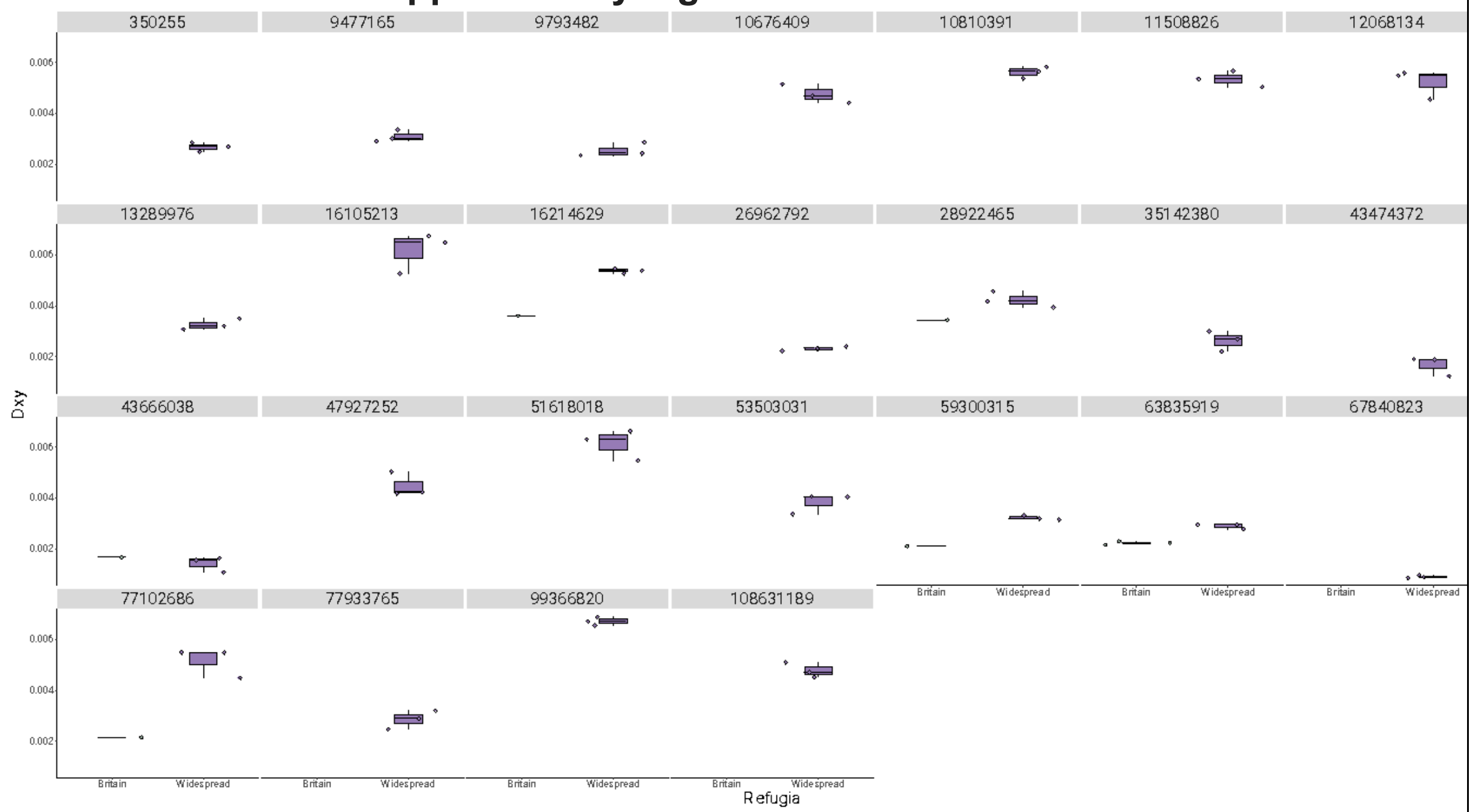

Revised dataset – Supplementary Figure 4

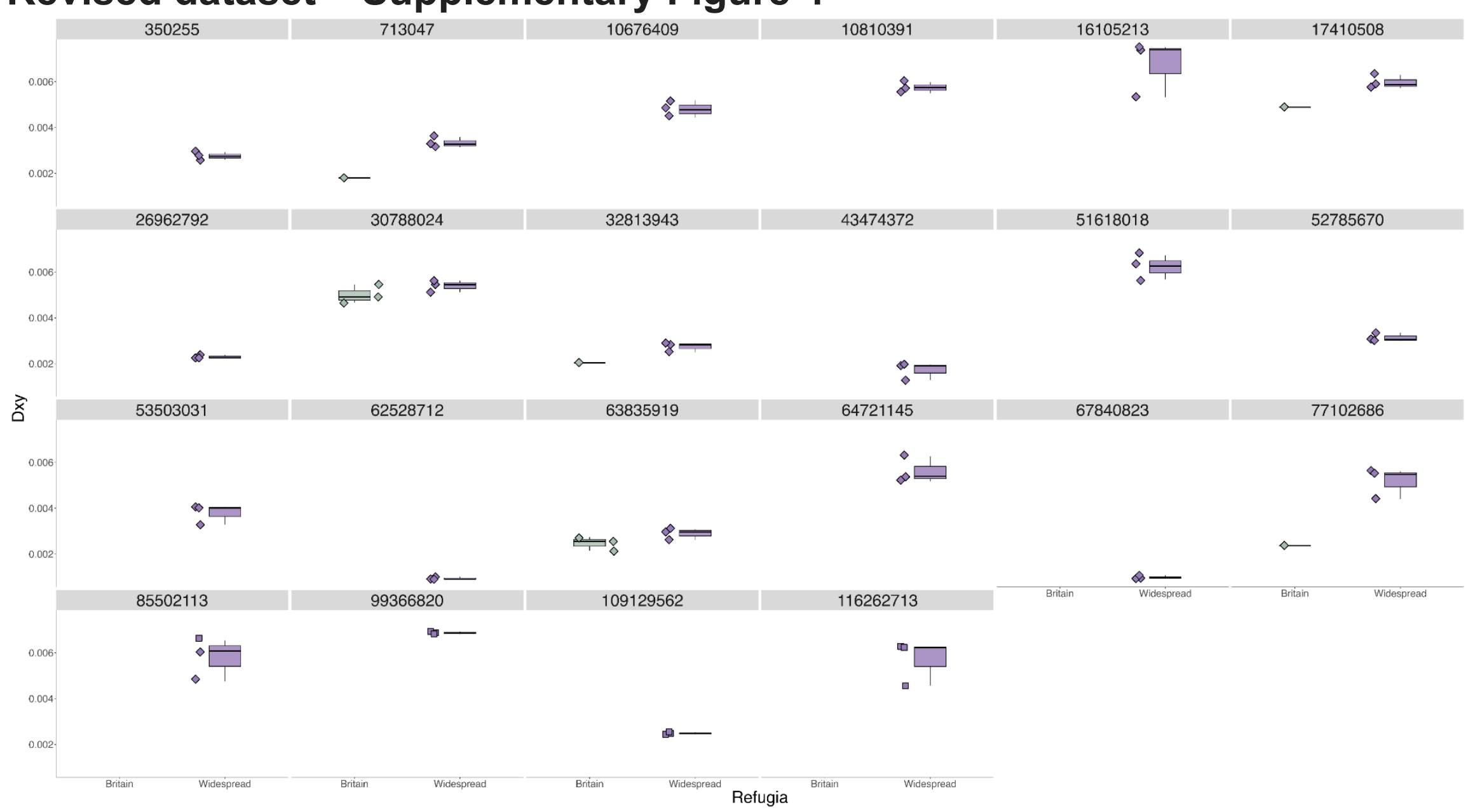

Prior submission – Supplementary Figure 5

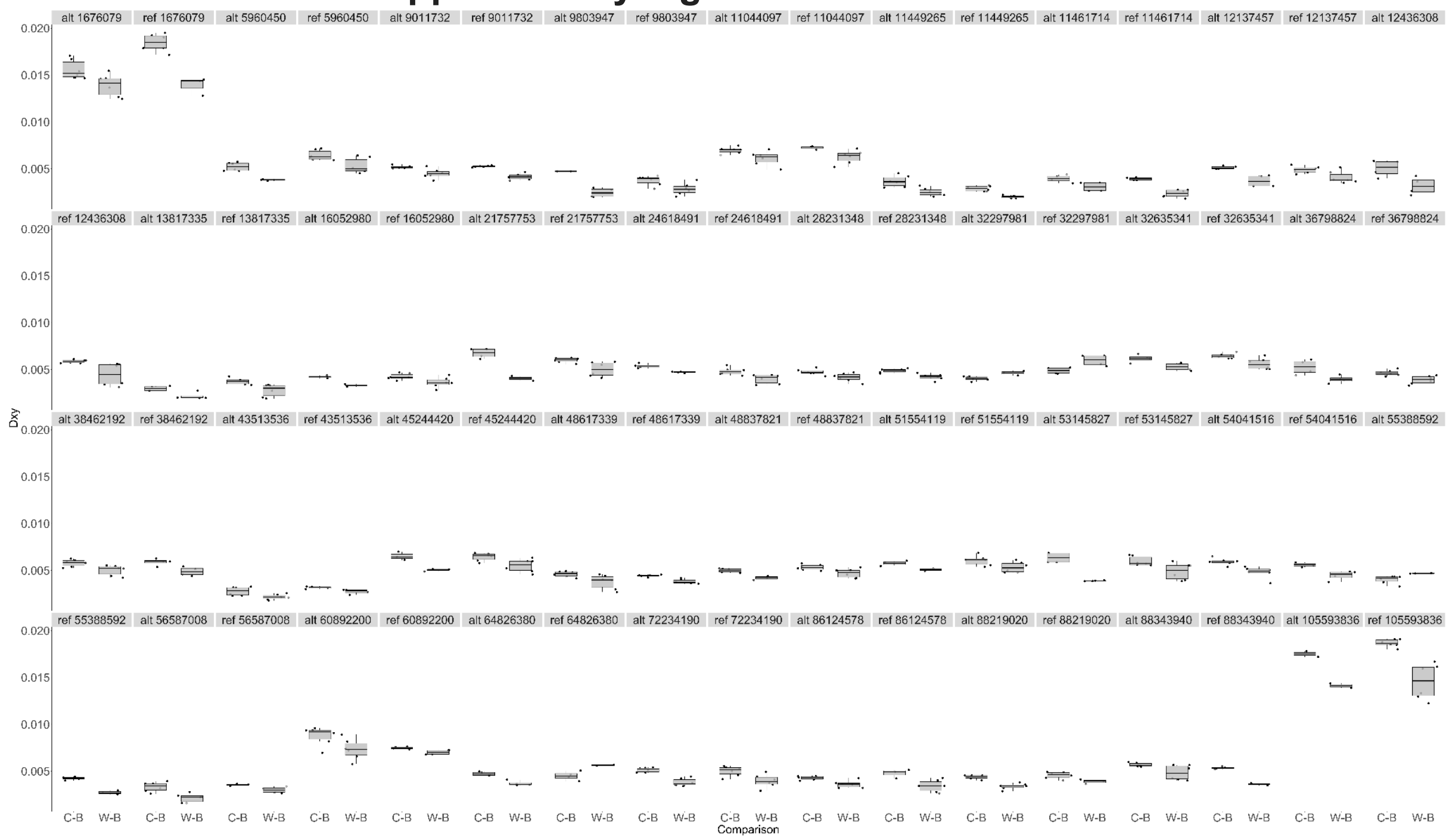

Revised dataset – Supplementary Figure 5

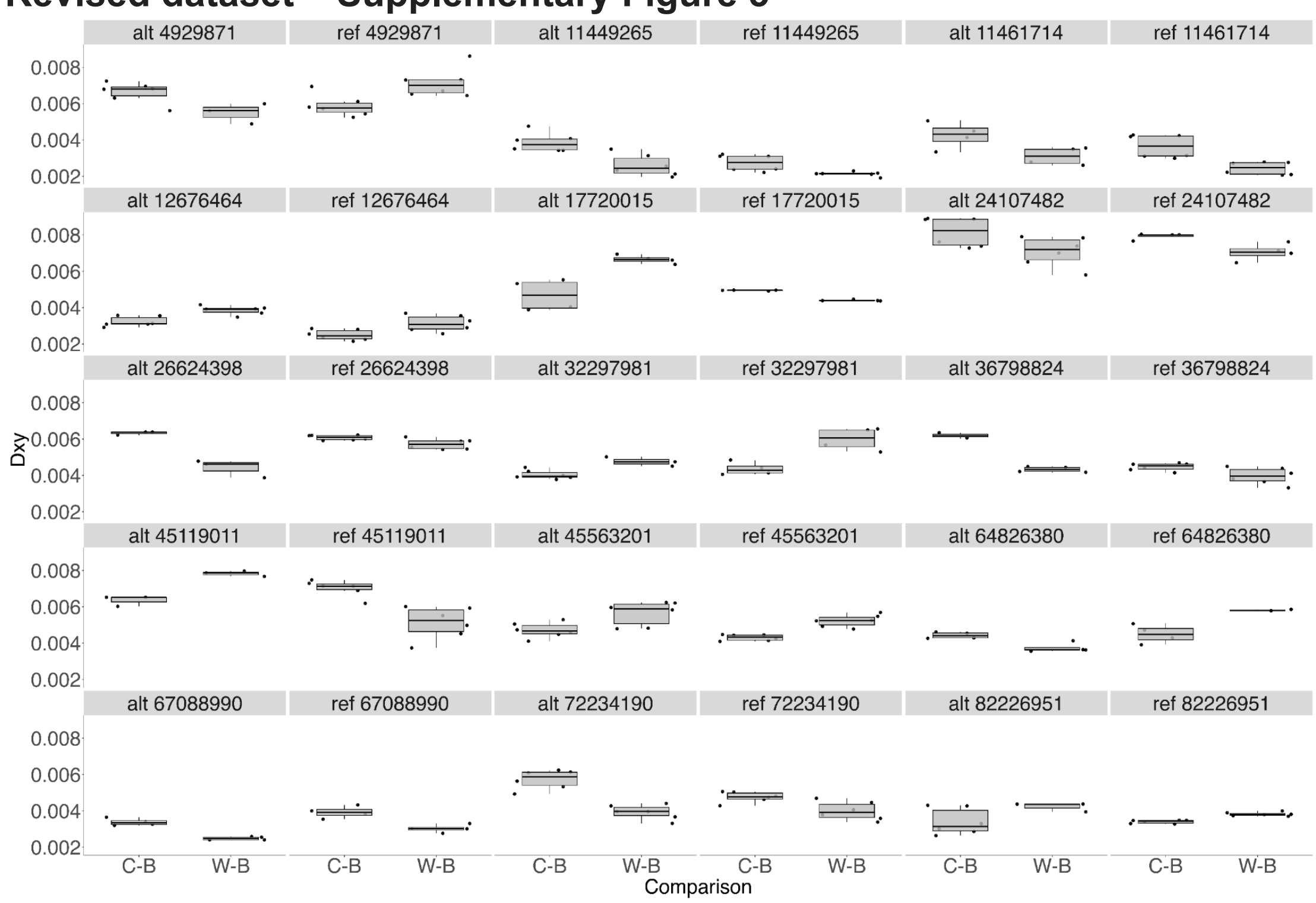

Prior submission – Supplementary Figure 6

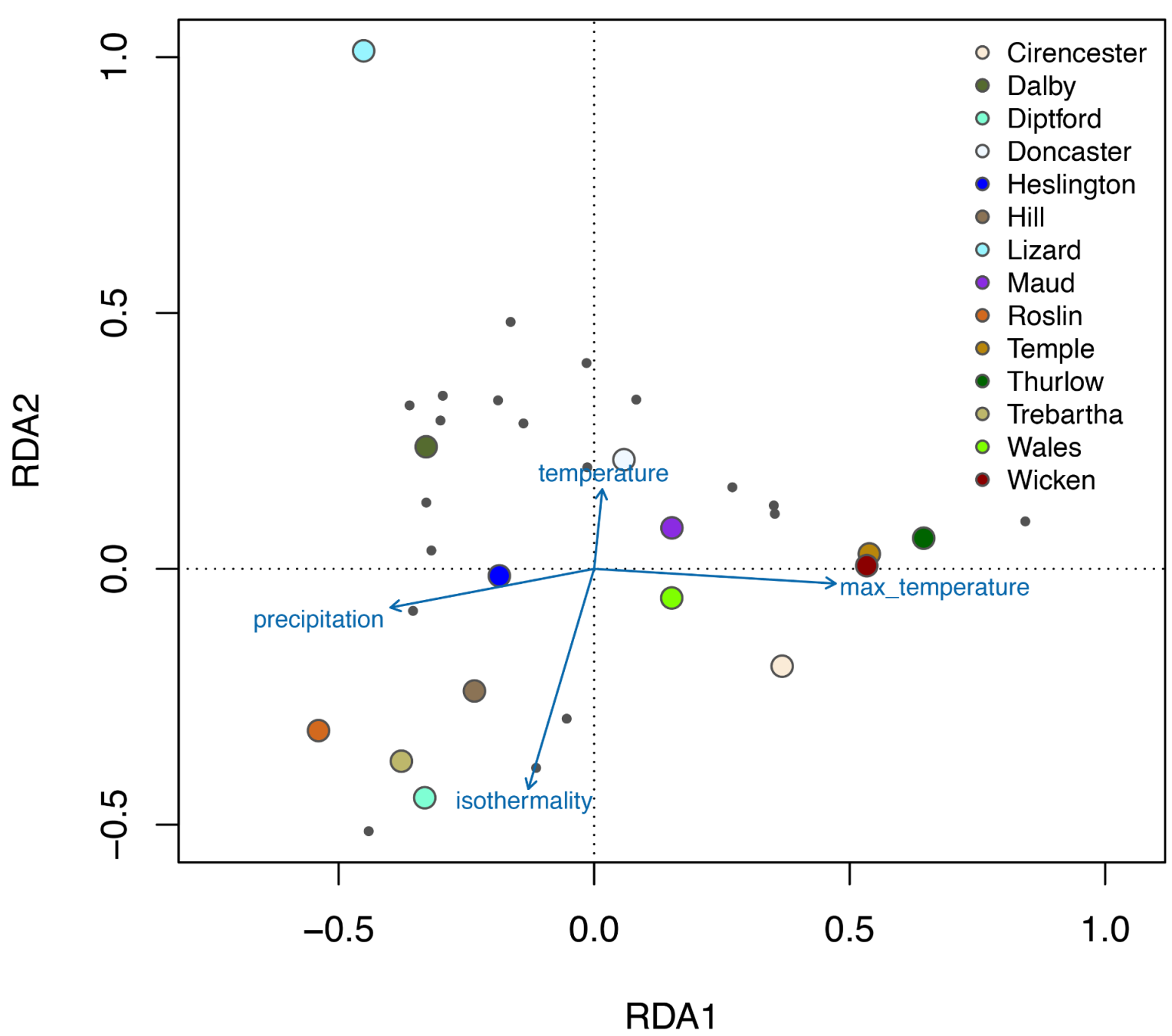

Revised dataset – Supplementary Figure 10 (figure numbers in this version changed to reflect order of reference in manuscript)

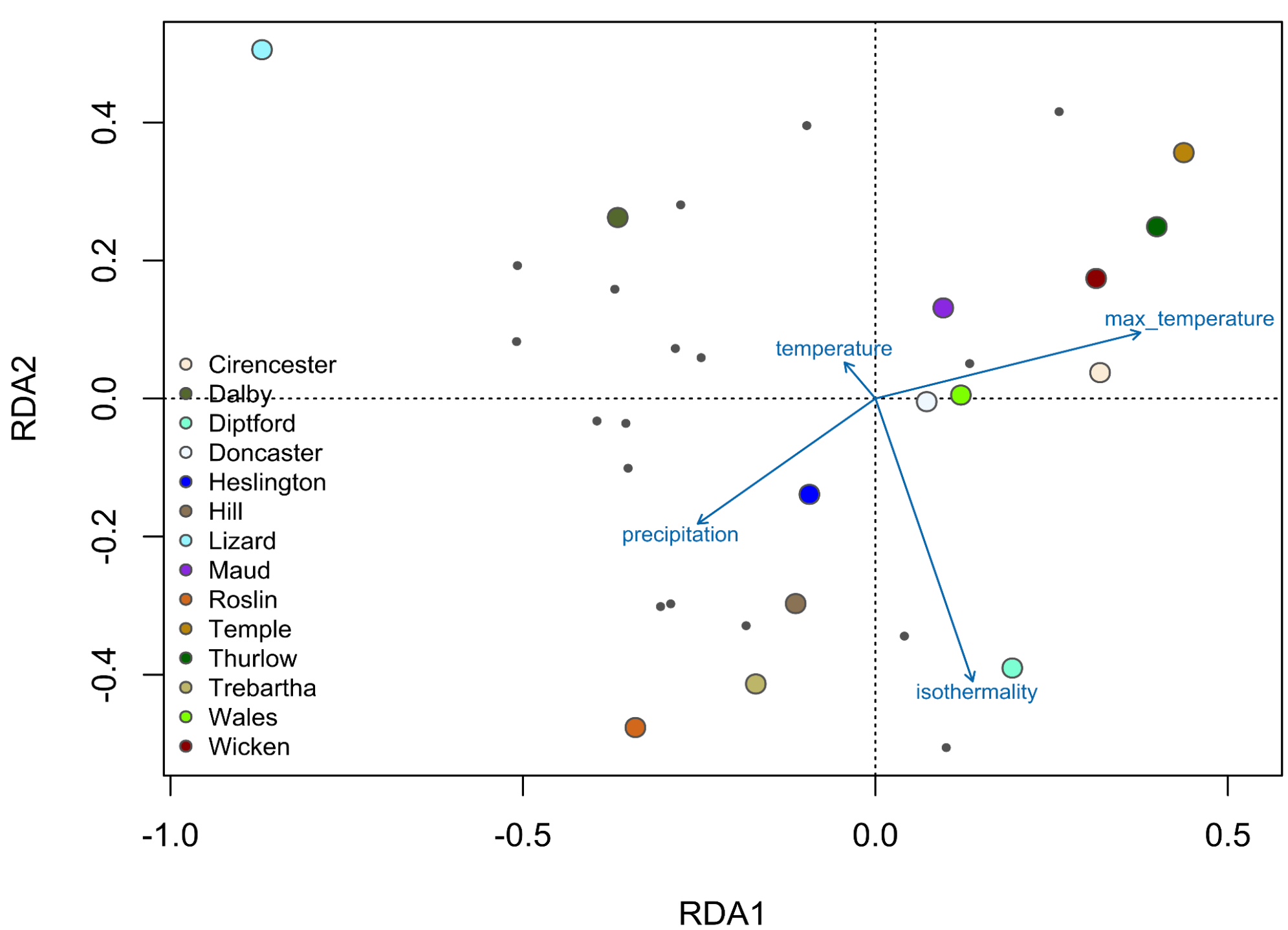

Prior submission – Supplementary Figure 7

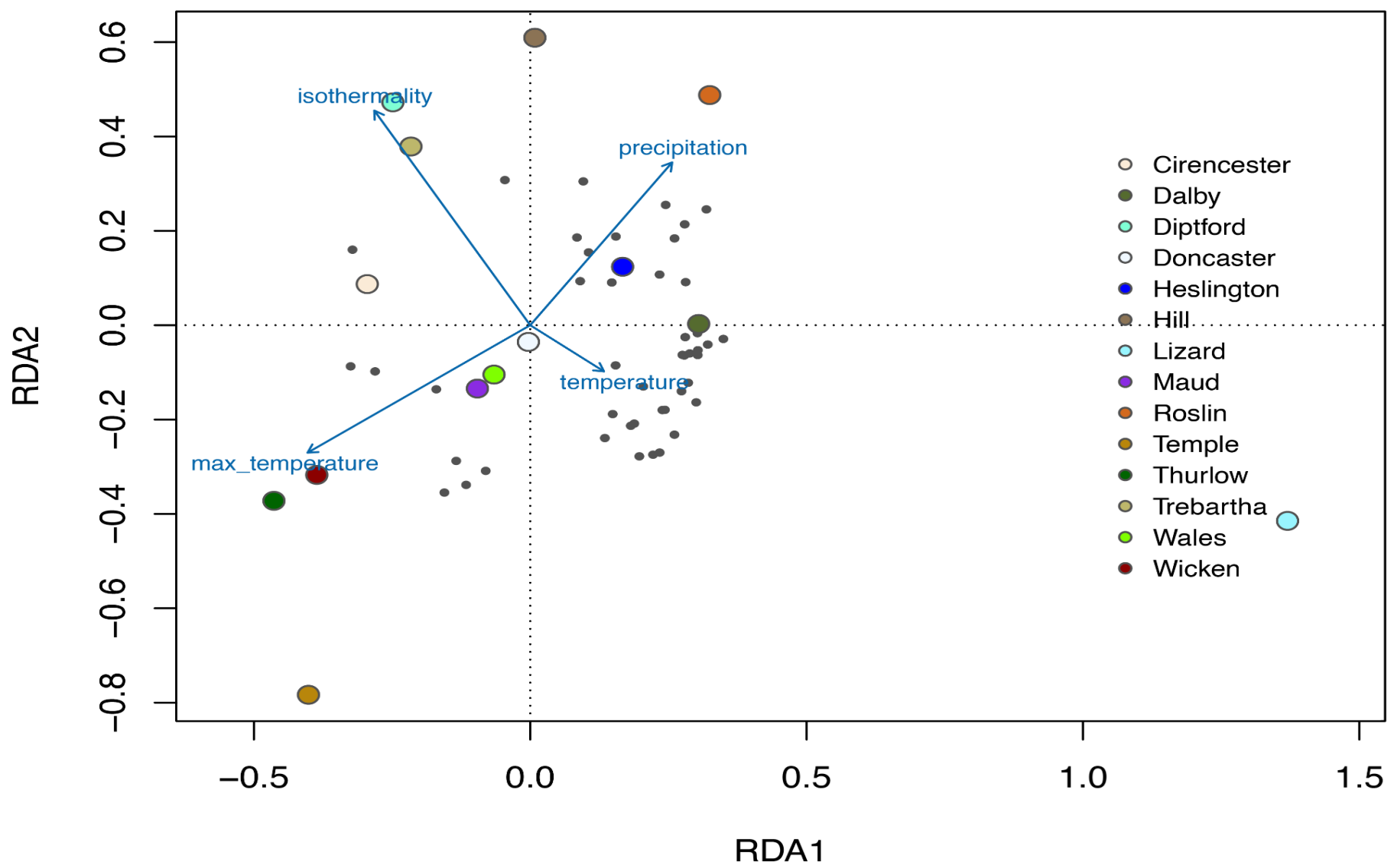

Revised dataset – Supplementary Figure 9 (renumbered from prior submission)

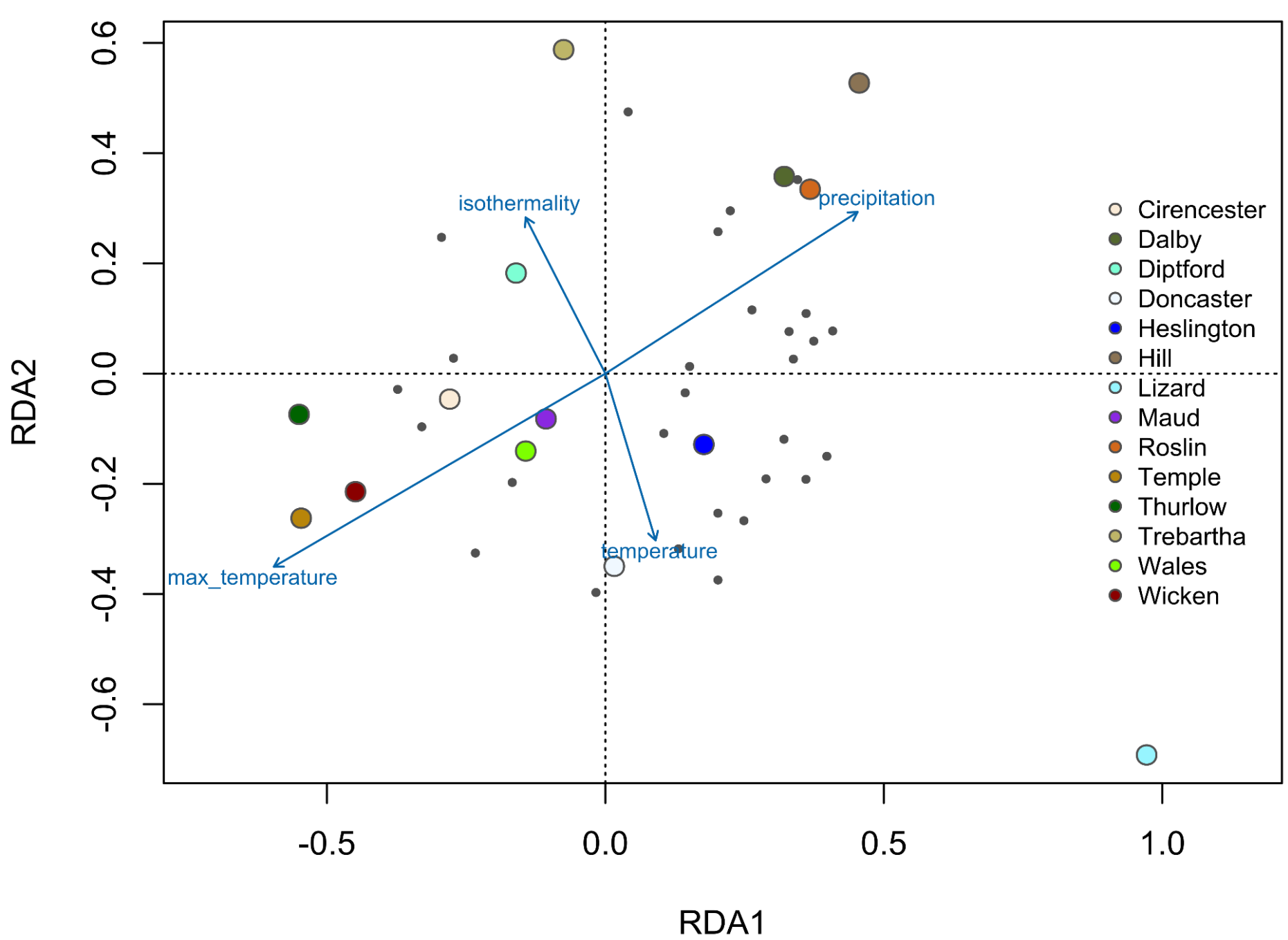

Prior submission – Supplementary Figure 8

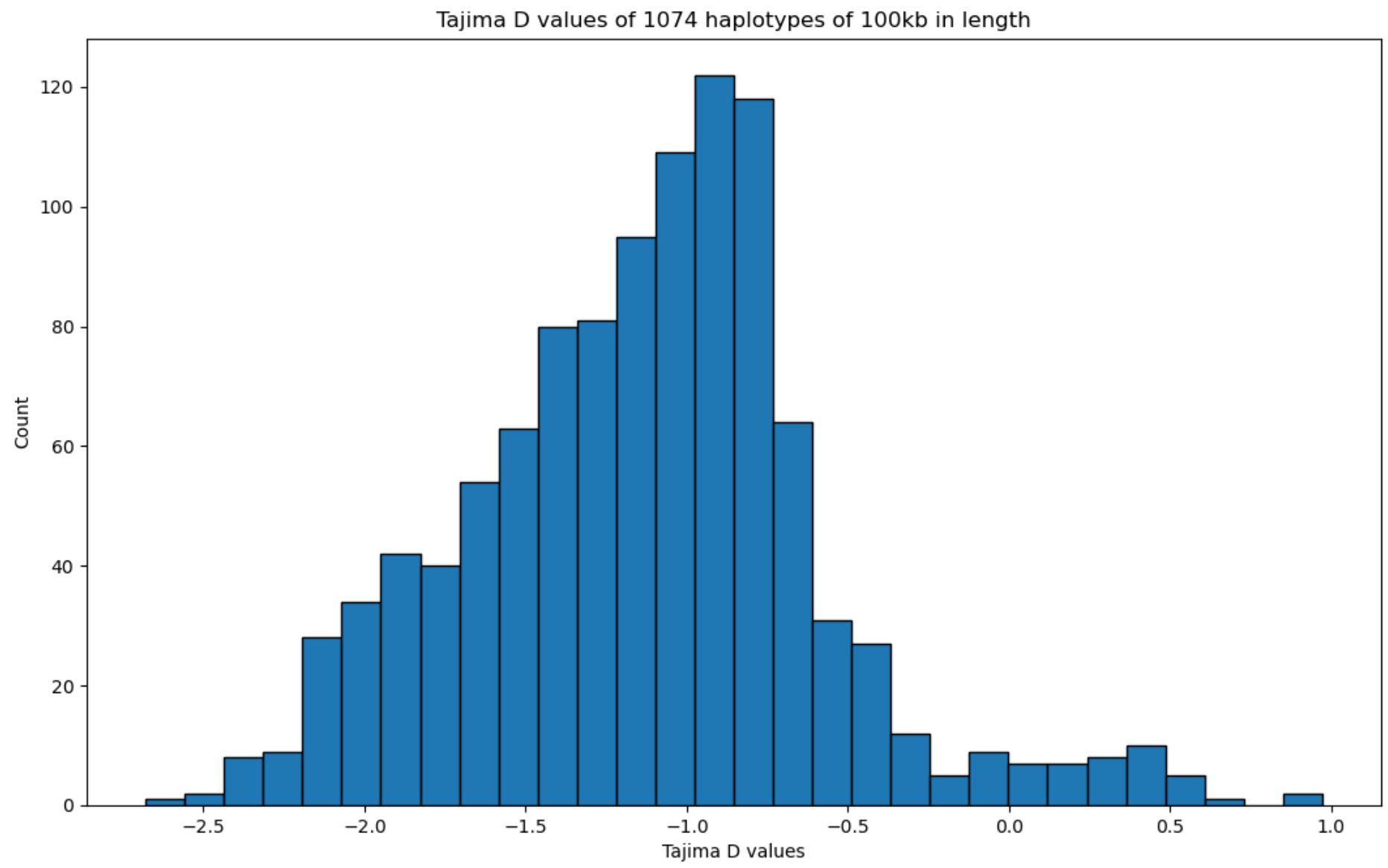

Revised dataset – Supplementary Figure 6 (renumbered from prior submission)

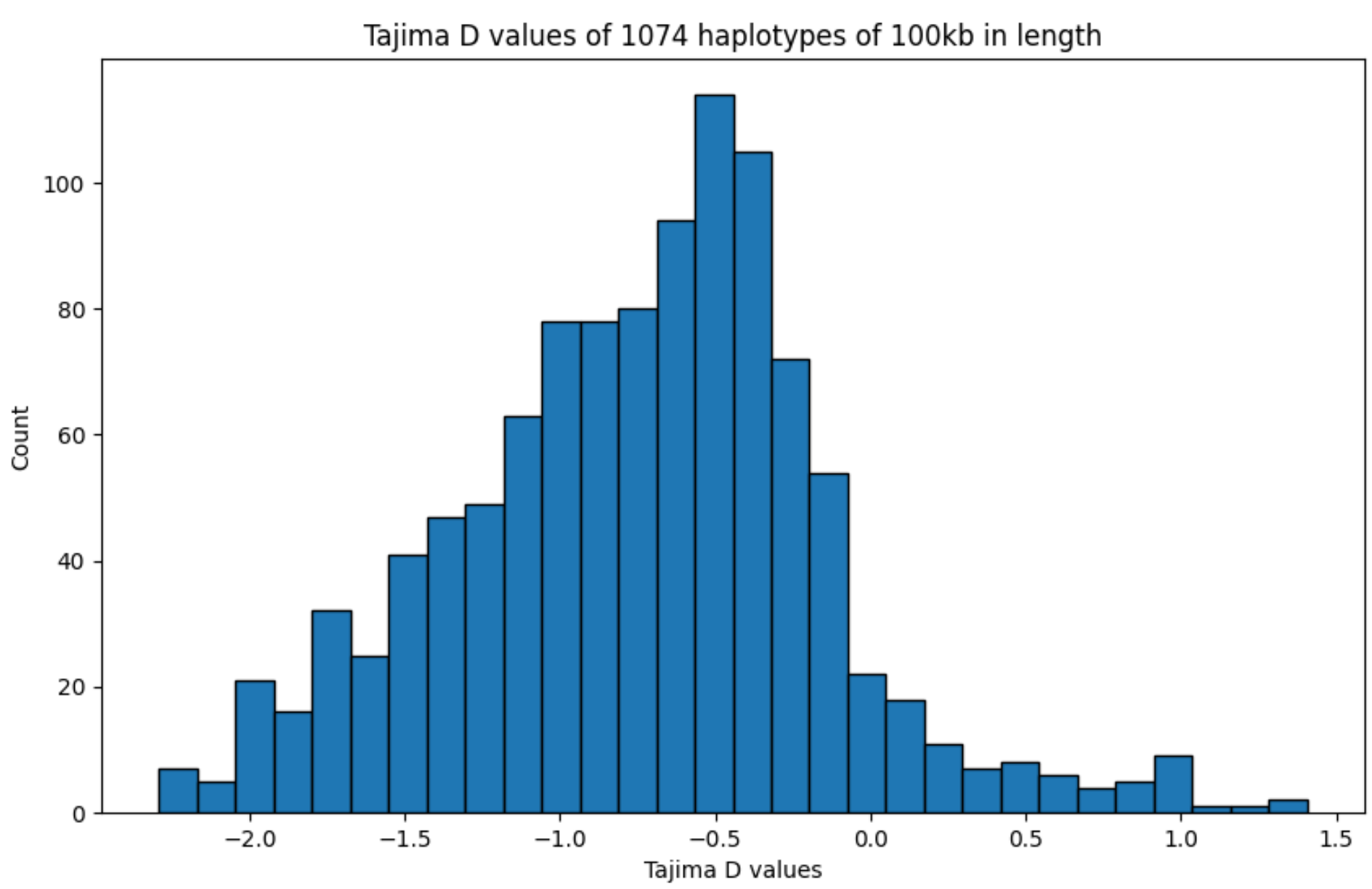

Prior submission – Supplementary Figure 9

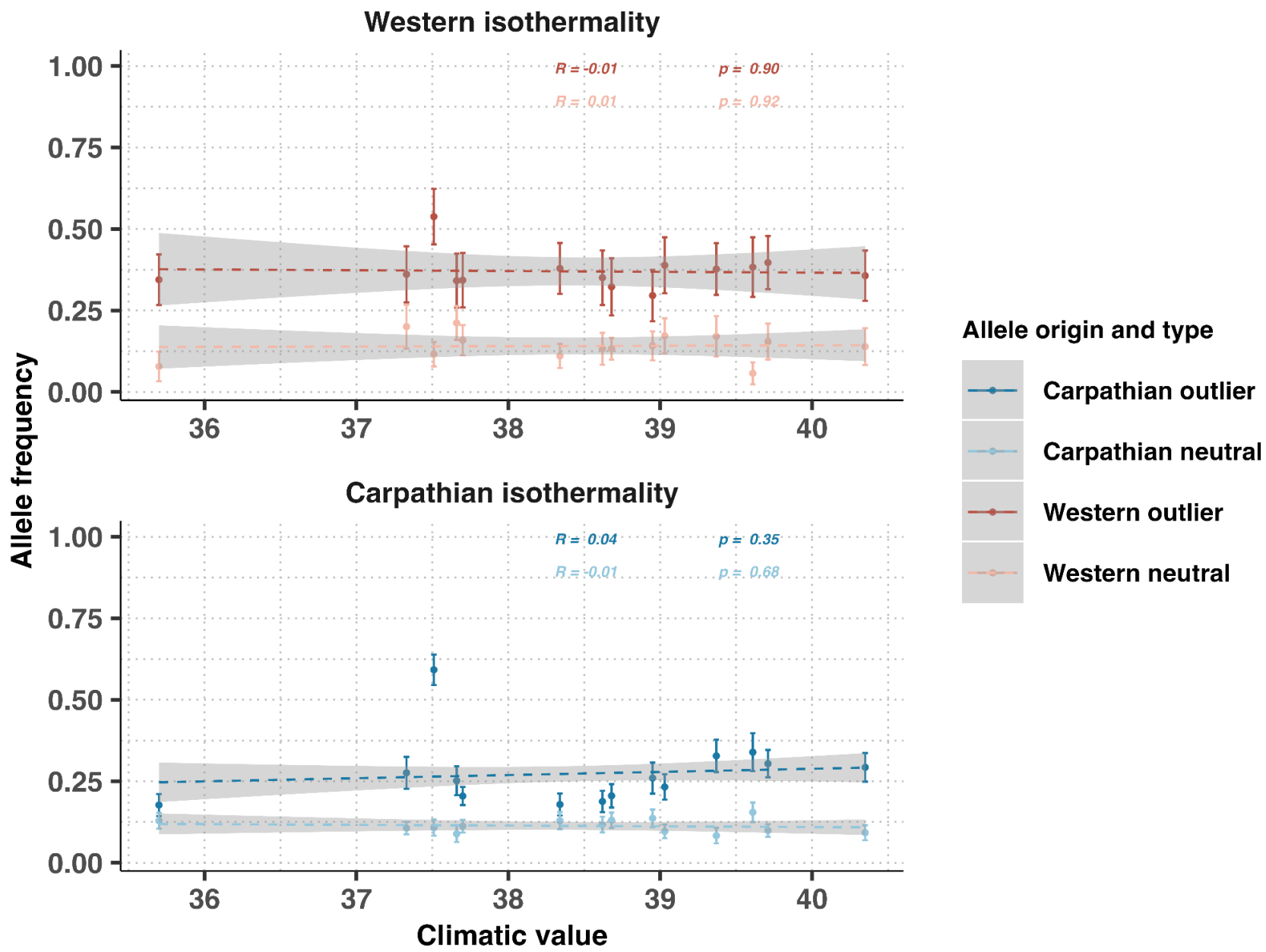

Revised dataset – Supplementary Figure 7 (renumbered from prior submission)

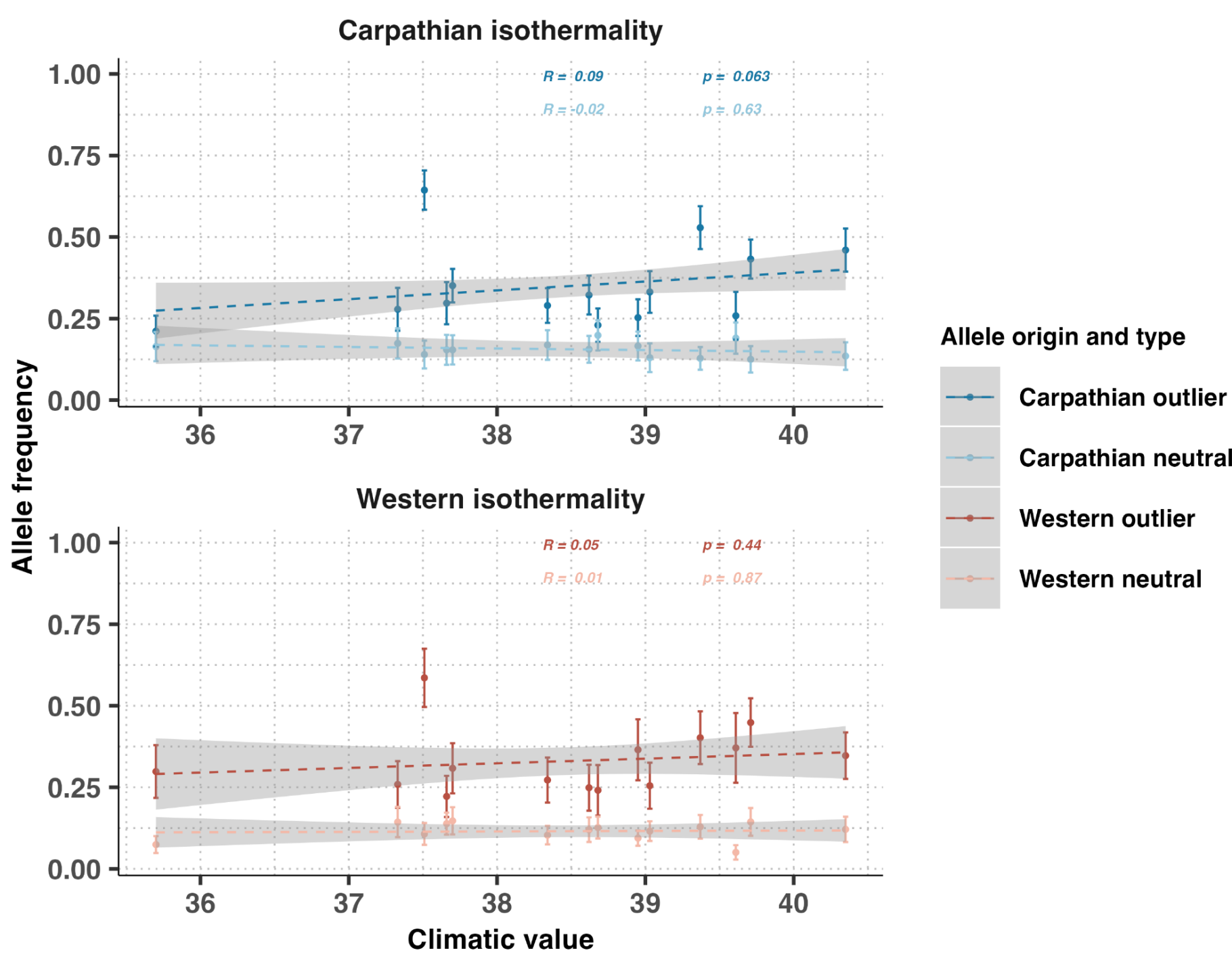

Prior submission – Supplementary Figure 10

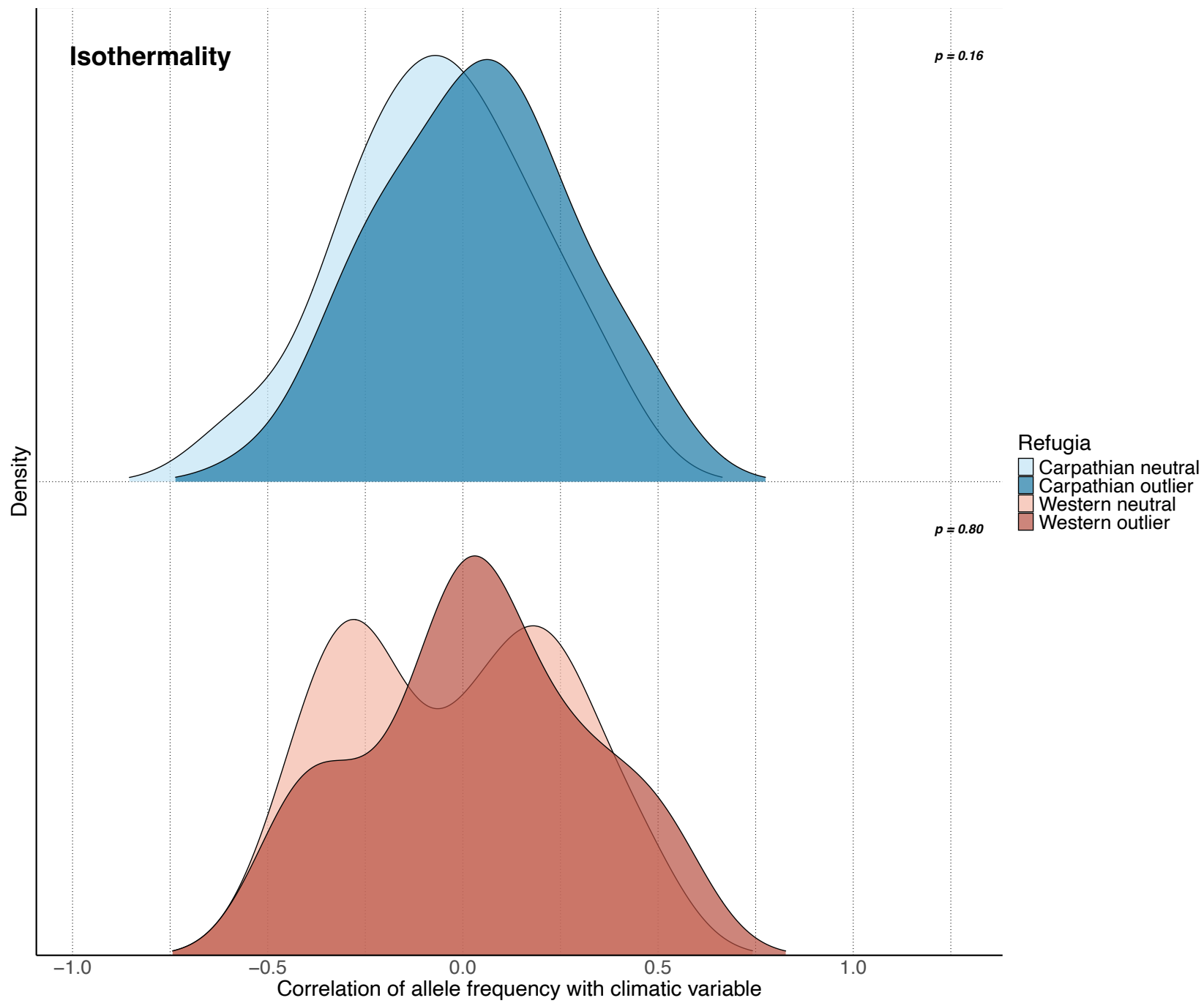

Revised dataset – Supplementary Figure S8 (renumbered from prior submission)

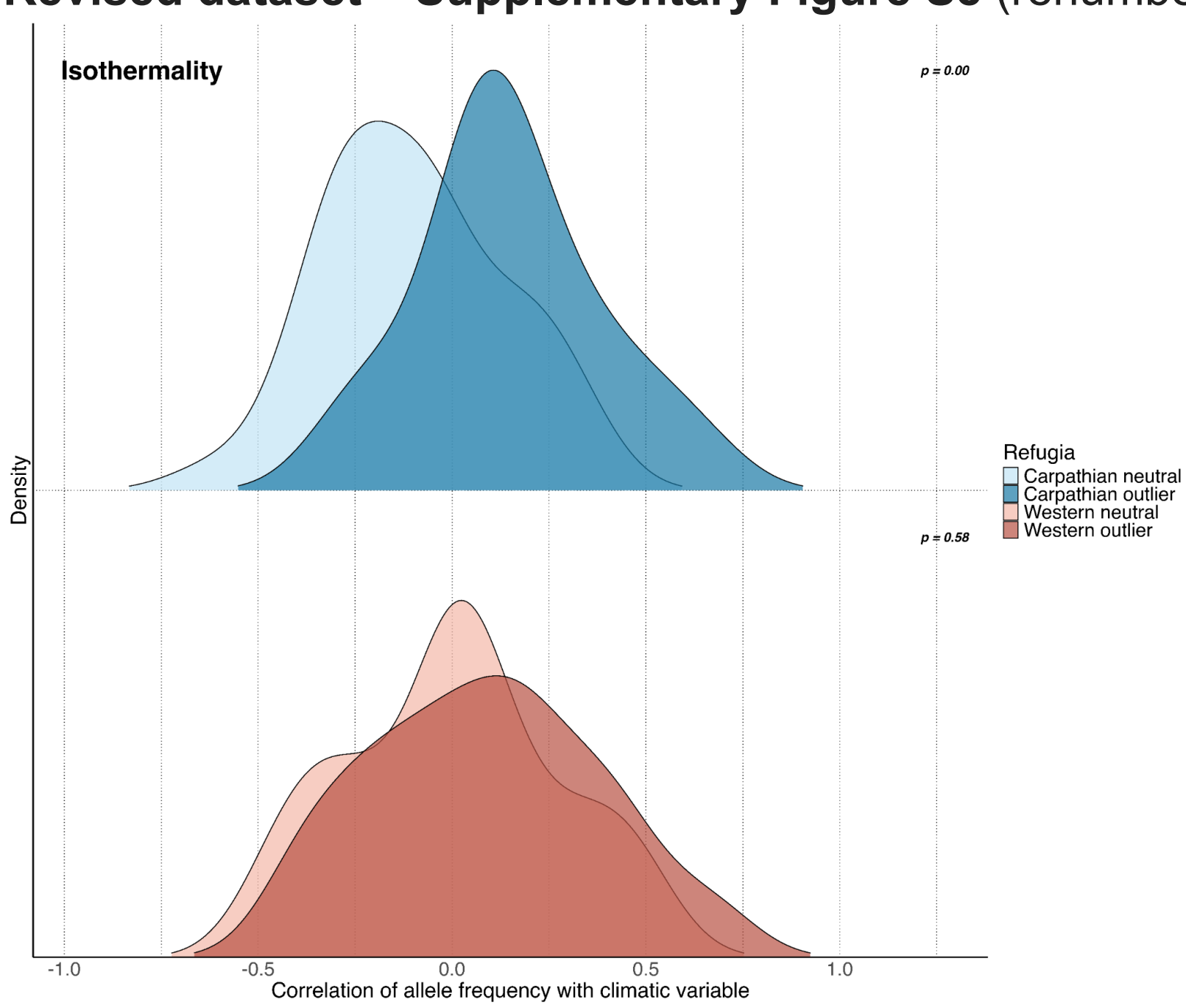

Supplement: Supplementary file 1 [file 42003_2026_10511_MOESM1_ESM.pdf]
