# Supplementary material for: Origins of adaptive genomic variation in a wild rodent
Source: Commun Biol. 2026 Jun 26;9:1360. doi: 10.1038/s42003-026-10511-6 (PMC13677672; doi:10.1038/s42003-026-10511-6)
Supplement: Supplementary file 2 [file 42003_2026_10511_MOESM2_ESM.pdf]

Supplementary Information

S1. Supplementary tables and results

(a) Estimation of divergence between haplotypes

The Dxy analyses verified that alleles inferred to originate from a refugium generally had similar or lower Dxy compared to widespread alleles at the same locus. For example, among 29 loci under selection containing Carpathian alleles, 19 had lower Dxy values than their widespread counterparts, while 10 had similar (i.e., not significantly higher) Dxy values (Figure S2). Of 17 loci containing Western alleles, 16 displayed lower Dxy values compared to widespread alleles, whereas 1 showed similar values (Figure S3). For British alleles, 6 of 22 loci had lower Dxy values than the corresponding widespread alleles, and the rest could not be evaluated (Figure S4) because haplotypes were not sufficiently widespread among populations.

(b) Correlation of allele frequencies with environmental variables

The pRDA analysis, which tested correlations between climatic variables and allele frequencies while controlling for genetic structure, revealed significant associations between mean annual temperature predictors and precipitation for Carpathian (Figure S9) and Western (Figure S10) alleles. Overall, Carpathian alleles were more strongly associated with mean annual temperature, while Western alleles were more negatively associated with the mean annual temperature and maximum temperature of the warmest month.

Supplementary Table S1: Number of samples for each population included in each analysis of allele origin (O), age (A), divergence analysis (D), or climate (C).

Location	Population	N_{inds}	Analyses	Latitude	Longitude
Britain	Cirencester	10	C	51.76	-4.16
Britain	Dalby	8	C	51.78	-1.69
Britain	Diptford	10	C	54.25	-0.69
Britain	Doncaster	10	C	50.41	-3.75
Britain	Heslington	10	OADC	53.49	-1.13
Britain	Hill	3	C	57.48	-2.08
Britain	Lizard	10	OADC	52.13	0.44
Britain	Maud	9	OADC	50.05	-5.25
Britain	Roslin	10	C	57.48	-2.08
Britain	Temple	5	C	52.13	0.41
Britain	Thurlow	7	C	53.93	-1.01
Britain	Trebartha	4	C	50.57	-4.44
Britain	Wales	8	C	55.85	-3.1
Britain	Wicken	6	C	52.28	0.27
Carpathian	Slovakia	10	OAD	48.84	18.66
Carpathian	Czechia	10	OAD	49.7	18.38
Western	France	10	OAD	48.92	0.2
Western	Netherlands	10	OAD	52.25	4.47
Calabria	Slia Grande	1	O	39.3	16.5

Supplementary Table S2: Counts (left of pipe) and percentages (right) of loci with one allele associated with each origin according to allele frequency and haplotype trees. Genealogical results are given as a range, with lower-end counts reflecting loci with variant origins associated with a single location based upon both allele frequency and haplotype analyses, whereas upper estimates include loci coded as maintained (widespread based on the allele distribution analyses but with a variant that appears to have only entered Britain via a single refugium based upon haplotype trees and Dxy results). Alleles that are not widespread in distribution (i.e., were assigned a possible refugial or British origin based on allele frequency) but lack a definitive single origin in haplotype trees were classified as unclear.

	**Neutral**	**Outlier**	**Outlier**	**Neutral**
**Variant Origin**	**Allele distribution**		**Haplotype trees**	
Widespread	466 | 43.3%	764 | 71.1%	730 | 68.0%	408 | 38.0%
Carpathian	118 | 10.9%	94 | 8.8%	29–42 | 2.7–3.9%	41–65 | 3.8–6.1%
Western	84 | 7.8%	77 | 7.2%	17–42 | 1.6–3.9%	33–72 | 3.1–6.7%
Britain	406 | 37.8%	139 | 12.9%	22 | 2%	243 | 22.6%
Unclear	* | *	* | *	238 | 22.2%	286 | 26.6%

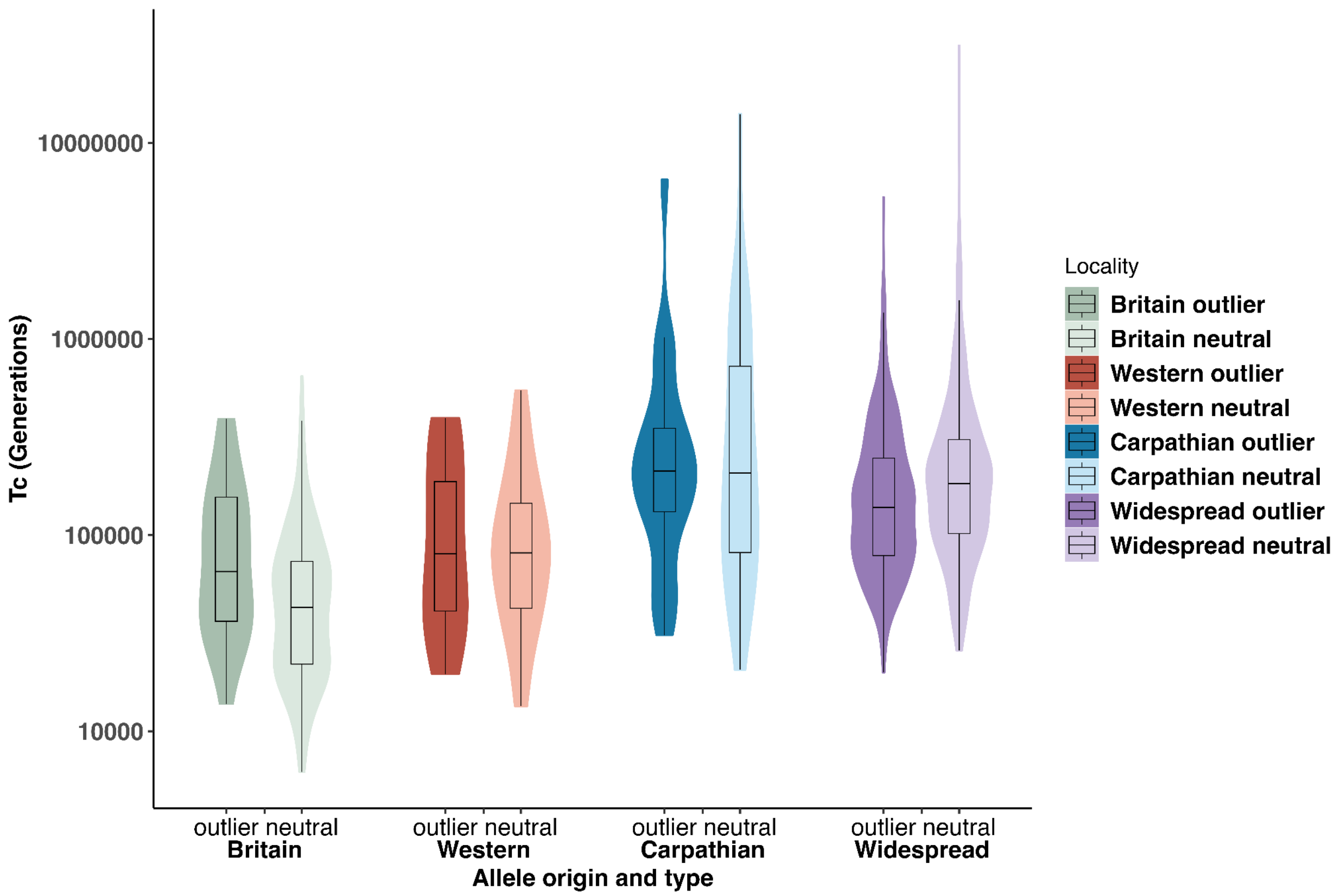

Supplementary Figure 1: Time of coalescence (T_c) in generations corresponding to outlier and neutral alleles relative to their inferred origins.

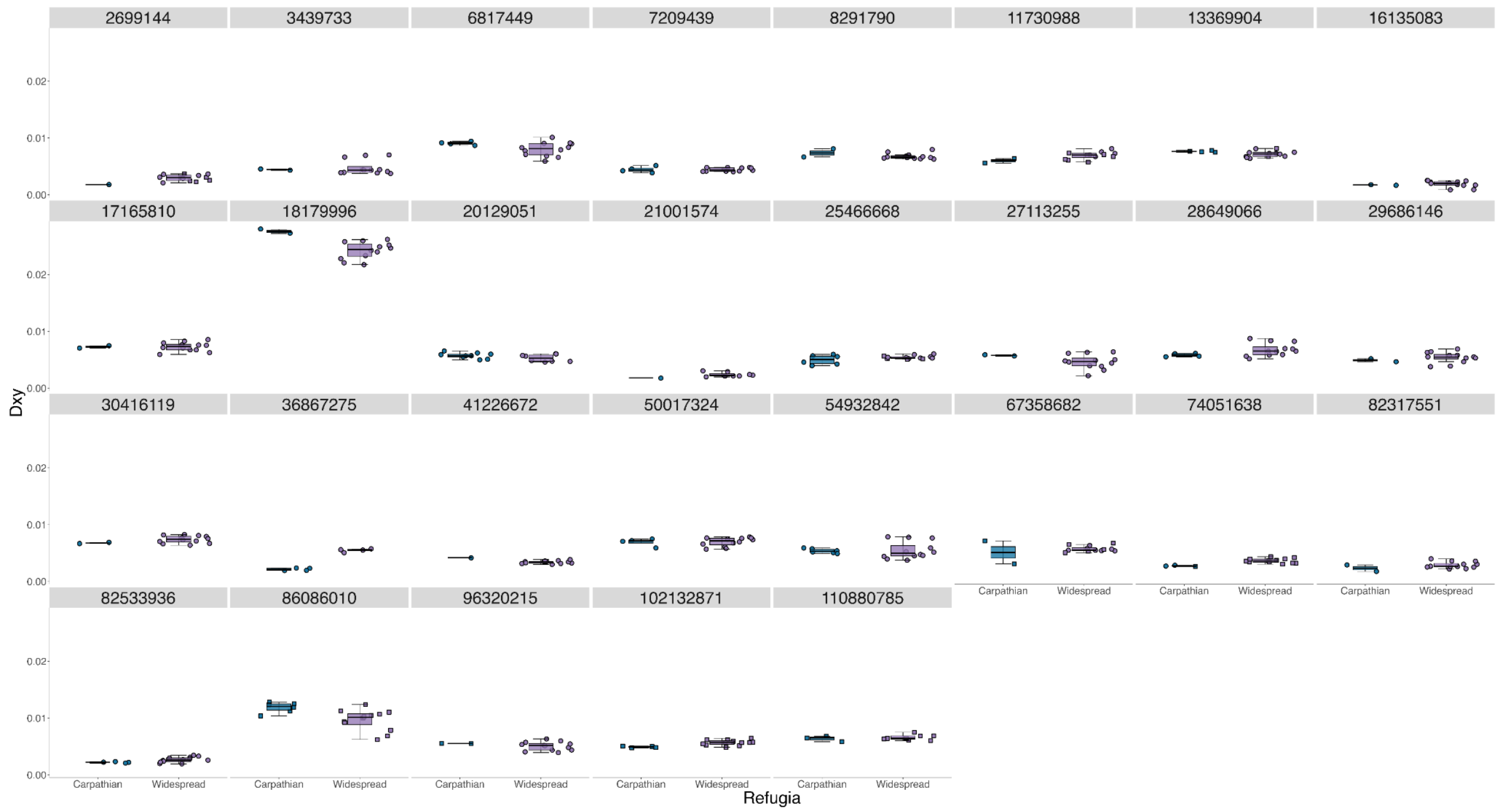

Supplementary Figure 2: Divergence (Dxy) between populations for Carpathian and widespread alleles, organized by candidate locus. Each panel is labeled with the genomic position of the locus, and points represent pairwise comparisons among all populations carrying that haplotype.

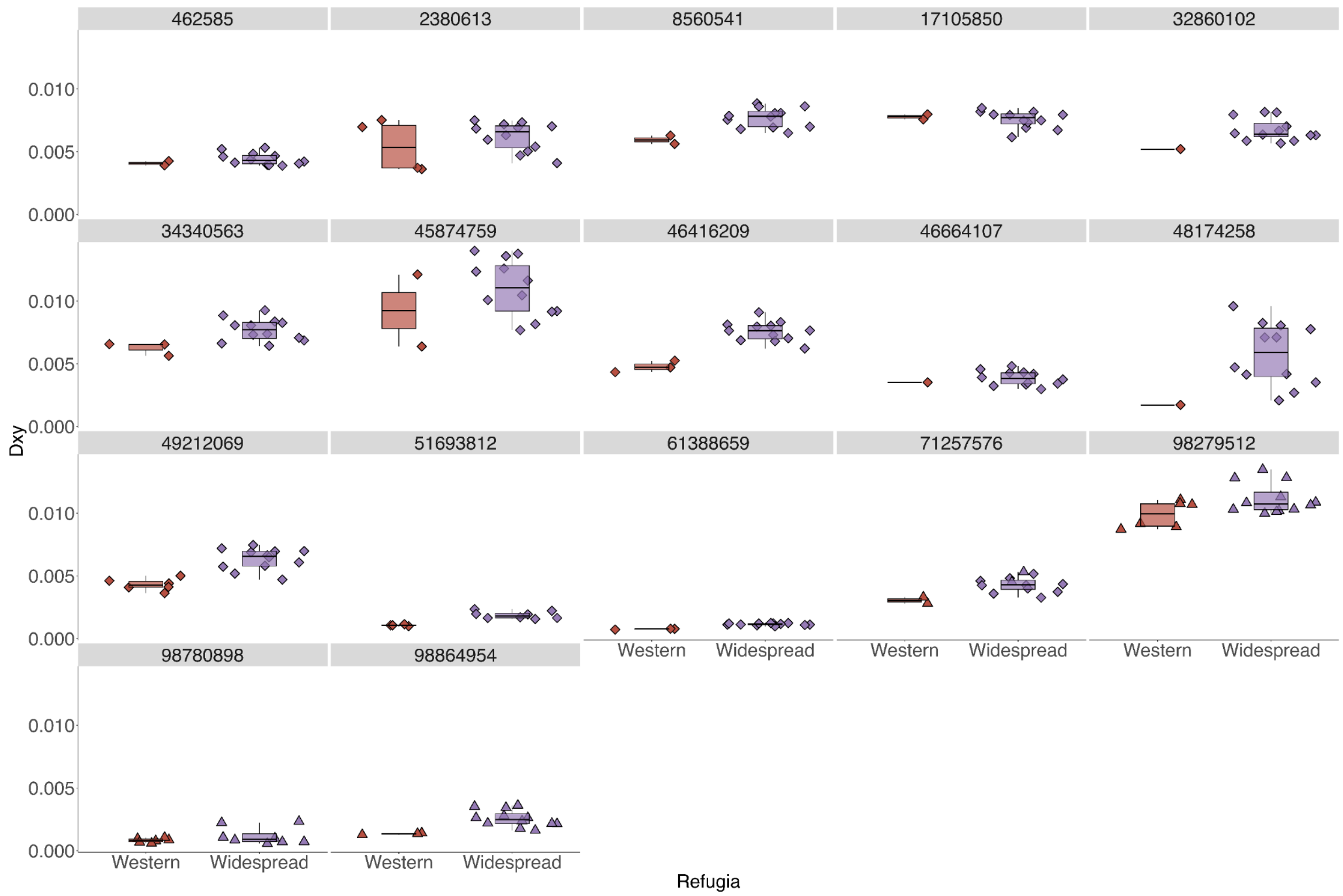

Supplementary Figure 3: Divergence (Dxy) between populations for Western and widespread alleles, organized by candidate locus. Each panel is labeled with the genomic position of the locus, and points represent pairwise comparisons among all populations carrying that haplotype.

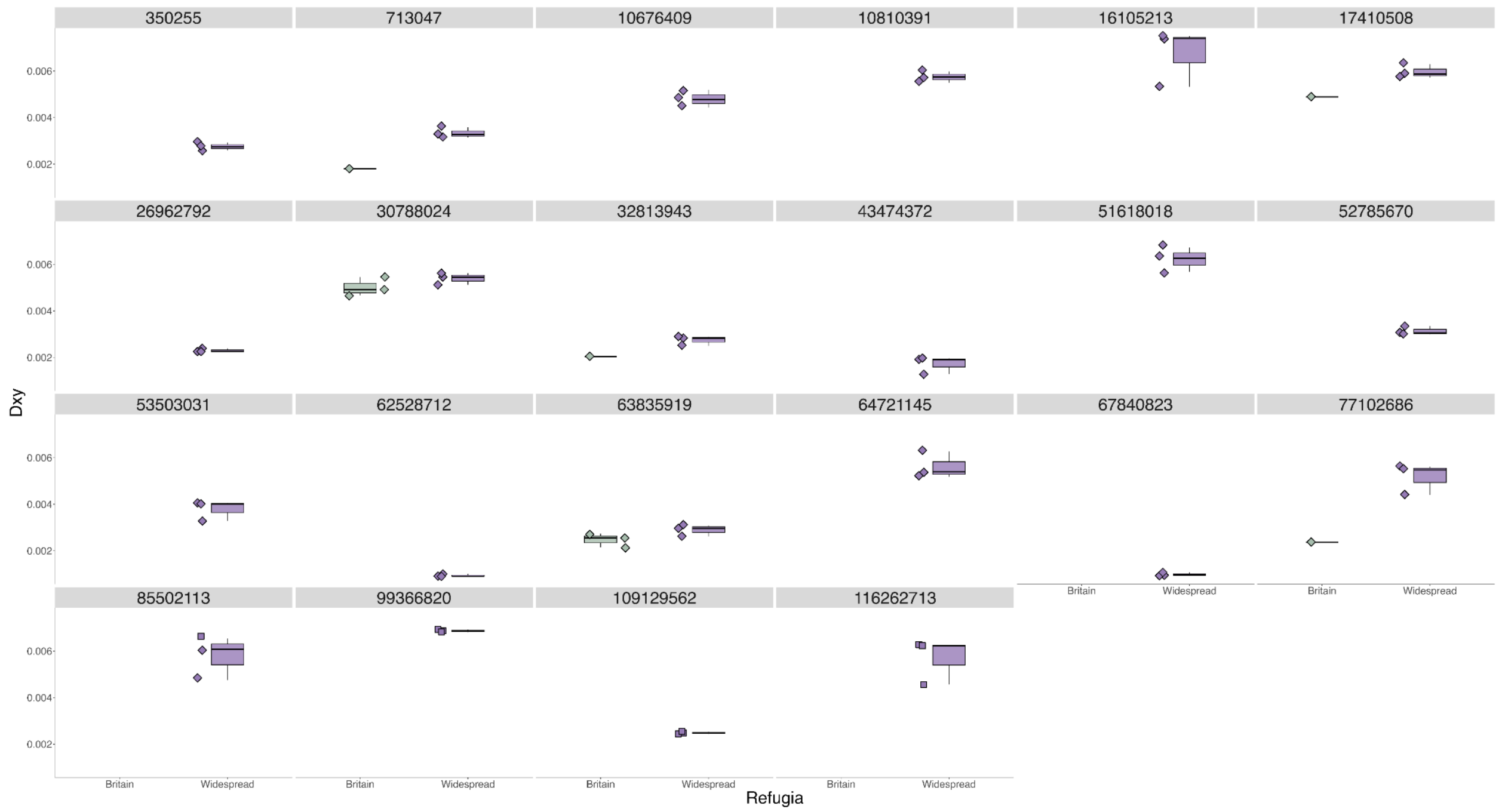

Supplementary Figure 4: Divergence (Dxy) between populations for British and widespread alleles, organized by candidate locus. Each panel is labeled with the genomic position of the locus, and points represent pairwise comparisons among all populations carrying that haplotype. Alleles occurring at only 1 of the 3 British populations have no divergence calculation, and only the divergence widespread alleles at the same locus are shown.

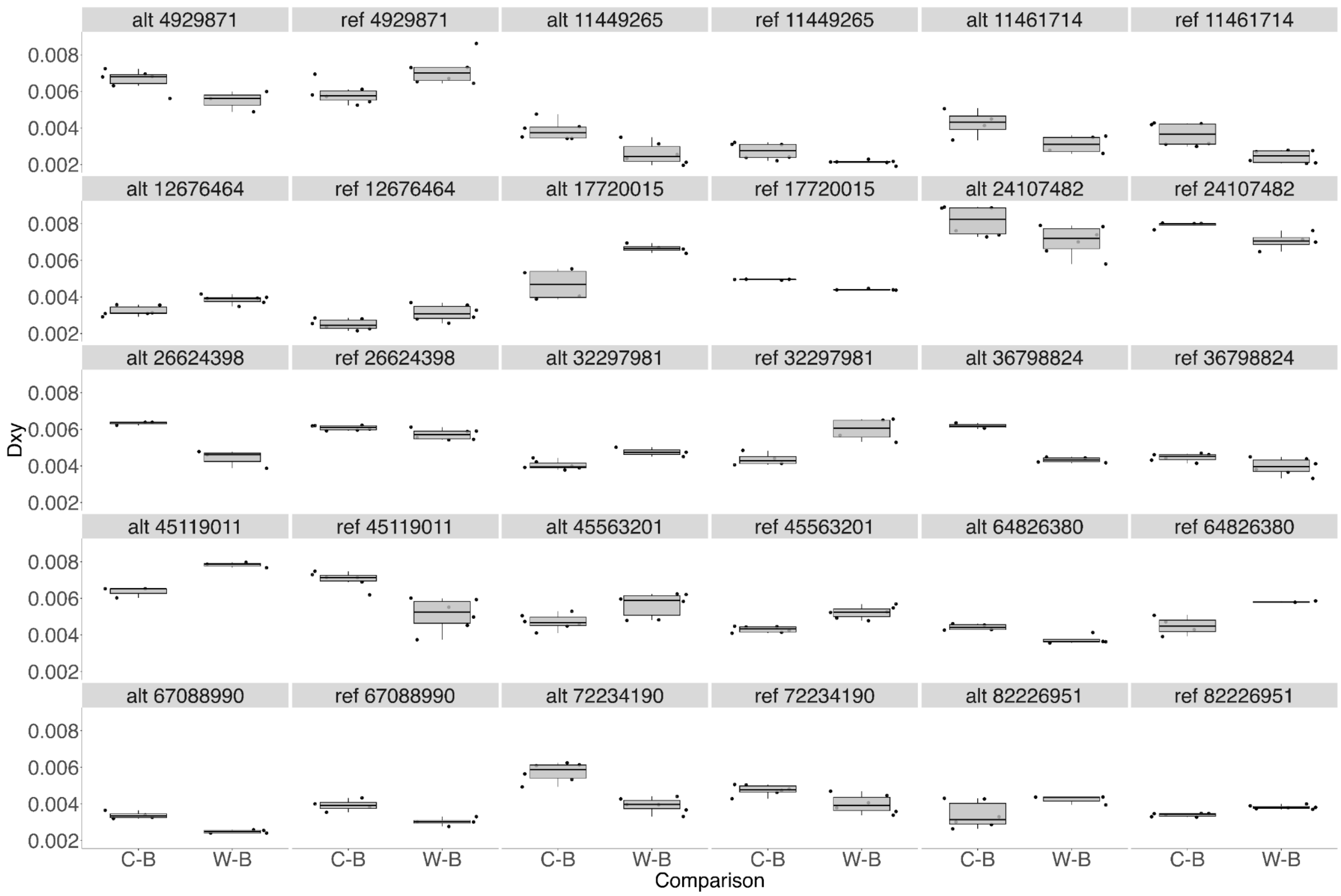

Supplementary Figure 5: Divergence (Dxy) between British and Carpathian (C-B) or British and Western (W-B) populations for both haplotypes at widespread polymorphic loci. Shown are widespread loci which exhibited significantly different levels of divergence between haplotypes (i.e., one haplotype is significantly more divergent than the other). Each comparison is labeled (above the graph) as alternative or reference, with respect to the bank vole genome, along with the position in the genome. Out of 1074 loci, only 15 exhibited haplotypes which had significantly different (*p*-value < 0.05) Dxy values when comparing C-B and W-B. Only four loci (492871, 17720015, 45119011 and 64826380) show a pattern of differential Dxy when comparing divergence with both mainland refugia (e.g., the alternative haplotype is more similar to the Western refugia but the reference haplotype is more similar to the Carpathian variant). We expect this differential Dxy if each lineage brought only one haplotype to Britain and the polymorphism recovered was due to admixture between lineages.

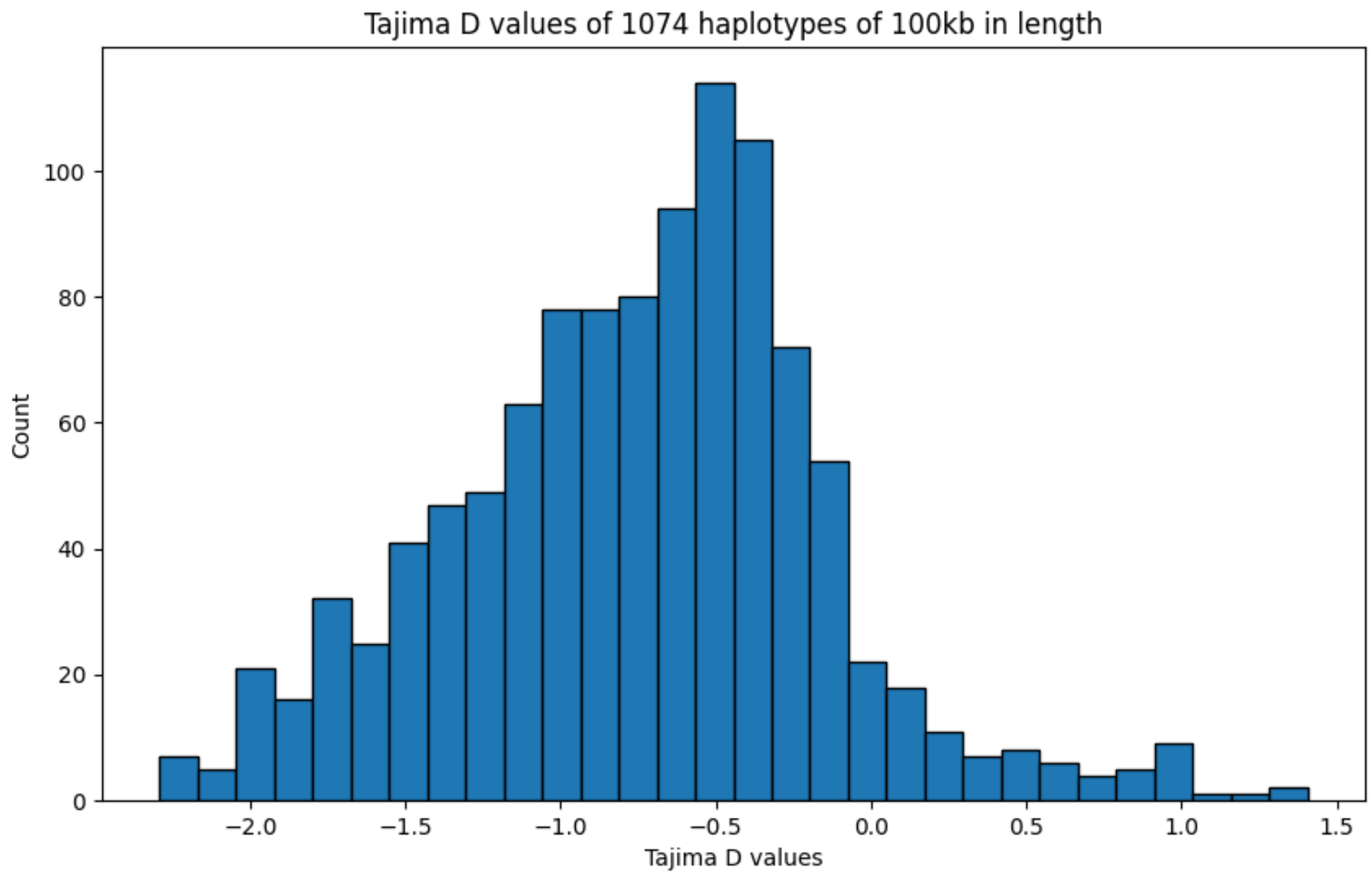

Supplementary Figure 6: The distribution of Tajima's *D* values across the 1074 haplotypes blocks of ~100 kb in length surrounding the neutral panel of loci.

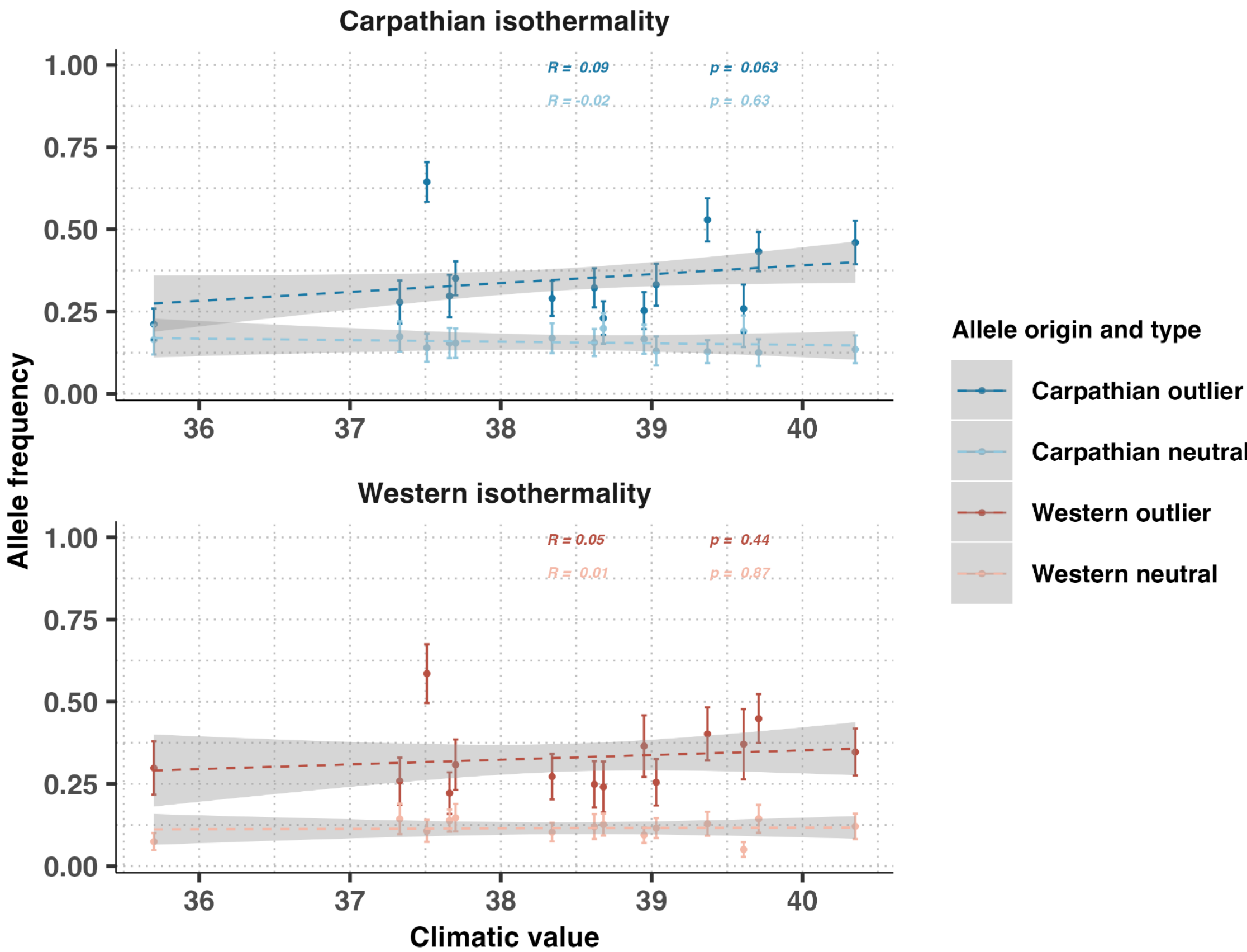

Supplementary Figure 7: Frequencies of alleles by category (i.e., outliers or neutral alleles associated with each origin) relative to isothermality across populations. While outlier alleles are typically at higher frequency than neural alleles, the relationship between isothermality and the overall allele frequency in a population does not differ for alleles from either refugium. Error bars represent standard error (*n* = 14).

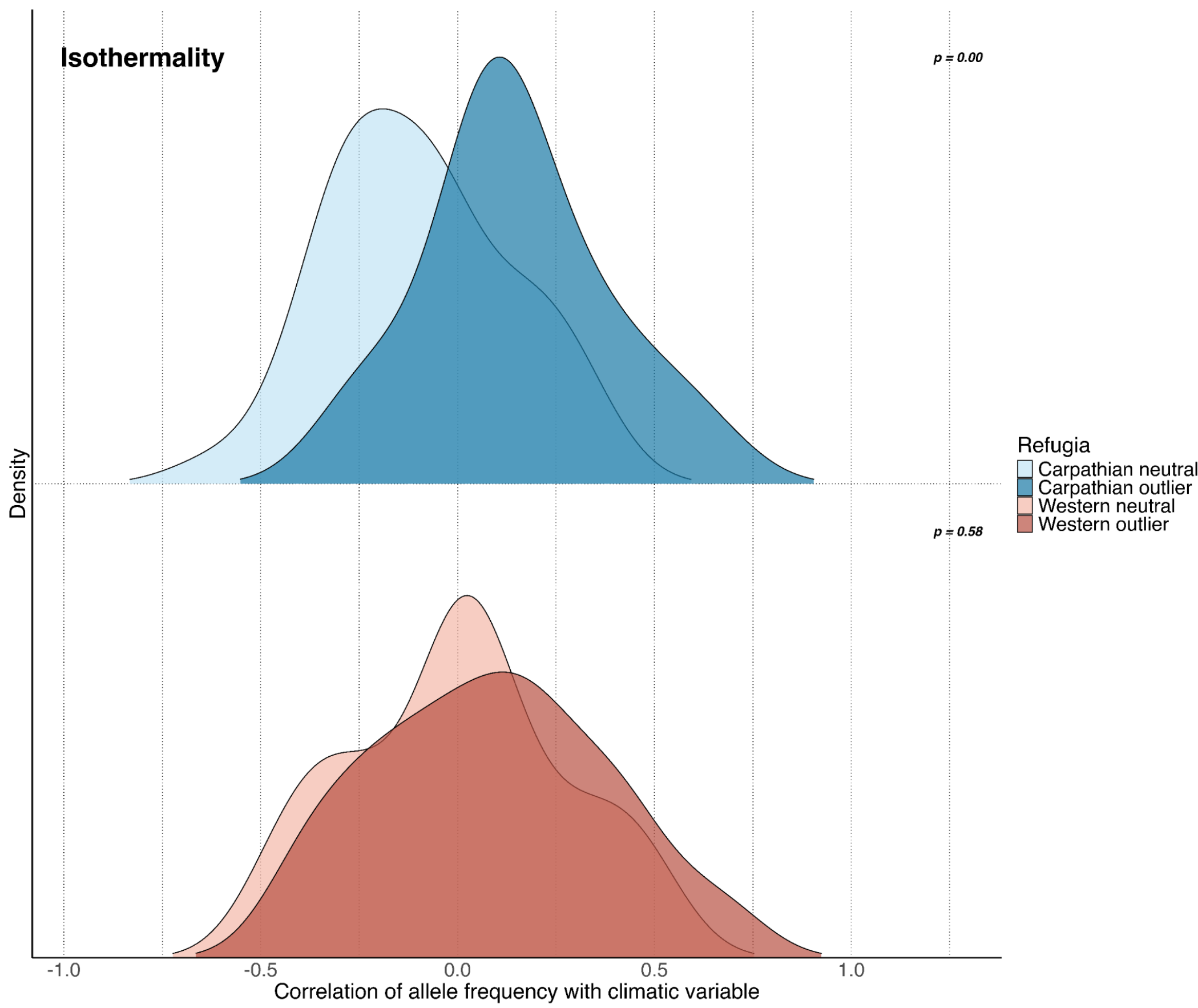

Supplementary Figure 8: The distributions of correlation coefficients between isothermality and the frequencies of outlier or neutral alleles calculated on a per-allele basis. Differences between the distributions of correlation coefficients for neutral and outlier alleles from each refugium were assessed using Kolmogorov–Smirnov tests.

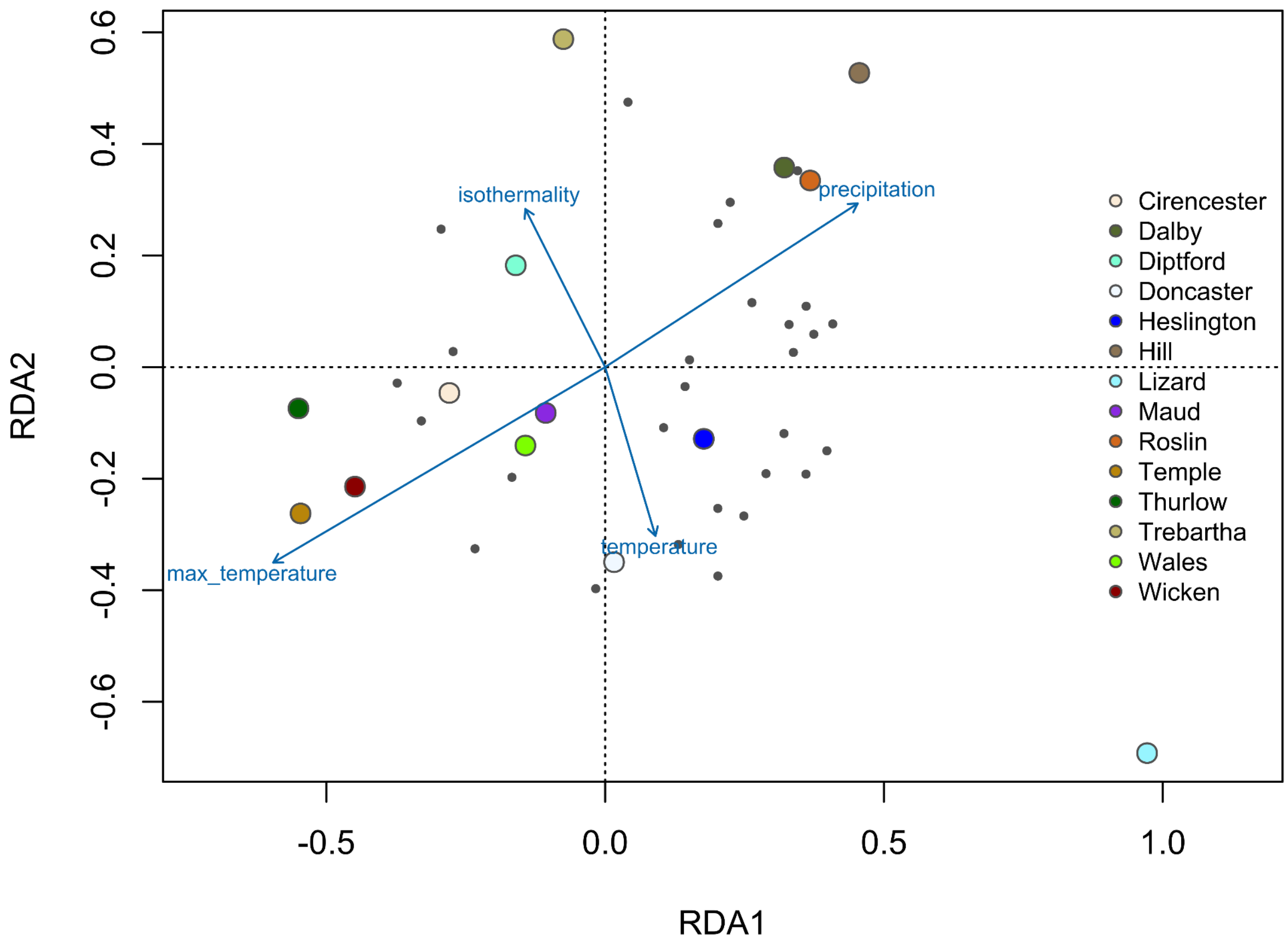

Supplementary Figure 9: Relationship between Carpathian allele frequency and climatic variables in British populations based on pRDA analyses. Gray crosses represent alleles, arrows represent predictor loading, and each British population is shown with a colored dot. There is a positive relationship between many of the Carpathian alleles and mean annual temperature (lower right quadrant) and precipitation (upper right quadrant), with a few alleles more closely associated with maximum temperature of the warmest month (lower left quadrant). The variation explained by RD1 and RD2 is 36% and 16%, respectively.

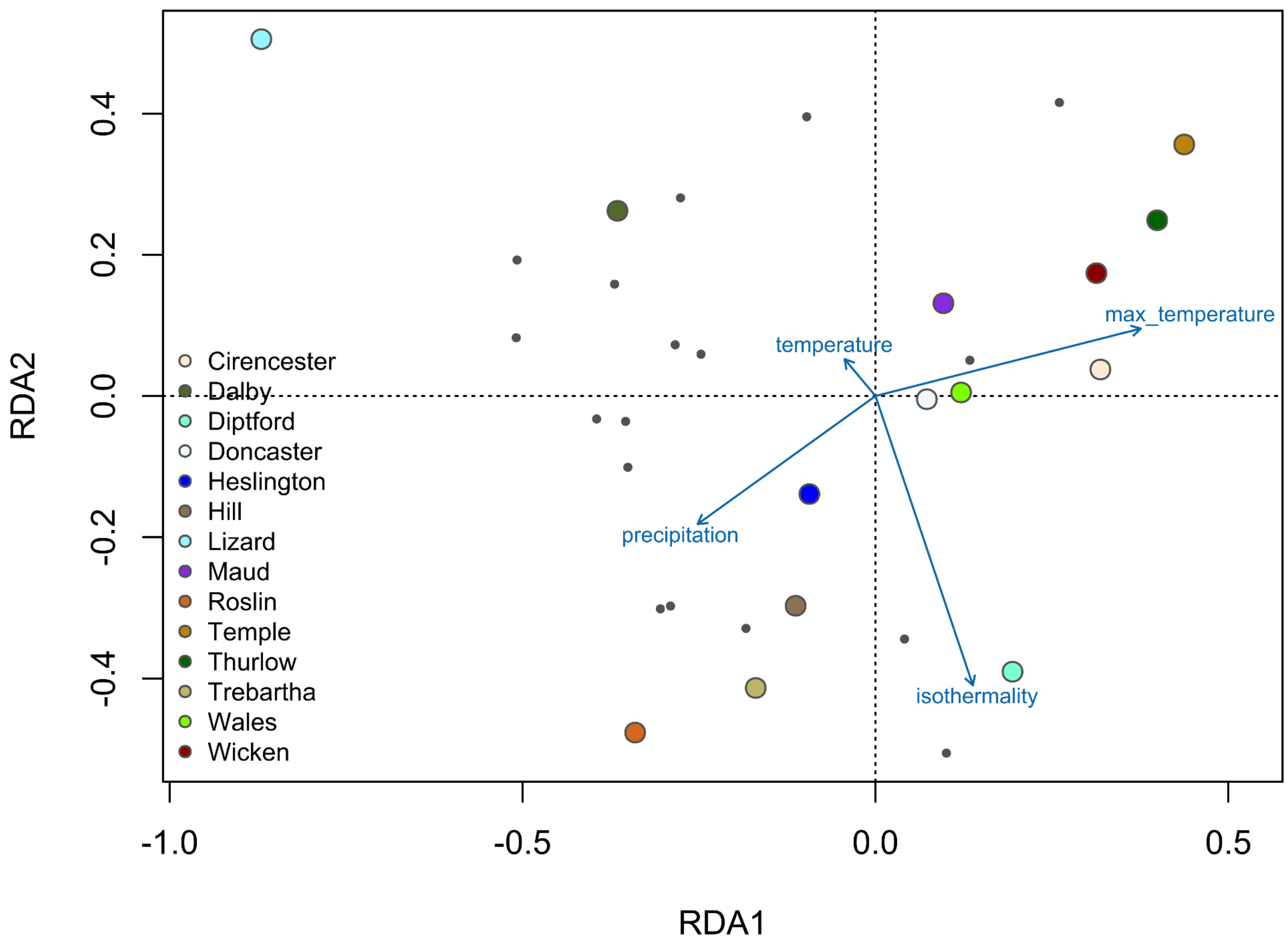

Supplementary Figure 10: Relationship between Western allele frequency and climatic variables in British populations based on the pRDA analyses. Gray crosses represent the alleles, arrows represent each predictor loading, and each British population is shown with a colored dot. There is a positive correlation between many of the Western alleles with mean annual temperature (upper left quadrant) and precipitation (lower left quadrants). The variation explained by RD1 and RD2 is 20% and 10%, respectively.
