# Supplementary material for: Origins of adaptive genomic variation in a wild rodent
Source: Commun Biol. 2026 Jun 26;9:1360. doi: 10.1038/s42003-026-10511-6 (PMC13677672; doi:10.1038/s42003-026-10511-6)
Supplement: Supplementary file 3 [file 42003_2026_10511_MOESM3_ESM.pdf]

Description of Additional Supplementary Files

File name: Supplementary Data 1

Description: Numerical source data for graphs and charts can be found in Supplementary Data 1.
