# Supplementary material for: Origins of adaptive genomic variation in a wild rodent
Source: Commun Biol. 2026 Jun 26;9:1360. doi: 10.1038/s42003-026-10511-6 (PMC13677672; doi:10.1038/s42003-026-10511-6)
Supplement: Supplementary file 4 [file 42003_2026_10511_MOESM5_ESM.pdf]

nature portfolio

Corresponding author(s): Marcos da Cruz

Last updated by author(s): May 28, 2026

Reporting Summary

Nature Portfolio wishes to improve the reproducibility of the work that we publish. This form provides structure for consistency and transparency in reporting. For further information on Nature Portfolio policies, see our Editorial Policies and the Editorial Policy Checklist.

Statistics

For all statistical analyses, confirm that the following items are present in the figure legend, table legend, main text, or Methods section.

n/a	Confirmed	
☐	☒	The exact sample size (*n*) for each experimental group/condition, given as a discrete number and unit of measurement
☐	☒	A statement on whether measurements were taken from distinct samples or whether the same sample was measured repeatedly
☐	☒	The statistical test(s) used AND whether they are one- or two-sided<br>*Only common tests should be described solely by name; describe more complex techniques in the Methods section.*
☒	☐	A description of all covariates tested
☒	☐	A description of any assumptions or corrections, such as tests of normality and adjustment for multiple comparisons
☐	☒	A full description of the statistical parameters including central tendency (e.g. means) or other basic estimates (e.g. regression coefficient) AND variation (e.g. standard deviation) or associated estimates of uncertainty (e.g. confidence intervals)
☐	☒	For null hypothesis testing, the test statistic (e.g. *F*, *t*, *r*) with confidence intervals, effect sizes, degrees of freedom and *P* value noted<br>*Give P values as exact values whenever suitable.*
☒	☐	For Bayesian analysis, information on the choice of priors and Markov chain Monte Carlo settings
☒	☐	For hierarchical and complex designs, identification of the appropriate level for tests and full reporting of outcomes
☒	☐	Estimates of effect sizes (e.g. Cohen's *d*, Pearson's *r*), indicating how they were calculated

Our web collection on statistics for biologists contains articles on many of the points above.

Software and code

Policy information about availability of computer code

Data collection	The present study did not use algorithms or software that are central to the research but have not yet been described in the published literature.
Data analysis	Data were processed and analyzed with bcl2fastq v2.20.0.422, BCFtools v.1.9, and VCFtools v.0.1.16, PLINK v.2.0, BEAGLE v.5.4, vcflib v1.0.9, RAxML v.9.2.4, ggtree v3.5.3, RUNTC v.0.0.1, PIXY v1.2.7, and vegan v2.6.4. Code used for converting and visualizing data and running analyses in R is available in GitHub (https://github.com/marcticruz/Origins-of-adaptive-genomic-variation-in-a-wild-rodent).

For manuscripts utilizing custom algorithms or software that are central to the research but not yet described in published literature, software must be made available to editors and reviewers. We strongly encourage code deposition in a community repository (e.g. GitHub). See the Nature Portfolio guidelines for submitting code & software for further information.

Data

Policy information about availability of data

All manuscripts must include a data availability statement. This statement should provide the following information, where applicable:

- Accession codes, unique identifiers, or web links for publicly available datasets
- A description of any restrictions on data availability
- For clinical datasets or third party data, please ensure that the statement adheres to our policy

The SNP dataset can be accessed through the Dryad Digital Repository (https://datadryad.org/dataset/doi:10.5061/dryad.k3j9kd5qq), raw sequence data are

available at NCBI in the Sequence Read Archive (SRA) under BioProject PRJNA1097528, and summarized data products used for plotting figures is accessible in the Supplementary Data file uploaded with the manuscript.

Research involving human participants, their data, or biological material

Policy information about studies with human participants or human data. See also policy information about sex, gender (identity/presentation), and sexual orientation and race, ethnicity and racism.

Reporting on sex and gender	This study does not include human research participants.
Reporting on race, ethnicity, or other socially relevant groupings	This study does not include human research participants.
Population characteristics	This study does not include human research participants.
Recruitment	This study does not include human research participants.
Ethics oversight	This study does not include human research participants.

Note that full information on the approval of the study protocol must also be provided in the manuscript.

Field-specific reporting

Please select the one below that is the best fit for your research. If you are not sure, read the appropriate sections before making your selection.

☐ Life sciences ☐ Behavioural & social sciences ☒ Ecological, evolutionary & environmental sciences

For a reference copy of the document with all sections, see nature.com/documents/nr-reporting-summary-flat.pdf

Ecological, evolutionary & environmental sciences study design

All studies must disclose on these points even when the disclosure is negative.

Study description	Whole-genome sequencing data were used to identify the origins of variation at putative adaptive loci (outlier loci, identified in a prior study) for the bank vole in Britain. These analyses were paired with estimates of allele age and climate associations to identify the role of gene flow and genomic ancestry in shaping local adaptation patterns.
Research sample	The present manuscript whole genome data for bank voles (Clethrionomys glareolus) derived from a previous study (Marková et al., 2023,, Nature Communications) as well as newly generated whole genome data from populations associated with the European refugia which gave rise to the British lineages and a Mediterranean population as an outgroup. In that earlier study, we used the full dataset to determine the capability of local populations to adapt to current and future climatic conditions and identify targets of selection. Here we build upon those findings by identifying the origins of alleles.
Sampling strategy	Data from the Markova et al. 2023 study involve the sample size was set according to the available samples, aiming for a number of 10 individuals per population to ensure a balance between the number of individuals and localities. In order to minimize bias, we used similar sample sizes from European populations.
Data collection	Genomic DNA was extracted from previously available tissue samples (liver, spleen, and toes, see Marková et al. 2021) using the DNeasy kit and whole-genome libraries were prepared and sequenced from those samples at the University of Oklahoma.
Timing and spatial scale	Sequence data are from a previous study (see above), where they were obtained from available bank vole samples collected mainly between 2009 and 2011. Whole genome libraries for the Markova et al. mansucript were generated in Summer 2020 and the genomic data added for this manuscript were generated in summer 2021.
Data exclusions	SNPs were filtered to remove loci with low coverage and quality and a high proportion of missing genotypes.
Reproducibility	Haplotype trees were independently scored by two of the authors to ensure convergence in the coding.
Randomization	We randomly sampled neutral background loci from throughout the genome, excluding all regions which were within 50kb on either side of putative adaptive loci.
Blinding	Blinding practice was not relevant to our study because no trials were performed.

Did the study involve field work? ☐ Yes ☒ No

Reporting for specific materials, systems and methods

We require information from authors about some types of materials, experimental systems and methods used in many studies. Here, indicate whether each material, system or method listed is relevant to your study. If you are not sure if a list item applies to your research, read the appropriate section before selecting a response.

Materials & experimental systems

n/a	Involved in the study
☒	☐ Antibodies
☒	☐ Eukaryotic cell lines
☒	☐ Palaeontology and archaeology
☒	☐ Animals and other organisms
☒	☐ Clinical data
☒	☐ Dual use research of concern
☒	☐ Plants

Methods

n/a	Involved in the study
☒	☐ ChIP-seq
☒	☐ Flow cytometry
☒	☐ MRI-based neuroimaging

Plants

Seed stocks	Not applicable.
Novel plant genotypes	Not applicable.
Authentication	Not applicable.
